# GenePattern flow cytometry suite

**DOI:** 10.1186/1751-0473-8-14

**Published:** 2013-07-03

**Authors:** Josef Spidlen, Aaron Barsky, Karin Breuer, Peter Carr, Marc-Danie Nazaire, Barbara Allen Hill, Yu Qian, Ted Liefeld, Michael Reich, Jill P Mesirov, Peter Wilkinson, Richard H Scheuermann, Rafick-Pierre Sekaly, Ryan R Brinkman

**Affiliations:** 1 Terry Fox Laboratory, British Columbia Cancer Agency, Vancouver, BC, Canada; 2 Department of Molecular Biology and Biochemistry, Simon Fraser University, Burnaby, British Columbia, Canada; 3 Computational Biology and Bioinformatics, Broad Institute of MIT and Harvard, Cambridge, MA, USA; 4 Vaccine and Gene Therapy Institute of Florida, Port Saint Lucie, FL, USA; 5 J. Craig Venter Institute, San Diego, CA, USA; 6 Department of Medical Genetics, University of British Columbia, Vancouver, BC, Canada

**Keywords:** Flow cytometry, Data analysis, GenePattern, FCS, Data preprocessing, Quality assessment, Normalization, Clustering

## Abstract

**Background:**

Traditional flow cytometry data analysis is largely based on interactive and time consuming analysis of series two dimensional representations of up to 20 dimensional data. Recent technological advances have increased the amount of data generated by the technology and outpaced the development of data analysis approaches. While there are advanced tools available, including many R/BioConductor packages, these are only accessible programmatically and therefore out of reach for most experimentalists. GenePattern is a powerful genomic analysis platform with over 200 tools for analysis of gene expression, proteomics, and other data. A web-based interface provides easy access to these tools and allows the creation of automated analysis pipelines enabling reproducible research.

**Results:**

In order to bring advanced flow cytometry data analysis tools to experimentalists without programmatic skills, we developed the GenePattern Flow Cytometry Suite. It contains 34 open source GenePattern flow cytometry modules covering methods from basic processing of flow cytometry standard (*i.e.*, FCS) files to advanced algorithms for automated identification of cell populations, normalization and quality assessment. Internally, these modules leverage from functionality developed in R/BioConductor. Using the GenePattern web-based interface, they can be connected to build analytical pipelines.

**Conclusions:**

GenePattern Flow Cytometry Suite brings advanced flow cytometry data analysis capabilities to users with minimal computer skills. Functionality previously available only to skilled bioinformaticians is now easily accessible from a web browser.

## Background

### Flow cytometry

Flow cytometry (FCM) is a technique for counting and examining microscopic particles, such as cells, by suspending them in a stream of fluid and passing them individually past a detector. It allows the simultaneous multi-parametric analysis of the physical and chemical characteristics of up to thousands of particles per second. For more than 30 years, FCM has been widely used by clinicians, immunologists, and cancer biologists to distinguish different cell types in mixed cell sub-populations, based on the expression of cellular markers. In both health research and treatment this analytical method is used for a variety of tasks, in particular the diagnosis and monitoring of HIV infection and cancer, cross-matching organs for transplantation, and for research involving stem cells, vaccine development, apoptosis and phagocytosis.

In the last decade, advances in FCM instrumentation and reagent technologies have enabled simultaneous single cell measurement of surface and intracellular markers, including cellular-activation markers, intra-cellular cytokines, immunological signaling, and cytoplasmic and nuclear cell cycle and transcription factors, thus positioning FCM to play an even bigger role in health care and medical research [1-3]. Today’s flow cytometers can measure up to 20 parameters simultaneously – two physical parameters (cell size and granularity) and 18 fluorescent markers [4]. However, the rapid development of FCM instrumentation has outpaced the development of adequate approaches and tools for data analysis. Traditionally, the majority of FCM experiments have been analyzed visually, through time-consuming and subjective serial inspection of one or two dimensions at a time (using a process termed “gating”, to define boundaries or “gates” for cell sub-populations), or by very basic comparisons of summary statistics. With 20 dimensional data, there are hundreds of possible pairwise parameter combinations and many cell populations. This number increased with the recent introduction of CyTOF instruments allowing potentially up to 100 stable isotope labels in a single sample and creating up to 100 dimensional FCS data files [5]. Analyzing such complex data is time consuming and human experts can miss important cell populations if these are only visible in high dimensional space not clearly distinguishable in any of the pairwise plots. For these reasons, there has been recent interest in developing new data analysis techniques that will exploit the full potential of modern flow cytometers and provide standardized, reproducible and objective analyses [6,7]. These are often created in the form of programming libraries, such as R/BioConductor [8] packages, and therefore only accessible to sophisticated users rather than experimentalists who lack advanced programming skills.

### GenePattern

GenePattern is a powerful web-based application offering easy access to over 180 tools for analysis of gene expression, proteomics, and other data [9]. An additional 100 tools are under development and testing at this point. These tools are provided in the form of modules, typically written in R, Java, Matlab, or Perl. GenePattern was originally released in 2004 and now has more than 22,000 users world wide. Using a web-based interface, experimentalists can easily submit their data and choose suitable settings in order to perform complex analyses without detailed knowledge of the underlying programming language, algorithms and settings, allowing them to concentrate on the interpretation of biologically meaningful results. Besides executing various modules as standalone tools, users can also chain modules together to create automated analysis pipelines enabling reproducible in silico research, now also facilitated by a Microsoft Word add-in as part of the GenePattern Reproducible Research Document that allows scientists to embed their pipelines in a text document [10].

## Methods

GenePattern is a web-based tool running within an Apache Tomcat application server. The GenePattern Flow Cytometry Suite (GP FCM Suite) is implemented as a set of GenePattern modules. These are command line-based software applications with formally defined syntax, inputs and outputs. These definitions reside in a manifest file, packaged together with the source code of the module and documentation in a module ZIP archive. The GP FCM Suite modules are developed in R, Java and C. The R-based modules extensively reuse many existing flow cytometry related R/BioConductor packages [11-18]. Java-based modules reuse a Java library called CFCS, which is an open-source implementation of the *Proposed API for reading and writing FCS files*[19]. The CFCS library was originally developed in 2003 by Tree Star, Inc. (Ashland, OR) and is now maintained by our group at the British Columbia Cancer Agency (BCCA) and is available from the *flowcyt* website [20].

## Results and discussion

**Figure 1:**
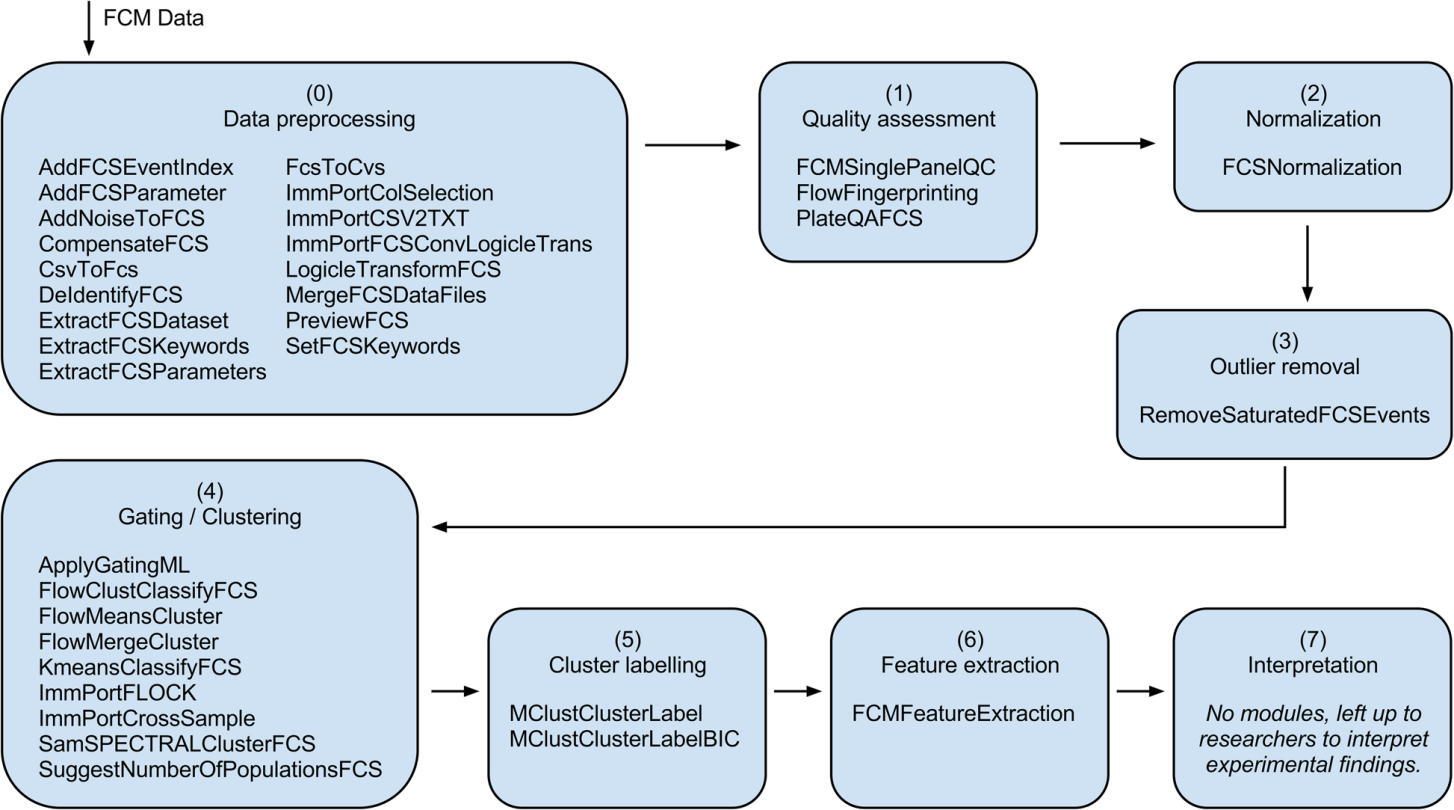
**GP FCM Suite Modules Overview.** Figure 1 enumerates modules currently available in the GP FCM Suite. These modules are assigned to steps (0–7) based on which step they address according to the general automated data analysis pipeline. This pipeline is based on framework [7] with an initial step (0) added for data preprocessing and with gating extended to cover both, manual and automated approaches.

We previously proposed a general FCM data analysis framework [7] consisting of seven steps: (1) *Quality assessment*, (2) *Normalization*, (3) *Outliers removal*, (4) *Automated gating*, (5) *Cluster labelling*, (6) *Feature extraction* and (7) *Interpretation*. Except for interpretation, the GP FCM Suite addresses all these steps; commonly with multiple modules and approaches (Figure 1). The last step – interpretation – is highly dependent on the actual experiment type (design, hypothesis, type of clinical test, etc.). Therefore, we currently leave it up the the user to choose an appropriate approach to interpret experimental findings. In addition to the proposed framework, the GP FCM Suite contains several *Data preprocessing* modules often required before the start of data analysis. Finally, manual gating was not considered in the general automated FCM data analysis framework [7]. While we do not incorporate interactive manual gating in automated analysis pipelines, we still allow users to reuse results of manual gating for the analysis in GenePattern.

### Data preprocessing

The GP FCM Suite includes several data preprocessing steps such as data preview and transformations, conversion between spreadsheets (i.e., CSV files) and the Flow Cytometry data file Standard (FCS [21]), merging and sub-sampling data as described below and shown in Figure 1, step 0.

#### Data preview

The first essential step in data processing is commonly the review of the contents of an FCS data file. This becomes especially important if a user is not familiar with the details about the data. The GP FCM Suite provides a data preview module, which lists the meta information stored in the file and provides details such as the number of events in the file (i.e., the number of particles, such as cells, whose characteristics have been captured in the file) and the number of parameters in the file (i.e., the number of distinct characteristics measured). Output is available as either as an HTML report (for human review) or an XML document (for further automated processing).

#### Adding and removing FCS keywords and parameters

We provide functionality for editing, adding or removing keyword/value pairs stored in the meta data section of FCS files (e.g., for de-identification of clinical data prior to sharing). In addition, we also offer modules to add or remove FCM parameters from data files. Adding a parameter is useful, for example, if calculated event (cell) features need to be stored. These may include assignments of cells into subpopulations as the result of a clustering algorithm. Removing parameters is useful for high content experiment with many markers where only a subset is included in a manuscript.

#### Adjusting data scale

In most FCM applications, fluorescence signals of interest can range over several decades. Several transformations have been developed to provide more complete, appropriate, and readily interpretable representations. Via a dedicated module (*LogicleTransformFCS*), the GP FCM Suite includes support for the Logicle / Biexponential [22,23] data transformation, the de-facto standard for contemporary visualization of FCM data. Additional transformation are supported via Gating-ML [24], including both FCM-specific transformations (e.g., Hyperlog [25], Split-scale [26]) as well as more generic transformations (e.g., inverse hyperbolic sine, logarithmic).

#### Data merging and sub-sampling

Merging multiple data sets into a single file can be used to identify all the cell subpopulations that are present among a group of subjects. We have also included several options to sub-sample the data since including all events from all source files creates a file whose size approximately equals to the sum of sizes of all source files, which may become too large to process by some algorithms.

#### Conversion to and from spreadsheets

Converting between FCS files and simple spreadsheets (*i.e.*, CSV files) becomes important if part of an analysis relies on customized tools that handle simple matrix-based data but do not implement the parsing of the FCS format. The GP FCM Suite includes modules for conversion in both directions and provides additional options allowing the specification of the details of how data shall be stored, such as the data type, range, precision, etc.

#### Compensation

Compensation is the process whereby the fluorescence spillover originating from a fluorochrome other than the one specified for a particular detector is subtracted as a percentage of the signal from other detectors. The inherent overlap of emission spectra from antibody fluorescent labels makes compensation necessary before proceeding with further analysis. The GP FCM Suite provides the ability to perform compensation based on the fluorescence spillover matrix that is commonly included within FCS files, or using compensation specification supplied externally.

#### Adding noise

The GP FCM Suite also offers a way to add noise to data sets, mainly to aid automated clustering methods as some these are sensitive to data singularity and may fail to deliver results unless a small amount of noise is added [27].

### Quality assessment

Data quality assessment (Figure 1, step 1) represents an important part of any data analysis, and quality control tests should be included at the beginning of data analysis and often at other steps of an analytical pipeline to identify differences in samples originating from changes in conditions that are probably not biologically motivated. Generally, methods establish a quality control criterion to give special consideration to abnormal samples or even exclude these from further analysis.

Quality control tests in the GP FCM Suite are largely based on functionality implemented in the *flowQ*[13]*R/BioConductor* package. They include tests applicable to both, plate-based and single panel FCM data (e.g., cell number test, time flow test, Probability Density Function (PDF) and medians test of forward and side scatter for cell debris). An interactive HTML report is created after the execution of selected quality assessment tests displaying an overview table with rows corresponding to tested samples and columns to selected quality control tests. The results of these tests are color-coded with green indicating no problems, yellow indicating a warning, and red suggesting the failure of a certain test on a certain sample. Clicking on the heading shows an overview plot for that particular test, and clicking on a particular sample/test result will reveal details about the execution of that test on that sample. It is left up to the user to review flagged samples and exclude individual samples from further analysis as appropriate. An example of a quality assessment report of a 96 well plate of a “Normal Donor” study performed by Becton, Dickinson and Company (BD) in order to measure immune responses to various infectious agents and cancer antigens among healthy young adults is included as Additional file 1.

#### Fingerprinting

Fingerprinting generates a description of the multivariate probability distribution function of FCM data by transforming raw FCM data into a fingerprint form suitable for data quality assessment purposes as well as direct input into conventional statistical analysis and empirical modeling software tools. Fingerprinting is independent of a presumptive functional form for the distribution, in contrast with model-based methods such as Gaussian Mixture Modeling. Within GenePattern, we implement FCM fingerprinting functionalities based on the flowFP [28] R/BioConductor package. This approach is computationally efficient and able to handle large flow cytometry data sets of arbitrary dimensionality.

### Normalization

Between-sample variation in high throughput FCM data represents a significant challenge for analysis of large scale data sets, such as those derived from multi-center clinical trials. It is often hard to match biologically relevant cell populations across samples due to technical variation in sample acquisition and instrumentation differences. Thus, normalization of data is a critical step prior to analysis, particularly in large-scale data sets from clinical trials, where group specific differences may be subtle and patient-to-patient variation common. The GP FCM Suite includes a normalization method that removes technical between-sample variation by aligning prominent features (landmarks) in the raw data on a per-channel basis as described by Hahne et al. [14].

### Outliers removal

Before further analysis, users may want to perform initial data clean up, such as the removal of margin channel events. These may, for example, occur when the instrument detector voltages are set too high so that cells highly expressing certain markers create signals above the recordable range for corresponding parameters. Events created by these cells will condense at the parameter top range value and eventually create artificial cell populations, which may cause problems for further analysis. The GP FCM Suite offers a module for data clean up, including the removal of saturated events and events believed to be caused by instrument errors (Figure 1, step 3).

### Gating

Gating is an inherent component of FCM data analysis; it is a process where particles (i.e., cells) are subsetted according to physical and fluorescence characteristics. These properties are reflected in parameter values of events stored in FCS files. In practice, gating corresponds to assigning classes (labels) to these events. This can be done either manually or automatically. While manual gating is still dominant in traditional FCM, automatic gating methods are becoming more important in contemporary and high throughput approaches [6]. The GP FCM Suite supports both (Figure 1, step 4), as described below.

#### Manual gating

Manual gating involves a combination of biological domain knowledge and visual inspection of the data. Typically, gate boundaries are drawn interactively on series of one or two dimensional data projections. Within the GP FCM Suite, we do not support interactive manual gating as virtually all experimentalists performing manual gating use one of several commercially available software tools well suited for this purpose already. However, the GP FCM Suite still supports non-interactive manual gating based on the input of Gating-ML [24] files, an open XML-based standard for encoding gating and data transformations.

#### Automated gating (clustering)

Recently, several methods have been developed to automate the gating process [15-18,29-35]. These include both model-based methods, such as multivariate mixture models, as well as non-parametric approaches. One of these methods [33] has already been made available by their authors in the form of a GenePattern module, and we have added support for flowClust [15], flowMerge [16], K-Means [36], flowMeans [18], SamSPECTRAL [17] and FLOCK [34,35].

##### Number of sub-populations

Most clustering algorithms require some user input, such as the number of expected sub-populations to search for. Computationally, the estimation of the correct number of sub-populations present in a data set is difficult and may not only depend on the data but also on the goal of particular analysis. For example, cells that could be considered as outliers in one case, could also represent a rare population that may be important for classification of a certain disease, or they could indicate other useful information about the subject. Therefore, in the GP FCM Suite, we typically do not integrate the automated selection of the number of sub-populations in most clustering algorithms. Instead, we provide a separate module that investigates the data and suggests the number of sub-populations to the user. This is graphically supported by the output of the Baysian Information Criterion (BIC) and the Integrated Completed Likelihood (ICL) score for a range of sub-population numbers. Generally, these curves show how well the data can be modeled as a mixture of a certain number of populations. This approach has the advantage that the user may either accept the suggested value or select her/his own value based on prior knowledge and/or inspection of the BIC and/or ICL curves.

### Cluster labeling

Independent clustering of multiple flow cytometry samples (e.g., from different patients) results in dividing each of the input data files into several subsets corresponding to cell sub-populations in each of the particular sample. Another analytical step (i.e., Figure 1, step 5) is required to match (label) these sub-populations across different samples. This label matching is usually performed by comparison of the position of each of the identified sub-populations. In the GP FCM Suite, we offer modules for assigning labels to previously clustered data sets from multiple flow cytometry samples. The data may have been previously clustered by any clustering algorithm. The cluster matching is performed using model-based clustering of the means of the previously clustered sub-populations and users can choose from several models to fit their data. In addition, the user shall specify how many distinct sub-populations are expected to be found across all the previously clustered FCM data. Similar to estimating the number of sub-populations in a single sample, there is also a module in the GP FCM Suite that can help with this estimation across multiple samples.

### Feature extraction

The extraction of features (Figure 1, step 6) of identified sub-populations typically follows after gating and eventually labeling of FCM data. The main feature is simply the number (or proportion) of cells in different sub-population (e.g., how many cells are positive or negative for specific markers). In addition, one may be interested in the mean value of selected parameters (e.g., the mean fluorescence intensity – MFI – of a certain population of cells). The MFI can, for example, indicate the cellular response after specific antigen stimulation [37]. In the GP FCM Suite, we offer the calculation of cell number, proportion as well as mean parameter values. Other features that can be calculated include the integrated mean fluorescence intensity (iMFI) [38], obtained by multiplying the cell proportion by the mean fluorescence value.

### Interpretation

Interpretation of analytical results (Figure 1, step 7) is highly dependent on the actual experiment type, its design, the hypothesis being tested, the type of clinical test, etc. Therefore, it is likely impossible to create a generally applicable solution. In GenePattern, we created a few very specific modules to help researchers from BCCA test hypotheses related to their projects, such as the computational quantification of long-term reconstituting hematopoietic stem cells (HSC) from adult mouse bone marrow [39]. However, these modules rely on a very specific experimental design and tightly defined settings and protocols, and therefore, they are only useful for the laboratory they have been designed for. Consequently, we have not included these modules in the GP FCM Suite and we are leaving it up the researchers to decide about the best way to interpret their experimental findings.

### Availability and requirements

All GP FCM Suite modules are available from GenePattern ( http://www.genepattern.org); these can be run directly on the public server ( http://genepattern.broadinstitute.org/gp/) hosted at Broad Institute of MIT and Harvard or downloaded for use with own installation of GenePattern server. Currently available modules are also included in Additional file 2 and their source codes in Additional file 3. In addition, newest modules developed in the future may be accessed from the GenePattern beta server ( http://genepatternbeta.broadinstitute.org/gp/) before their official release through GenePattern. The GP FCM Suite is distributed as open source under the GNU LGPL 3.0 license. All used R libraries are freely available and their licensing conditions are specified on the download page of each specific library in the appropriate R repository, such as CRAN or BioConductor. GenePattern server software is freely available under the *GenePattern License Agreement*. The text of this license agreement is available at http://www.broadinstitute.org/cgi-bin/cancer/software/genepattern/gp_server_license.cgi.

## Conclusions

Traditional FCM data analysis involves the interpretation of individual two-dimensional scatter plots culled from sets of simultaneous analysis of highly multidimensional data. Recent technological advances have increased the amount of data generated by the FCM technology and outpaced the development of analytical approaches. While it is becoming clear that analysis methods based on manual gating are unsuitable for the increased amount of data and simultaneously measured fluorescence parameters, they still represent the main functionality in commercial FCM data analysis software. The need for new analytical approaches has been well recognized by the research community; however, advanced tools being developed are commonly released in the form of programming libraries (such as R/BioConductor packages) and therefore only accessible programmatically. Little effort is invested into making these available via user-friendly interfaces that would make these tools accessible for experimentalists without advanced programming skills.

In order to address this issue, we have developed the GP FCM Suite consisting of GenePattern modules to analyze FCM data. The modules in the GP FCM Suite can help with quality assessment, normalization, outliers removal, gating/clustering, cluster labeling, feature extraction and other tasks.

To the best of our knowledge, there is no other software tool that would provide a variety of advanced algorithms for the computational analysis of flow cytometry data via a user-friendly interface. However, a few software tools, most of them commercial, integrate one or two of these algorithms. For example, FlowJo ( http://www.flowjo.com/) allows users to utilize automated clustering for the purpose of analyzing flow cytometry data. Cytobank [40] has recently included the *Cyto Spanning tree Progression of Density normalized Events (SPADE)*[41] algorithm to their hosted versions of Cytobank and DVS Cytobank servers. The *Immunology Database and Analysis Portal* (ImmPort, https://immport.niaid.nih.gov) integrates the *FLOCK*[34,35] analysis (also available as part of the GP FCM Suite). GemStone (Verity Software House, http://www.vsh.com) offers a patented Probability State Modeling (PSM) technology to combine multiple samples and estimate missing parameter values. Finally, most of the major commercial third party software vendors, including Tree Star, De Novo Software, and Verity Software House, offer computational support for cell cycle analysis. All these tools integrate some algorithms facilitating users willing to apply computational methods for the analysis of flow cytometry data. While the scope and variety of implemented methods is limited compared to all the modules offered by the GP FCM Suite, the increasing commercial support clearly shows the new trend of users seeking advanced algorithms to help them analyze the increasing amount of increasingly complex data. Users with programmatic skills will always get the most out of the advanced FCM analysis tools if they programmatically incorporate these in an analysis pipeline. These users will have additional settings for various algorithms as well as the choice to encode more complex work flows compared to the options offered by GenePattern. However, we argue that most of the experimentalists have biology or medicine-related backgrounds and their programmatic skills are limited. For them, having advanced analytical functionality accessible from a simple web-based user interface becomes very useful.

## Abbreviations

BCCA: British Columbia Cancer Agency; BIC: Baysian information criterion; CSV: Comma-separated values; ECDF: Empirical cumulative distribution function; FCS: Flow cytometry standard; FCM: Flow cytometry; HTML: HyperText Markup Language; GP FCM Suite: GenePattern flow cytometry suite; HSC: Hematopoietic stem cells; ICL: Integrated completed likelihood; iMFI: Integrated mean fluorescence intensity; MFI: Mean fluorescence intensity; MIT: Massachusetts Institute of Technology; PDF: Probability density function; SVM: Support vector machine; XML: eXtensible Markup Language

## Competing interests

The authors declare that they have no competing interests.

## Authors’ contributions

JS led the module development effort, developed the majority of modules and wrote the initial version of the manuscript. AB, KB and PW contributed to module development. KB developed an automated module unit testing framework. PC and AB developed high throughput extension of GenePattern server. PC developed mechanism for resolving module dependencies on GenePattern server. YQ and RHS developed the ImmPort FLOCK modules. PC, MDN, BAH, TL, and MR provided extensive support for module development, testing, integration and hosting on the public GenePattern server at Broad Institute as well as in their module repository. RRB coordinated the BCCA development team and collaborations with the Broad Institute, JPM coordinated collaboration from the Broad Institute side and RPS from the Vaccine and Gene Therapy Institute. All authors reviewed and approved the final version of the manuscript.

## Supplementary Material

Additional file 1 **Example of a quality assessment report.** Please use any ZIP-compatible software to extract the ZIP archive file into a folder and then open the index.html file in your web browser. The interactive report shows an example of quality assessment with samples in rows and performed tests in columns. The results are color-coded with green indicating no problems, yellow indicating a warning, and red suggesting the failure of a certain test on a certain sample. The user should review flagged samples and decide whether further actions are required. Clicking on a heading shows an overview plot for a particular test. Clicking on a ‘+’ sign will expand the appropriate section, revealing detailed test results. Individual dots can be clicked on to provide the supporting analyses of tested FCM parameters underlying the final call. The example demonstrates a quality assessment report of a 96 well plate of a “Normal Donor” study performed by Becton, Dickinson and Company (BD) in order to measure immune responses to various infectious agents and cancer antigens among healthy young adults. The ≈ 8 GB of data from the mentioned study may be downloaded from http://www.ficcs.org/data/data-files/. Click here for file

Additional file 2 **GenePattern Flow Cytometry Suite Modules.** Please use any ZIP-compatible software to extract the ZIP archive file into a folder. After extraction, the folder will contain 34 ZIP files, each of these represents one of the GenePattern modules in the GenePattern Flow Cytometry Suite. If you are hosting your own GenePattern server then you can install the GenePattern Flow Cytometry Suite from these module ZIP files by following “Modules & Pipelines”, “Install from zip” at your local GenePattern site. The latest version of these modules can always be obtained by navigating to the particular module at the GenePattern public server web site ( http://genepattern.broadinstitute.org/gp/) and following the “export” link. Click here for file

Additional file 3 **Source Codes of GenePattern Flow Cytometry Suite Modules.**Please use any ZIP-compatible software to extract the ZIP archive file into a folder. There will be 35 folders after the extraction. The folder named *lib* contains the *CFCS* library that is required in order to compile the Java-based modules. In addition, the lib folder contains a ZIP compression tool that is being used by the *FCMSinglePanelQC* and *PlateQAFCS* modules to compress the results in case the server computer is a Windows-based machine. This tool is not required for Linux/Unix or Mac based servers. The additional 34 folders contain the source codes of each of the modules in the GenePattern Flow Cytometry Suite. Click here for file
