# Supplementary material for: GenePattern flow cytometry suite
Source: Source Code Biol Med. 2013 Jul 3;8:14. doi: 10.1186/1751-0473-8-14 (PMC3717030; doi:10.1186/1751-0473-8-14)
Supplement: Additional file 1 — Example of a quality assessment report. Please use any ZIP-compatible software to extract the ZIP archive file into a folder and then open the index.html file in your web browser. The interactive report shows an example of quality assessment with samples in rows and performed tests in columns. The results are color-coded with green indicating no problems, yellow indicating a warning, and red suggesting the failure of a certain test on a certain sample. The user should review flagged samples and decide whether further actions are required. Clicking on a heading shows an overview plot for a particular test. Clicking on a ‘+’ sign will expand the appropriate section, revealing detailed test results. Individual dots can be clicked on to provide the supporting analyses of tested FCM parameters underlying the final call. The example demonstrates a quality assessment report of a 96 well plate of a “Normal Donor” study performed by Becton, Dickinson and Company (BD) in order to measure immune responses to various infectious agents and cancer antigens among healthy young adults. The ≈ 8 GB of data from the mentioned study may be downloaded from http://www.ficcs.org/data/data-files/. [file 1751-0473-8-14-S1.zip › AdditionalFile1_QAReportExample/index.html]

Report


## Please wait to be redirected to your report

This page should automatically redirect to your report. If it does not, please click here to see your report.
