# Supplementary material for: GenePattern flow cytometry suite
Source: Source Code Biol Med. 2013 Jul 3;8:14. doi: 10.1186/1751-0473-8-14 (PMC3717030; doi:10.1186/1751-0473-8-14)
Supplement: Additional file 3 — Source Codes of GenePattern Flow Cytometry Suite Modules.Please use any ZIP-compatible software to extract the ZIP archive file into a folder. There will be 35 folders after the extraction. The folder named lib contains the CFCS library that is required in order to compile the Java-based modules. In addition, the lib folder contains a ZIP compression tool that is being used by the FCMSinglePanelQC and PlateQAFCS modules to compress the results in case the server computer is a Windows-based machine. This tool is not required for Linux/Unix or Mac based servers. The additional 34 folders contain the source codes of each of the modules in the GenePattern Flow Cytometry Suite. [file 1751-0473-8-14-S3.zip › AdditionalFile3_GPFCMSuiteSourceCodes/LogicleTransformFCS/edu/stanford/facs/logicle/package.html]

Logicle Data Scale

Provides an implementation of the Logicle data scale and its inverse.
The Logicle data scale is a generalization of the inverse hyperbolic sine function
or `arcsinh`.
Like `arcsinh` it is logarithmic for large data values, linear
in the neighborhood of zero, symmetric around zero and changes smoothly between
the log and linear regions.
It is more general because the width of the linear region can be independently controlled.
The scale is parameterized by the choice of a number of decades of data range to cover,
the maximum data value on scale and the width of the linear region.
It is used in flow cytometry where a large dynamic range of data, most suitable for a
logarithmic scale, is present along with other data distributed near zero
and including negative values, for which an ordinary logarithmic scale is not suitable.

## Related Documentation

The Logicle methods was originally described in the publication:
> A new "Logicle" display method avoids deceptive effects of logarithmic scaling for low signals and compensated data
>   
> David R. Parks, Mario Roederer, Wayne A. Moore,
>   
> Cytometry Part A, Volume 69A, Issue 6, Pages 541-551

available online as
PDF
or
HTML
.

The method is included in the Gating-ML proposed standard of the International Society
for the Advancement of Cytometry. The specifications for Gating-ML are available
here.

## Copyright, License and Patent

Copyright (c) 2009, The Board of Trustees of The Leland Stanford Junior University.
All rights reserved.

Redistribution and use in source and binary forms, with or without modification, are permitted provided that the following conditions are met:

- Redistributions of source code must retain the above copyright notice, this list of conditions and the following disclaimer.
- Redistributions in binary form must reproduce the above copyright notice, this list of conditions and the following disclaimer in the documentation and/or other materials provided with the distribution.
- Neither the name of the Leland Stanford Junior University nor the names of its contributors may be used to endorse or promote products derived from this software without specific prior written permission.

THIS SOFTWARE IS PROVIDED BY THE COPYRIGHT HOLDERS AND CONTRIBUTORS "AS IS" AND ANY EXPRESS OR IMPLIED WARRANTIES, INCLUDING, BUT NOT LIMITED TO, THE IMPLIED WARRANTIES OF MERCHANTABILITY AND FITNESS FOR A PARTICULAR PURPOSE ARE DISCLAIMED. IN NO EVENT SHALL THE COPYRIGHT HOLDER OR CONTRIBUTORS BE LIABLE FOR ANY DIRECT, INDIRECT, INCIDENTAL, SPECIAL, EXEMPLARY, OR CONSEQUENTIAL DAMAGES (INCLUDING, BUT NOT LIMITED TO, PROCUREMENT OF SUBSTITUTE GOODS OR SERVICES; LOSS OF USE, DATA, OR PROFITS; OR BUSINESS INTERRUPTION) HOWEVER CAUSED AND ON ANY THEORY OF LIABILITY, WHETHER IN CONTRACT, STRICT LIABILITY, OR TORT (INCLUDING NEGLIGENCE OR OTHERWISE) ARISING IN ANY WAY OUT OF THE USE OF THIS SOFTWARE, EVEN IF ADVISED OF THE POSSIBILITY OF SUCH DAMAGE.

The Logicle method is patented under United States Patent 6,954,722.
However, Stanford University does not enforce the patent for non-profit academic
purposes or for commercial use in the field of flow cytometry.

@author Wayne A. Moore
@version 1.0
