# Supplementary material for: GenePattern flow cytometry suite
Source: Source Code Biol Med. 2013 Jul 3;8:14. doi: 10.1186/1751-0473-8-14 (PMC3717030; doi:10.1186/1751-0473-8-14)
Supplement: Additional file 2 — GenePattern Flow Cytometry Suite Modules. Please use any ZIP-compatible software to extract the ZIP archive file into a folder. After extraction, the folder will contain 34 ZIP files, each of these represents one of the GenePattern modules in the GenePattern Flow Cytometry Suite. If you are hosting your own GenePattern server then you can install the GenePattern Flow Cytometry Suite from these module ZIP files by following “Modules & Pipelines”, “Install from zip” at your local GenePattern site. The latest version of these modules can always be obtained by navigating to the particular module at the GenePattern public server web site ( http://genepattern.broadinstitute.org/gp/) and following the “export” link. [file 1751-0473-8-14-S2.zip › AdditionalFile2_GPFCMSuiteModules/AddFCSEventIndex.zip!/AddFCSEventIndex.pdf]

AddFCSEventIndex Documentation

Description: Adds indexes to events in a Flow Cytometry Standard (FCS) data file.

Author: Josef Spidlen (jspidlen@bccrc.ca), gp-help@broadinstitute.org

Summary

The module takes an input FCS data file and adds an index to each event as a new parameters. The index may be used identify events in different analytical processing steps, e.g., to facilitate back-gating independently of the flow cytometry analysis tools used.

For more information about the FCS file standard and its parameters, see this page at the International Society for Advancement of Cytometry.

References

Spidlen J, Moore W, Parks D, Goldberg M, Bray C, Bierre P, Gorombey P, Hyun B, Hubbard M, Lange S, Lefebvre R, Leif R, Novo D, Ostruszka L, Treister A, Wood J, Murphy RF, Roederer M, Sudar D, Zigon R, Brinkman RR. Data file standard for flow cytometry, version FCS 3.1. *Cytometry A*. 2010;77:97-100.

Parameters

**Name**	**Description**
Input data file	An FCS data file to which you want to add indexes.
Start index	The index number for the first event in the data set; the default is 1. The index will be incremented by 1 for each of the events in the data set.
Index parameter short name	The short name ($PnN keyword) of the parameter to get the index; the default is "Index."
Index parameter long name	The long name ($PnS keyword) of the parameter to get the index; the default is blank.

Output fcs file name	The output file name. The default uses the input file name as the base.

Output Files

1. Output FCS data file

   A single FCS data file containing the events from the input data file with an additional index parameter. The index is assigned to events in the order in which they are stored in the FCS file. The first event gets the number specified in the *Start index* parameter.

Platform Dependencies

Module type: Flow Cytometry

CPU type: any

OS: any

Language: Java (1.5 minimum)

GenePattern Module Version Notes

Date	Version	Description
6/29/2012	v. 2	AddFCSEventIndex version 2 added fix for parsing empty keyword values
