# Supplementary material for: GenePattern flow cytometry suite
Source: Source Code Biol Med. 2013 Jul 3;8:14. doi: 10.1186/1751-0473-8-14 (PMC3717030; doi:10.1186/1751-0473-8-14)
Supplement: Additional file 2 — GenePattern Flow Cytometry Suite Modules. Please use any ZIP-compatible software to extract the ZIP archive file into a folder. After extraction, the folder will contain 34 ZIP files, each of these represents one of the GenePattern modules in the GenePattern Flow Cytometry Suite. If you are hosting your own GenePattern server then you can install the GenePattern Flow Cytometry Suite from these module ZIP files by following “Modules & Pipelines”, “Install from zip” at your local GenePattern site. The latest version of these modules can always be obtained by navigating to the particular module at the GenePattern public server web site ( http://genepattern.broadinstitute.org/gp/) and following the “export” link. [file 1751-0473-8-14-S2.zip › AdditionalFile2_GPFCMSuiteModules/AddFCSParameter.zip!/AddFCSParameter.pdf]

# AddFCSParameter Documentation

**Description:** Adds parameters and their values to a Flow Cytometry Standard (FCS) data file.

**Author:** Josef Spidlen (jspidlen@bccrc.ca), gp-help@broadinstitute.org

## Summary

This module inserts a calculated value for each event directly into an FCS file. Though it is best to keep the original FCS file unmodified and to keep all calculated values in external files, this module is provided for compatibility with tools that only read FCS files.

The calculated values must be in a CSV file. This can be as simple as a plain text file whose first line is the new parameter name. After that, each value should be on a separate line. There must be a calculated value for every event in the FCS file. For example, if your FCS file has 20,000 events, then your CSV file must have 20,001 rows: the first row for the parameter name and then one row for each calculated value.

FCS files typically have two names per parameter: a short name ($PnN) and a long name ($PnS). To specify both names in the header row of your CSV file, separate them with a colon, e.g., ”FL1-H:CD4” would set ($PnN) to FL1-H and ($PnS) to CD4.

A CSV file can list more than one parameter to be inserted. The CSV file is a standard file format, and each column is separated by a comma. You can add a column to your CSV file for each parameter to be inserted, still using the first line of the file for all the names of the new parameters, separated by commas. There must be a value for every event in the FCS file in every column.

For more information about the FCS file standard and its parameters, see this page at the International Society for Advancement of Cytometry.

## Usage

Maximum memory and processing time was estimated based on adding parameters from a CSV file with 1,000,000 events and 3 parameters to an FCS file with 1,000,000 events and 24 parameters stored as FCS 3.0 in the floating point data type.

- Maximum RAM: 1.9 GB
- Maximum run time: 50 seconds

## References

Spidlen J, Moore W, Parks D, Goldberg M, Bray C, Bierre P, Gorombey P, Hyun B, Hubbard M, Lange S, Lefebvre R, Leif R, Novo D, Ostruszka L, Treister A, Wood J, Murphy RF, Roederer M, Sudar D, Zigon R, Brinkman RR. Data file standard for flow cytometry, version FCS 3.1. *Cytometry A*. 2010;77:97-100.

## Parameters

| Name | Description |
|---|---|
| Input fcs data file | The input file in FCS format. |
| Input csv file | A file containing the additional parameter(s) to add to the Input FCS data file. |
| Output file name | The output file name. The default uses the input file name as the base. |

## Output Files

1. Output FCS data file

   An FCS file with all the events and parameters of the original input FCS data file, with additional parameters and their values specified in the CSV file.

## Platform Dependencies

**Module type:** Flow Cytometry

**CPU type:** any

**OS:** any

**Language:** Java (1.5 minimum)

## GenePattern Module Version Notes

| Date | Version | Description |
|---|---|---|
| 6/29/2012 | v.2 | AddFCSParameter v.2 added fix for parsing empty keyword values |
