# Supplementary material for: GenePattern flow cytometry suite
Source: Source Code Biol Med. 2013 Jul 3;8:14. doi: 10.1186/1751-0473-8-14 (PMC3717030; doi:10.1186/1751-0473-8-14)
Supplement: Additional file 2 — GenePattern Flow Cytometry Suite Modules. Please use any ZIP-compatible software to extract the ZIP archive file into a folder. After extraction, the folder will contain 34 ZIP files, each of these represents one of the GenePattern modules in the GenePattern Flow Cytometry Suite. If you are hosting your own GenePattern server then you can install the GenePattern Flow Cytometry Suite from these module ZIP files by following “Modules & Pipelines”, “Install from zip” at your local GenePattern site. The latest version of these modules can always be obtained by navigating to the particular module at the GenePattern public server web site ( http://genepattern.broadinstitute.org/gp/) and following the “export” link. [file 1751-0473-8-14-S2.zip › AdditionalFile2_GPFCMSuiteModules/AddNoiseToFCS.zip!/AddNoiseToFCS.pdf]

AddNoiseToFCS Documentation

Description: Add noise to specified parameters in an FCS data file.

Author: Josef Spidlen (jspidlen@bccrc.ca), gp-help@broadinstitute.org

Summary

The module takes an input FCS data file and adds a specified level of noise to (specified or all) parameters in file. Additionally, it can also remove saturated events, i.e., events with parameter values very close to the maximum range of particular scales. The result is saved as a new data file. This functionality serves as a useful clustering preprocessing step. Several clustering algorithms, such as FlowClust (implemented in the FlowClustClassify module) look at the variance among events in different directions. Saturated events as well as other "aligned" events may create a group (cluster) with a zero variance in a certain dimension, which prevents FlowClust from successfully finishing the clustering operation (essentially due to a division by zero exception). Using the AddNoiseToFCS module can help prevent these errors by removing saturated events and by adding a small amount of noise that is not biologically relevant; however, it minimizes the chances of having a group of perfectly aligned events. Essentially, the AddNoiseToFCS module allows you to search for a higher number of clusters compared to clustering the original FCS data file directly.

For more information about the FCS file standard and its parameters, see this page at the International Society for Advancement of Cytometry.

Usage

Maximum memory and processing time was estimated based on processing an FCS file with 1,000,000 events and 24 parameters stored as FCS 3.0 in the floating point data type.

- Maximum RAM: 2 GB
- Maximum run time: 50 seconds

References

Lo K, Brinkman RR, Gottardo R. Automated gating of flow cytometry data via robust model-based clustering. *Cytometry A*. 2008;73:321-332.

Spidlen J, Moore W, Parks D, Goldberg M, Bray C, Bierre P, Gorombey P, Hyun B, Hubbard M, Lange S, Lefebvre R, Leif R, Novo D, Ostruszka L, Treister A, Wood J, Murphy RF, Roederer M, Sudar D, Zigon R, Brinkman RR. Data file standard for flow cytometry, version FCS 3.1. *Cytometry A*. 2010;77:97-100.

Parameters

Name	Description
Input.FCS.data.file<br>(required)	The FCS data file to which the module will add noise.
Output.FCS.file.name<br>(required)	The output FCS file name. Default:<br><Input.FCS.data.file_basename>.with_noise.fcs
Parameters.to.add.noise.to<br>(optional)	List of parameters to which the module will add noise. The list may be provided as a list of indexes (e.g., "1-3,5,7") or as a list of parameter names (i.e., values of the $PnN keyword, such as "FSC-H,FL1-H,FL2-A"). By default, the noise will be added to all parameters but the Time parameter.
Noise.level<br>(required)	Noise level in percent. This is the maximum noise that will be added to an event based on the current event parameter value or on the maximum range of the appropriate parameter. The latter option will be effective if the *Uniform noise* option is selected. Default: 0.5
Noise<br>(required)	Specifies the method used to add noise. Options include:<br>• Proportional - the maximum amount of noise added to each value will be based on the value itself, e.g., low values will get lower amount of noise.<br>• Uniform - the maximum amount of noise added to each value is only dependent on the range of the particular parameter.<br>Default: Proportional

Saturated.events (required)	Whether to remove saturated events. An event is considered saturated if one or more of its parameter values are closer than 0.1% from the maximum range for that parameter (i.e., value of the appropriate $PnR keyword). Options include: Remove saturated events - saturated events will be removed from the output data set. Keep saturated events - saturated events will be kept in the output data set. Default: Remove saturated events
Random.Seed (optional)	The seed to use for the random generator. A blank field will generate a randomly selected seed. Default: 1234567

Output Files

1. Output FCS data file

   An FCS data file with the specified level of noise added to the events and saturated events removed from the data set (according to the selected options).

Platform Dependencies

**Module type:**	Flow Cytometry
**CPU type:**	any
**OS:**	any
**Language:**	Java (1.5 minimum)

GenePattern Module Version Notes

Date	Version	Description
7/3/2012	v.2	AddNoiseToFCS v.2 added fix for parsing empty keyword values, clarified description of Random Seed parameter and changed default output file name
