# Supplementary material for: GenePattern flow cytometry suite
Source: Source Code Biol Med. 2013 Jul 3;8:14. doi: 10.1186/1751-0473-8-14 (PMC3717030; doi:10.1186/1751-0473-8-14)
Supplement: Additional file 2 — GenePattern Flow Cytometry Suite Modules. Please use any ZIP-compatible software to extract the ZIP archive file into a folder. After extraction, the folder will contain 34 ZIP files, each of these represents one of the GenePattern modules in the GenePattern Flow Cytometry Suite. If you are hosting your own GenePattern server then you can install the GenePattern Flow Cytometry Suite from these module ZIP files by following “Modules & Pipelines”, “Install from zip” at your local GenePattern site. The latest version of these modules can always be obtained by navigating to the particular module at the GenePattern public server web site ( http://genepattern.broadinstitute.org/gp/) and following the “export” link. [file 1751-0473-8-14-S2.zip › AdditionalFile2_GPFCMSuiteModules/ApplyGatingML.zip!/ApplyGatingML.pdf]

GenePattern

ApplyGatingML Documentation

**Description:**	Apply a Gating-ML file on FCS data to gate and/or transform.
**Author:**	Josef Spidlen (jspidlen@bccrc.ca)
	Please see the gp-flowcyt-help Google Group (https://groups.google.com/a/broadinstitute.org/forum/#!forum/gp-flowcyt-help) for help regarding these modules. If you have a GenePattern specific question, please feel free to contact GenePattern at gp-help@broadinstitute.org

Summary

This module will apply the gates in a Gating-ML file to an FCS data file. Each gate in the Gating-ML file creates a population saved in a CSV file. The CSV file format can be used by many other modules directly or it can be transformed into FCS format by the module CsvToFcs. A summary of the gate and a plot of the gated population are also created. If the gate in the Gating-ML file is defined on compensated or transformed data, these transformations will be applied to the FCS file before the gate is applied.

For more information on the FCS file format, see the FCS 3.1 File Standard (PDF).

Usage

Maximum memory and processing time was estimated based on processing an FCS file with 1,000,000 events and 24 parameters stored as FCS 3.0 in floating point data type. Applied two two-dimensional gates, one rectangular and one elliptical.

- Maximum RAM: 1.7 GB
- Maximum run time: 3 minutes

Server Requirements

- GenePattern ≥ 3.2.0
- R 2.10.0
- flowCore 1.12.0
- flowUtils 1.6.0
- flowViz 1.10.0
- graph 1.26.0

References

- Spidlen J, Leif RC, Moore W, Roederer M, Brinkman RR, International Society for Advancement of Cytometry Data Standards Task Force. Gating-ML: Xml-based gating descriptions in flow cytometry. *Cytometry A*. 2008;73A:1151-1157.
- Spidlen J, Moore W, Parks D, Goldberg M, Bray C, Bierre P, Gorombey P, Hyun B, Hubbard M, Lange S, Lefebvre R, Leif F, Novo D, Ostruszka L, Treister A,

Wood J, Murphy RF, Roederer M, Sudar D, Zigon R, Brinkman RR. Data file standard for flow cytometry, version FCS 3.1. *Cytometry A*. 2010;77:97-100.

Links

- Wikipedia. Comma-separated values; the CSV file format description: http://en.wikipedia.org/wiki/Comma-separated_values

Parameters

Name	Description
Input FCS data file	An FCS data file to be gated.
Input Gating-ML file	A Gating-ML file specifying the gates.
Output base name	A base name for the results file names. Each gate will create a CSV file named <Output base name.gate id>.csv and a summary description <Output base name.gate id>.summary.txt. If the Output figures parameter is set to PNG then a summary plot will be made with the name <Output base name.gate id>.figure.png.<br>Default: <Input.FCS.data.file_basename>
Output figures	If set to PNG, then a summary plot will be made with the name <Output base name.gate id>.figure.png. The plot will show density plots of the gated population in each pair of dimensions.<br>Default: None, default

Input Files

1. Input FCS data file (required)

   The FCS file to be gated.

2. Input Gating-ML file (required)

   A file in Gating-ML format defining transformations and gates.

Output Files

1. <Output base name.gate id>.csv

   Each gate will create a CSV file with the name <Output base name.gate id>.csv with events in that gate. The Output base name is taken from the output base name

parameter. The gate ID is taken from the Gating-ML file. The header of the CSV file will contain parameter names and the rows will contain events that are in the gate.

2. <Output base name.gate id>.summary.txt

   Each gate produces a summary description in a text file named <Output base name.gate id>.summary.txt. This summary will include the number and percentage of events in the gate and descriptive statistics for each of the parameter, i.e., the minimum, 1st quartile, median, mean, 3rd quartile and the maximum for that parameter for events in the gate.

3. <Output base name.gate id>.figure.png

   If the Output figures parameter is set to PNG then a summary plot will be made to show density plots of the gated population in each pair of dimensions. This will be saved in the <Output base name.gate id>.figure.png file.

Example Data

Example data, including input FCS files, Gating-ML files, and expected results are included in a Gating-ML compliance test suite that may be downloaded from http://sourceforge.net/projects/flowcyt/files/Gating-ML/Gating-ML%201.5/Gating-ML.v1.5.081030.Compliance-tests.081030.zip/download

Platform Dependencies

**Module type:**	Flow Cytometry
**CPU type:**	any
**OS:**	any
**Language:**	R (minimum version 2.10)

GenePattern Module Version Notes

Version	Description
1	Initial version released 7/11/12
