# Supplementary material for: GenePattern flow cytometry suite
Source: Source Code Biol Med. 2013 Jul 3;8:14. doi: 10.1186/1751-0473-8-14 (PMC3717030; doi:10.1186/1751-0473-8-14)
Supplement: Additional file 2 — GenePattern Flow Cytometry Suite Modules. Please use any ZIP-compatible software to extract the ZIP archive file into a folder. After extraction, the folder will contain 34 ZIP files, each of these represents one of the GenePattern modules in the GenePattern Flow Cytometry Suite. If you are hosting your own GenePattern server then you can install the GenePattern Flow Cytometry Suite from these module ZIP files by following “Modules & Pipelines”, “Install from zip” at your local GenePattern site. The latest version of these modules can always be obtained by navigating to the particular module at the GenePattern public server web site ( http://genepattern.broadinstitute.org/gp/) and following the “export” link. [file 1751-0473-8-14-S2.zip › AdditionalFile2_GPFCMSuiteModules/CompensateFCS.zip!/CompensateFCS.pdf]

CompensateFCS Documentation

Description: Compensate a Flow Cytometry Standard (FCS) data file.

Author: Josef Spidlen (jspidlen@bccrc.ca), gp-help@broadinstitute.org

Please see the gp-flowcyt-help Google Group (https://groups.google.com/a/broadinstitute.org/forum/#!forum/gp-flowcyt-help) for help regarding these modules. If you have a GenePattern specific question, please feel free to contact GenePattern at gp-help@broadinstitute.org

Summary

Before analyzing your flow cytometry data, usually you need to make sure that these data are properly compensated. This may or may not be the case already. This module can help you compensate your data if it has been stored uncompensated and you have the spillover matrix. The spillover matrix may be part of your FCS already, or it may be provided in an external text file. You can use the PreviewFCS module to check whether there is a spillover matrix in your FCS file. When using the CompensateFCS module, make sure that subsequent steps in your analysis do not include compensation since you do not want to compensate multiple times.

For more information about the FCS file standard and its parameters, see the FCS 3.1 File Standard (PDF).

Usage

Maximum memory and processing time was estimated based on processing an FCS file with 1,000,000 events and 24 parameters stored as FCS 3.0 in the floating point data type.

- Maximum RAM: 1 GB
- Maximum run time: 15 seconds

References

Spidlen J, Moore W, Parks D, Goldberg M, Bray C, Bierre P, Gorombey P, Hyun B, Hubbard M, Lange S, Lefebvre R, Leif R, Novo D, Ostruszka L, Treister A, Wood J, Murphy RF, Roederer M, Sudar D, Zigon R, Brinkman RR. Data file standard for flow cytometry, version FCS 3.1. *Cytometry A*. 2010;77:97-100.

Parameters

**Name**	**Description**
FCS.data.file	The input FCS data file to be compensated.

Spillover.matrix.file	A text file with spillover matrix. Use this parameter if there is no spillover matrix in the input FCS file or to use a spillover matrix different from the matrix in the input FCS file. The text file contains the compensation in a format specified by the $SPILLOVER keyword value pair in it, either using the parameter name-based syntax as specified by the FCS 3.1 standard (e.g., /$SPILLOVER/2,FL1-H,FL2-H,1.0,0.05,0.12,1.0/) or the parameter index-based syntax as used by some vendors (e.g., /$SPILLOVER/2,3,4,1.0,0.05,0.12,1.0/).
Output.file.name	The name of the output file. The extension will match the file format specified in *Output file format*. Default: <FCS.data.file_basename>.compensated
Output.file.format	The file format to save the compensated data; choices include FCS, CSV, or RData. Default: FCS

Output Files

1. Compensated data file

   A data file with compensated values. This file may be in FCS, CSV, or Rdata format, based on the format specified. The name of the data file is specified by the Output file name parameter of the module.

2. stderr.txt

   When running the CompensateFCS module for the first time, this module connects to the Internet and downloads and installs an R / BioConductor package named flowCore. The result of the installation process will be displayed in the stderr.txt file. Note that while GenePattern will detect this as a failure of the job, the compensation will likely be correctly computed. The next time you run the CompensateFCS module, the flowCore package should already be installed and there should not be any output in the stderr.txt file.

Platform Dependencies

**Module type:**	Flow Cytometry
**CPU type:**	any
**OS:**	any
**Language:**	R 2.9.0

GenePattern

GenePattern Module Version Notes

Version	Description
1	Initial version released 7/13/12
