# Supplementary material for: GenePattern flow cytometry suite
Source: Source Code Biol Med. 2013 Jul 3;8:14. doi: 10.1186/1751-0473-8-14 (PMC3717030; doi:10.1186/1751-0473-8-14)
Supplement: Additional file 2 — GenePattern Flow Cytometry Suite Modules. Please use any ZIP-compatible software to extract the ZIP archive file into a folder. After extraction, the folder will contain 34 ZIP files, each of these represents one of the GenePattern modules in the GenePattern Flow Cytometry Suite. If you are hosting your own GenePattern server then you can install the GenePattern Flow Cytometry Suite from these module ZIP files by following “Modules & Pipelines”, “Install from zip” at your local GenePattern site. The latest version of these modules can always be obtained by navigating to the particular module at the GenePattern public server web site ( http://genepattern.broadinstitute.org/gp/) and following the “export” link. [file 1751-0473-8-14-S2.zip › AdditionalFile2_GPFCMSuiteModules/CsvToFcs.zip!/CsvToFcs.pdf]

CsvToFcs Documentation

Description: Converts Flow Cytometry data in a comma-separated values (CSV) file to a Flow Cytometry Standard (FCS) file.

Author: Josef Spidlen (jspidlen@bccrc.ca), gp-help@broadinstitute.org

Summary

This module converts Flow Cytometry data in a CSV file into an FCS data file. This may be useful if you created a CSV data file as part of your analysis and now want to analyze the data using conventional Flow Cytometry data analysis tools.

For more information about the FCS file standard and its parameters, see this page at the International Society for Advancement of Cytometry.

The CSV file has a header containing the parameter names. Parameter names can be comprised of two parts separated by a colon; e.g., "FL1-H:CD4". If two parts are detected, then these will be split into $PnN (the short name) and $PnS (the full name) in the resulting FCS file. If only a single name is detected, it will be used as the $PnN name, leaving the $PnS empty.

Range ($PnR) and data type ($DATATYPE) for the resulting FCS file can be specified. By default, this module will try to set the best settings based on the data in the file. The FCS files use the floating point data type with 32 bits if floating point numbers are detected. Otherwise, the integer data type will be used with the lowest number of bits (8, 16, 32) that allows capture of all the data. By default, the range will be set to the lowest power of 2 that is greater than the largest number in the data set.

References

Spidlen J, Moore W, Parks D, Goldberg M, Bray C, Bierre P, Gorombey P, Hyun B, Hubbard M, Lange S, Lefebvre R, Leif R, Novo D, Ostruszka L, Treister A, Wood J, Murphy RF, Roederer M, Sudar D, Zigon R, Brinkman RR. Data file standard for flow cytometry, version FCS 3.1. *Cytometry A*. 2010;77:97-100.

Parameters

**Name**	**Description**
Input.CSV.data.file	The input CSV file to be converted to FCS
Output.FCS.file.name	The output FCS file

Range	The range for the resulting data set, i.e., value of the $PnR keyword in the resulting FCS data file. The options are:<br>• **auto**: the range will be set to the lowest power of 2 greater than the largest number in the data set (default)<br>• **auto-exact**: the range will be set to (largest number in the data set) + 1<br>• **BD**: the range will be set to 262,144 (21 8) (default used by modern BD instruments)<br>• **n**: the range will be set to the explicitly-specified integer value
Data.type	The data type for the resulting data set, i.e., value of the $DATATYPE keyword in the resulting FCS data file. The options are:<br>• **auto**: the data type will be determined based on the contents of the input file, i.e., the 32-bit floating point data type will be used if floating point numbers are detected in the input CSV file; otherwise, the FCS file will use an integer data type with the lowest number of bits (8, 16, 32) that captures all the data (default)<br>• **integer**: the FCS file will use an integer data type with the lowest number of bits (8, 16, 32) that captures all the data; floating point values in the source CSV file will be rounded and saved as integers<br>• **floating point**: the FCS file will use a 32-bit floating point data type

Output Files

1. FCS data file

   An FCS 3.0 file with events from the input CSV file. The Range ($PnR) and Data type ($DATATYPE) will either be set automatically based on the data in the file or specific values may be requested using the Range and Data type parameters.

Example Data

Input: Example CSV File

Output: Example FCS File

Platform Dependencies

**Module type:**	Data Format Conversion
**CPU type:**	any
**OS:**	any
**Language:**	Java (1.6 minimum)

GenePattern Module Version Notes

Date	Version	Description
07/10/12	v.2	CsvToFcs v.2 Updated to release candidate from Josef which includes more lenient CFCS library
