# Supplementary material for: GenePattern flow cytometry suite
Source: Source Code Biol Med. 2013 Jul 3;8:14. doi: 10.1186/1751-0473-8-14 (PMC3717030; doi:10.1186/1751-0473-8-14)
Supplement: Additional file 2 — GenePattern Flow Cytometry Suite Modules. Please use any ZIP-compatible software to extract the ZIP archive file into a folder. After extraction, the folder will contain 34 ZIP files, each of these represents one of the GenePattern modules in the GenePattern Flow Cytometry Suite. If you are hosting your own GenePattern server then you can install the GenePattern Flow Cytometry Suite from these module ZIP files by following “Modules & Pipelines”, “Install from zip” at your local GenePattern site. The latest version of these modules can always be obtained by navigating to the particular module at the GenePattern public server web site ( http://genepattern.broadinstitute.org/gp/) and following the “export” link. [file 1751-0473-8-14-S2.zip › AdditionalFile2_GPFCMSuiteModules/DeIdentifyFCS.zip!/DeIdentifyFCS.pdf]

GenePattern

DeIdentifyFCS Documentation

Description: De-identify FCS data files.

Author: Josef Spidlen (jspidlen@bccrc.ca)

Please see the gp-flowcyt-help Google Group (https://groups.google.com/a/broadinstitute.org/forum/#!forum/gp-flowcyt-help) for help regarding these modules. If you have a GenePattern specific question, please feel free to contact GenePattern at gp-help@broadinstitute.org

Summary

Before sharing your FCS files outside of your organization, you might want to remove sensitive information from the file. In addition to the event data for the cells, an FCS file may contain information about the sample (source) in the text segment of the file. This module helps remove unwanted text segment information, e.g., a patient ID, while leaving the event data intact.

To see a list of keywords that are stored in the Text segment of an FCS file, run the ExtractFCSKeywords or PreviewFCS modules. Once you know the name of the keyword to remove, enter it in the Remove keywords parameter. You can enter a comma-separated list of keywords as well.

For more information on the FCS file format, see the FCS 3.1 File Standard (PDF).

Usage

Maximum memory and processing time was estimated based on processing an FCS file with 1,000,000 events and 24 parameters stored as FCS 3.0 in floating point data type.

- Maximum RAM: 10 MB
- Maximum run time: 10 seconds

Requirements

- GenePattern ≥3.2.0
- Java ≥1.6

References

Spidlen J, Moore W, Parks D, Goldberg M, Bray C, Bierre P, Gorombey P, Hyun B, Hubbard M, Lange S, Lefebvre R, Leif R, Novo D, Ostruszka L, Treister A, Wood J, Murphy RF, Roederer M, Sudar D, Zigon R, Brinkman RR. Data file standard for flow cytometry, version FCS 3.1. *Cytometry A*. 2010;77:97-100.

Parameters

Name	Description
Input FCS data file (required)	The input FCS file to be de-identified.
Remove keywords (required)	The exact name of the keyword to remove or a comma-separated list of keyword names to remove. Find the parameter names of an FCS file by running the ExtractFCSKeywords or PreviewFCS modules. If a comma is part of a keyword name that you want to remove, you can escape it by using a back slash (\).
Is.regular expression used (required)	You can also remove multiple keywords at once by specifying a regular expression. When this parameter is set to true, then the text in the *Remove.keywords* parameter is treated as a regular expression. Details on regular expression patterns can be found at http://java.sun.com/javase/6/docs/api/java/util/regex/Pattern.html<br>Default: No, default<br>Some useful examples:<br>• `(?i).*patient.*` Match the word patient anywhere in the keyword. Starting with (?i) makes the search case insensitive (e.g., matches Patient or patient or PATIENT).<br>• `Patient \d+` Matches a keyword that starts with Patient and ends with one or more digits.
Output FCS file name (required)	The output file name. Default: <Input.FCS.data.file_basename>.deidentified.<Input.FCS.data.file_extension>

Output Files

1. De-identified FCS data file

   An FCS data file with all data from the original input FCS data file but with the specified keyword/values removed. These keywords may be specified by either using a regular expression or providing a simple list of keywords. The name of the de-identified FCS data file is specified by the *Output FCS file name* parameter of the module.

2. stdout.txt

   The file stdout.txt will list the keywords that were removed from the file. It also notes if a specified keyword could not be removed (e.g., was not found) or if the regular expression does not have any match within the set of keywords in the FCS file.

Platform Dependencies

Module type: Flow Cytometry

CPU type: any

OS: any

Language: Java (minimum version 1.6)

GenePattern Module Version Notes

Version	Description
1	Initial release 7/13/12.
