# Supplementary material for: GenePattern flow cytometry suite
Source: Source Code Biol Med. 2013 Jul 3;8:14. doi: 10.1186/1751-0473-8-14 (PMC3717030; doi:10.1186/1751-0473-8-14)
Supplement: Additional file 2 — GenePattern Flow Cytometry Suite Modules. Please use any ZIP-compatible software to extract the ZIP archive file into a folder. After extraction, the folder will contain 34 ZIP files, each of these represents one of the GenePattern modules in the GenePattern Flow Cytometry Suite. If you are hosting your own GenePattern server then you can install the GenePattern Flow Cytometry Suite from these module ZIP files by following “Modules & Pipelines”, “Install from zip” at your local GenePattern site. The latest version of these modules can always be obtained by navigating to the particular module at the GenePattern public server web site ( http://genepattern.broadinstitute.org/gp/) and following the “export” link. [file 1751-0473-8-14-S2.zip › AdditionalFile2_GPFCMSuiteModules/ExtractFCSDataset.zip!/ExtractFCSDataset.pdf]

ExtractFCSDataset Documentation

Description: Extracts one or more Flow Cytometry Standard (FCS) data sets from an FCS data file.

Author: Josef Spidlen (jspidlen@bccrc.ca)

Please see the gp-flowcyt-help Google Group (https://groups.google.com/a/broadinstitute.org/forum/#!forum/gp-flowcyt-help) for help regarding these modules. If you have a GenePattern specific question, please feel free to contact GenePattern at gp-help@broadinstitute.org

Summary

Typically, there is only one data set per data file. However, in certain cases, there may be more than one data set in an FCS data file. Since most software does not support more than one data set per FCS data file, using this module is a workaround to analyze data files with multiple data sets with common tools.

This module extracts one or more data sets from a data file and store them in one or more FCS data files so that there is one data set per data file. The extracted data sets are specified by their indexes in the input file.

For more information on the FCS file format, see the FCS 3.1 File Standard (PDF).

Usage

Maximum memory and processing time was estimated based on extracting one data set from an FCS file with 1,000,000 events and 24 parameters stored as FCS 3.0 in the floating point data type. The other maximum estimate is based on another extreme case, extracting 96 data sets from an FCS data file, each data set having 8 parameters and approximately 15,000 events.

Maximum RAM: 1.7 GB/700 MB
Maximum run time: 25 seconds/15 seconds

References

Spidlen J, Moore W, Parks D, Goldberg M, Bray C, Bierre P, Gorombey P, Hyun B, Hubbard M, Lange S, Lefebvre R, Leif R, Novo D, Ostruszka L, Treister A, Wood J, Murphy RF, Roederer M, Sudar D, Zigon R, Brinkman RR. Data file standard for flow cytometry, version FCS 3.1. *Cytometry A*. 2010;77:97-100.

Parameters

Name	Description
Input data file	The input FCS data file; one or more data sets will be extracted from this file.

Output file name	The name for the output file. Uses the input data file name (for example, *name.fcs*) as the default, and adds *_datasetIndex* to the name (e.g., *name_datasetIndex.fcs*).
Dataset indexes	A list of data set indexes to be extracted from the data file. The first data set has the index 1, the second the index 2, etc.<br>The index specification may be in the form of a list, where individual index numbers are separated by commas, and intervals are specified by a separation with a hyphen. For example, the string, "1,6-9,3,12-15" will extract data set numbers 1, 3, 6, 7, 8, 9, 12, 13, 14, and 15.<br>An error will be reported if a dataset cannot be found or if it is not a list mode data set (that is, if the data set does not contain each event listed sequentially, parameter by parameter). All data sets will be extracted into separate files if dataset indexes are not specified.<br>All data sets will be extracted if no indexes are provided.

Output Files

1. FCS data files with a single list mode data set

   FCS data file(s) with only one data set per file. The data sets are extracted from the original data file. An error will be reported if a specified data set cannot be found or if it is not a list mode data set.

Platform Dependencies

**Module type:**	Flow Cytometry
**CPU type:**	any
**OS:**	any
**Language:**	Java (1.6 minimum)

GenePattern Module Version Notes

**Date**	**Version**	**Description**
07/11/12	v.2	ExtractFCSDataset v.2 Updated to include more lenient CFCS library
