# Supplementary material for: GenePattern flow cytometry suite
Source: Source Code Biol Med. 2013 Jul 3;8:14. doi: 10.1186/1751-0473-8-14 (PMC3717030; doi:10.1186/1751-0473-8-14)
Supplement: Additional file 2 — GenePattern Flow Cytometry Suite Modules. Please use any ZIP-compatible software to extract the ZIP archive file into a folder. After extraction, the folder will contain 34 ZIP files, each of these represents one of the GenePattern modules in the GenePattern Flow Cytometry Suite. If you are hosting your own GenePattern server then you can install the GenePattern Flow Cytometry Suite from these module ZIP files by following “Modules & Pipelines”, “Install from zip” at your local GenePattern site. The latest version of these modules can always be obtained by navigating to the particular module at the GenePattern public server web site ( http://genepattern.broadinstitute.org/gp/) and following the “export” link. [file 1751-0473-8-14-S2.zip › AdditionalFile2_GPFCMSuiteModules/ExtractFCSKeywords.zip!/ExtractFCSKeywords.pdf]

ExtractFCSKeywords Documentation

Description: Extracts keyword(s) value(s) from a Flow Cytometry Standard (FCS) file.

Author: Josef Spidlen (jspidlen@bccrc.ca)

Please see the gp-flowcyt-help Google Group (https://groups.google.com/a/broadinstitute.org/forum/#!forum/gp-flowcyt-help) for help regarding these modules. If you have a GenePattern specific question, please feel free to contact GenePattern at gp-help@broadinstitute.org

Summary

Specific metadata information (data about data) is usually stored in FCS data files as part of the TEXT segment in the form of keyword/value pairs. This can include information such as date and time of data acquisition, description of measured cells, comments, type and/or serial number of cytometer used, name of the operator, tube or plate identification, excitation wavelength and power for measured channels, types and voltages of detectors used, identification of the sample/specimen and its source, and/or volume of the sample run during data acquisition. This information can be lost during certain analytical steps, especially during conversion from FCS to another file format, such as CSV.

This module can be used to extract and save the information in all keyword/value pair(s) so that it can be added back later. The keywords value pairs can either be stored as a CSV file (default) or as an FCS text segment chunk in a text file.

For more information on the FCS file format, see the FCS 3.1 File Standard (PDF).

Usage

Maximum memory and processing time was estimated based on processing an FCS file with 1,000,000 events and 24 parameters stored as FCS 3.0 in the floating point data type.

- Maximum RAM: 1.2 GB
- Maximum run time: 20 seconds

References

Spidlen J, Moore W, Parks D, Goldberg M, Bray C, Bierre P, Gorombey P, Hyun B, Hubbard M, Lange S, Lefebvre R, Leif R, Novo D, Ostruszka L, Treister A, Wood J, Murphy RF, Roederer M, Sudar D, Zigon R, Brinkman RR. Data file standard for flow cytometry, version FCS 3.1. *Cytometry A*. 2010;77:97-100.

Parameters

Name	Description
Input FCS data file	The input FCS data file from which the module will extract keyword/value pairs.
Keywords output mode	The file format to be used to store the extracted keywords/value pairs. Use one of:<br>• CSV file (default): the keyword will be stored as a CSV file with two columns, *keyword* and *value*<br>• Text file with FCS text segment chunk: the keywords will be stored in a text file in the FCS text segment format; see the FCS specification for details on the FCS text segment format
Keywords output file name	The name for the output file containing the keyword/value pairs. The name of the input FCS file is the default value here. The extension will be either .csv or .txt.

Output Files

1. Extracted keyword/value pairs

   A file with the extracted keyword/value pairs in it. Depending on the *Keywords output mode* parameter, these may be saved as a CSV file with two columns (keyword and value) or as a text file containing the FCS TEXT segment chunk.

Platform Dependencies

Module type: Flow Cytometry

CPU type: any

OS: any

Language: Java (1.6 minimum)

GenePattern Module Version Notes

Date	Version	Description
7/13/2012	v.2	ExtractFCSKeywords v.2 added fix for parsing empty keyword values
