# Supplementary material for: GenePattern flow cytometry suite
Source: Source Code Biol Med. 2013 Jul 3;8:14. doi: 10.1186/1751-0473-8-14 (PMC3717030; doi:10.1186/1751-0473-8-14)
Supplement: Additional file 2 — GenePattern Flow Cytometry Suite Modules. Please use any ZIP-compatible software to extract the ZIP archive file into a folder. After extraction, the folder will contain 34 ZIP files, each of these represents one of the GenePattern modules in the GenePattern Flow Cytometry Suite. If you are hosting your own GenePattern server then you can install the GenePattern Flow Cytometry Suite from these module ZIP files by following “Modules & Pipelines”, “Install from zip” at your local GenePattern site. The latest version of these modules can always be obtained by navigating to the particular module at the GenePattern public server web site ( http://genepattern.broadinstitute.org/gp/) and following the “export” link. [file 1751-0473-8-14-S2.zip › AdditionalFile2_GPFCMSuiteModules/ExtractFCSParameters.zip!/ExtractFCSParameters.pdf]

GenePattern

ExtractFCSParameters Documentation

Description: Extracts specified parameters from a Flow Cytometry Standard (FCS) file.

Author: Josef Spidlen (jspidlen@bccrc.ca), gp-help@broadinstitute.org

Please see the gp-flowcyt-help Google Group (https://groups.google.com/a/broadinstitute.org/forum/#!forum/gp-flowcyt-help) for help regarding these modules. If you have a GenePattern specific question, please feel free to contact GenePattern at gp-help@broadinstitute.org

Summary

Sometimes, an FCS data file may contain several parameters (data dimensions) that are not relevant for a given analysis. For example, you may want to cluster your events based on cell size and granularity, so including additional fluorescence information is not relevant for this analytical step. If this is the case, you may want to remove the irrelevant parameters, which will reduce the size of the data set and may speed up subsequent analytical steps.

This module allows you to extract specified parameters from an FCS data file and save these in a new FCS data file. The subset of parameters to extract may be specified either by their indexes (e.g., 1-3, 5, 8; intervals are allowed) or as a list of parameter short names (values of the $PnN keywords, e.g., FSC-H, SSC-H, FL1-A, FL3-H). A warning will be issued if a parameter cannot be found in the source data file.

For more information on the FCS file format, see the FCS 3.1 File Standard (PDF).

Usage

Maximum memory and processing time was estimated based on extracting 75% of parameters from an FCS file with 1,000,000 events and 24 parameters stored as FCS 3.0 in the floating point data type.

- Maximum RAM: 1.4 GB
- Maximum run time: 25 seconds

References

Spidlen J, Moore W, Parks D, Goldberg M, Bray C, Bierre P, Gorombey P, Hyun B, Hubbard M, Lange S, Lefebvre R, Leif R, Novo D, Ostruszka L, Treister A, Wood J, Murphy RF, Roederer M, Sudar D, Zigon R, Brinkman RR. Data file standard for flow cytometry, version FCS 3.1. *Cytometry A*. 2010;77:97-100.

Parameters

Name	Description
Input FCS data file	The input FCS data file from which to extract parameters. Required.
Parameters to extract	A list of parameters to extract; parameters may be specified by their indexes (e.g., 1-3, 5, 8) or by their short names (e.g., FSC-H, SSC-H, FL1-A, FL3-H). Required.
Output file name	The output file name. The default uses the input file name as the base.

Output Files

1. FCS file

   An FCS data file with only the relevant parameters and all events from the original data file.

Platform Dependencies

Module type: Flow Cytometry

CPU type: any

OS: any

Language: Java (1.6 minimum)

GenePattern Module Version Notes

Version	Description
2	Fix for parsing empty keyword values
