# Supplementary material for: GenePattern flow cytometry suite
Source: Source Code Biol Med. 2013 Jul 3;8:14. doi: 10.1186/1751-0473-8-14 (PMC3717030; doi:10.1186/1751-0473-8-14)
Supplement: Additional file 2 — GenePattern Flow Cytometry Suite Modules. Please use any ZIP-compatible software to extract the ZIP archive file into a folder. After extraction, the folder will contain 34 ZIP files, each of these represents one of the GenePattern modules in the GenePattern Flow Cytometry Suite. If you are hosting your own GenePattern server then you can install the GenePattern Flow Cytometry Suite from these module ZIP files by following “Modules & Pipelines”, “Install from zip” at your local GenePattern site. The latest version of these modules can always be obtained by navigating to the particular module at the GenePattern public server web site ( http://genepattern.broadinstitute.org/gp/) and following the “export” link. [file 1751-0473-8-14-S2.zip › AdditionalFile2_GPFCMSuiteModules/FCMFeatureExtraction.zip!/FCMFeatureExtraction.pdf]

GenePattern

FCMFeatureExtraction Documentation

Description: Extraction of features from gated flow cytometry data.

Author: Josef Spidlen (jspidlen@bccrc.ca)

Please see the gp-flowcyt-help Google Group (https://groups.google.com/a/broadinstitute.org/forum/#!forum/gp-flowcyt-help) for help regarding these modules. If you have a GenePattern specific question, please feel free to contact GenePattern at gp-help@broadinstitute.org

Summary

In flow cytometry data analysis, the extraction of features of identified subpopulations is typically following after gating (or clustering) of FCS data files. These features typically include the number of events (cells) in each of the subpopulation, the proportion (percentage) of events in each subpopulation and/or the mean value of each parameter or selected parameters (e.g., the Mean Fluorescence Intensity, MFI) of each subpopulation. The FCMFeatureExtraction allows you to calculate these features from your gated/clustered data stored in several CSV files, one for each identified subpopulation.

Usage

The time and memory requirements were evaluated based on a set of 17 CSV files with clustering results of 8-parameter FCS files containing 7-parameter data and 13,359 events (360 kB of input data):

- Required RAM: 50 MB.
- Run time: 1 second

Requirements

- GenePattern ≥3.3.0
- R 2.10
- workflowCommons ≥0.1-1

References

Spidlen J, Moore W, Parks D, Goldberg M, Bray C, Bierre P, Gorombey P, Hyun B, Hubbard M, Lange S, Lefebvre R, Leif R, Novo D, Ostruszka L, Treister A, Wood J, Murphy RF, Roederer M, Sudar D, Zigon R, Brinkman RR. Data file standard for flow cytometry, version FCS 3.1. *Cytometry A*. 2010;77:97-100.

Parameters

Name	Description
Input.CSV.files<br>(required)	CSV files with clustering results (of a single data file). Each of these files is expected to include events corresponding to a single population; the events are stored in rows of the CSV files and the parameters in columns with column headings identifying flow cytometry parameters by their names. Parameter long names may be used; in this case, the parameter short name and the parameter long name are concatenated by a column (:). Event values are expected in the data portion of the CSV file.<br>The module accepts a ZIP file with all input files, or a server based directory name, or a text file listing all input files by their full server path (one input file per line)
Calculate number of cells<br>(required)	Specifies whether to calculate and output the number of cells in each subpopulation. Options are:<br>• Yes, default: Yes, include the number of cells in each subpopulation.<br>• No: No, do not include the number of cells in each subpopulation.
Calculate percentage of cells<br>(required)	Specifies whether to calculate and output the percentage (proportion) of cells in each subpopulation. Options are:<br>• Yes, default: Yes, include the percentage of cells in each sub-population.<br>• No: No, do not include the percentage of cells in each sub-population.
Calculate means<br>(required)	Calculate mean parameter values (e.g., Mean Fluorescence Intensities) for all parameters of each subpopulation? Options are:<br>• Yes, default: Yes, include the means of parameter values of cells in each subpopulation.<br>• No: No, do not include the means of parameter values of cells in each subpopulation.
Output CSV file name<br>(required)	The name of the output CSV file. Default: features.csv

Output Files

1. Extracted feature values

   A CSV file with rows corresponding to subpopulations and columns corresponding to calculated features:

   - the first column contains the name of the input file (population identification)
   - the second column contains the calculated number of cells for each of the subpopulations
   - the third column contains the calculated percentage of cells for each of the subpopulations
   - the remaining columns contain calculated mean values of all parameters for each of the sub-populations

   Some columns may not be present if the appropriate features are not selected to be calculated by the input settings of this module. Each column includes a heading suggesting the contents of the data in that column.

Platform Dependencies

Module type: Flow Cytometry

CPU type: any

OS: any

Language: R 2.10

GenePattern Module Version Notes

Date	Version	Description
07/13/12	v.1	Initial Version
