# Supplementary material for: GenePattern flow cytometry suite
Source: Source Code Biol Med. 2013 Jul 3;8:14. doi: 10.1186/1751-0473-8-14 (PMC3717030; doi:10.1186/1751-0473-8-14)
Supplement: Additional file 2 — GenePattern Flow Cytometry Suite Modules. Please use any ZIP-compatible software to extract the ZIP archive file into a folder. After extraction, the folder will contain 34 ZIP files, each of these represents one of the GenePattern modules in the GenePattern Flow Cytometry Suite. If you are hosting your own GenePattern server then you can install the GenePattern Flow Cytometry Suite from these module ZIP files by following “Modules & Pipelines”, “Install from zip” at your local GenePattern site. The latest version of these modules can always be obtained by navigating to the particular module at the GenePattern public server web site ( http://genepattern.broadinstitute.org/gp/) and following the “export” link. [file 1751-0473-8-14-S2.zip › AdditionalFile2_GPFCMSuiteModules/FCMSinglePanelQC.zip!/FCMSinglePanelQC.pdf]

FCMSinglePanelQC Documentation

Description: Perform quality control of a single panel from cytometry data.

Author: Josef Spidlen (jspidlen@bccrc.ca)

Please see the gp-flowcyt-help Google Group (https://groups.google.com/a/broadinstitute.org/forum/#!forum/gp-flowcyt-help) for help regarding these modules. If you have a GenePattern specific question, please feel free to contact GenePattern at gp-help@broadinstitute.org

Summary

Before analyzing your Flow Cytometry Standard (FCS) data files, you may want to perform quality control (QC) tests of your data. Quality control is an important step in the data analysis pipeline, often helping researchers identify differences in samples originating from changes in conditions that are probably not biologically motivated. The general aim is to establish a QC criterion to give special consideration to these samples or even exclude them from further analysis.

This module is based on functionality implemented in R/Bioconductor in the flowQ package. There are four tests available for the analysis of single-panel experiments:

- the cell number control test
- the boundary events test
- the timeflow test
- the timeline test

These tests may be individually switched on or off. For QC of multipanel experiments, see the PlateQAFCS module.

Usage

The time and memory requirements were evaluated based on the following tests:

- A set of 70 FCS files containing 7 data parameters and 12,000-106,000 events each (28 MB of input data): Required RAM: 900 MB. Run time: 8 minutes
- A set of 102 FCS files containing 7 data parameters and 10,000-38,000 events each (23 MB of input data): Required RAM: 1 GB. Run time: 9 minutes
- A set of 48 FCS files containing 13 data parameters and 22,000-70,000 events each (142 MB of input data): Required RAM: 2.2 GB. Run time: 20 minutes
- A set of 48 FCS files containing 13 data parameters and 90,000-120,000 events each (250 MB of input data): Required RAM: 3.3 GB. Run time: 35 minutes

References

Gentleman R, Hahne F, Kettman JR, Le Meur N, Gopalakrishnan N. R/bioconductor package flowQ: Quality control for flow cytometry. http://bioconductor.org/packages/release/bioc/html/flowQ.html

Spidlen J, Moore W, Parks D, Goldberg M, Bray C, Bierre P, Gorombey P, Hyun B, Hubbard M, Lange S, Lefebvre R, Leif R, Novo D, Ostruszka L, Treister A, Wood J, Murphy RF, Roederer M, Sudar D, Zigon R, Brinkman RR. Data file standard for flow cytometry, version FCS 3.1. *Cytometry A*. 2010;77:97-100.

Parameters

**Name**	**Description**
Input.FCS.data.files	The input FCS files to be tested. The module accepts a ZIP file with all input files, or a server based directory name, or a text file listing all input files by their full server path (one input file per line)
Perform.cell.number.test	Perform the most simple QC test: check whether the total number of events for a given sample is above a certain threshold. This threshold will be automatically determined from the average number of events per sample. Default: Yes
Perform.boundary.events.test	Perform the boundary events test. A typical flow cytometer has a certain dynamic range over which it acquires a signal. All fluorescence intensities that fall out of this range will be accumulated as margin events, i.e., they are artificially given the minimum or the maximum value of the dynamic range. A high number of these events usually indicates potential problems with compensation, instrument settings, or drifts. Default: Yes
Perform.timeflow.test	Perform the timeflow test. Most flow cytometers record a time tick for each event they measure. Based on this parameter, this test can detect check whether the flow rate was steady and uninterrupted. Default: Yes
Perform.timeline.test	Perform the timeline test, i.e., analyze each of the fluorescence intensities separately as a function of time and flag samples with unexpected non-random behavior. Default: Yes
Zip.output	Where the ZIP the output report to a single ZIP file. Default: Yes

Output Files

1. Quality control report

   An HTML report summarizing the quality control tests. Depending on the Zip.output parameter, all the report files may be compressed into a single ZIP file named FCMSinglePanelQCReport.zip, or they will be left as separate files. Please note that the report typically consists of hundreds of output files unless they are zipped together. A single ZIP may be easier to download; however, if files are not zipped together then the report may be viewed online on the server by opening the FCMSinglePanelQCReport/report.html file. The report displays an overview table with rows corresponding to tested samples and rows to selected quality control processes. Clicking on the heading will show an appropriate overview plot for that process. Clicking on a + sign will expand the appropriate information. The colors in this report specify the following results:

   - green – test passed
   - yellow – there is a warning associated with the test
   - red – test failed

Platform Dependencies

**Module type:**	Flow Cytometry
**CPU type:**	any
**OS:**	any
**Language:**	R (2.9.0)

GenePattern Module Version Notes

Version	Description
1	Initial version released 7/13/12
