# Supplementary material for: GenePattern flow cytometry suite
Source: Source Code Biol Med. 2013 Jul 3;8:14. doi: 10.1186/1751-0473-8-14 (PMC3717030; doi:10.1186/1751-0473-8-14)
Supplement: Additional file 2 — GenePattern Flow Cytometry Suite Modules. Please use any ZIP-compatible software to extract the ZIP archive file into a folder. After extraction, the folder will contain 34 ZIP files, each of these represents one of the GenePattern modules in the GenePattern Flow Cytometry Suite. If you are hosting your own GenePattern server then you can install the GenePattern Flow Cytometry Suite from these module ZIP files by following “Modules & Pipelines”, “Install from zip” at your local GenePattern site. The latest version of these modules can always be obtained by navigating to the particular module at the GenePattern public server web site ( http://genepattern.broadinstitute.org/gp/) and following the “export” link. [file 1751-0473-8-14-S2.zip › AdditionalFile2_GPFCMSuiteModules/FCSNormalization.zip!/FCSNormalization.pdf]

FCSNormalization Documentation

Description: Perform per-channel normalization of Flow Cytometry Standard (FCS) data files.

Author: Josef Spidlen (jspidlen@bccrc.ca)

Please see the gp-flowcyt-help Google Group (https://groups.google.com/a/broadinstitute.org/forum/#!forum/gp-flowcyt-help) for help regarding these modules. If you have a GenePattern specific question, please feel free to contact GenePattern at gp-help@broadinstitute.org

Summary

Between-sample variation in high throughput flow cytometry data poses a significant challenge for analysis of large scale data sets, such as those derived from multi-center clinical trials. It is often hard to match biologically relevant cell populations across samples due to technical variation in sample acquisition and instrumentation differences. Thus normalization of data is a critical step prior to analysis, particularly in large-scale data sets from clinical trials, where group specific differences may be subtle and patient-to-patient variation common.

This module implements a normalization method that remove technical between-sample variation by aligning prominent features (landmarks) in the raw data on a per-channel basis. For more details on the normalization method, see Hahne et al (2010).

Usage

Maximum memory and processing time was estimated based on processing an FCS file with 1,000,000 events and 24 parameters stored as FCS 3.0 in the floating point data type.

- Maximum RAM: 1 GB
- Maximum run time: 15 seconds

References

Hahne F, Khodobakhshi AH, Bashashati A, Wong CJ, Gascoyne RD, Weng AP, Seyfert-Margolis V, Bourcier K, Asare A, Lumley T, Gentleman R, Brinkman RR. Per-channel basis normalization methods for flow cytometry data. *Cytometry A*. 2010;77:121-131.

Spidlen J, Moore W, Parks D, Goldberg M, Bray C, Bierre P, Gorombey P, Hyun B, Hubbard M, Lange S, Lefebvre R, Leif R, Novo D, Ostruszka L, Treister A, Wood J, Murphy RF, Roederer M, Sudar D, Zigon R, Brinkman RR. Data file standard for flow cytometry, version FCS 3.1. *Cytometry A*. 2010;77:97-100.

Parameters

Name	Description
Input.FCS.files	The input FCS files to be normalized.
Channels.to. normalize	Comma-separated list of parameter short names to normalize.
Apply.ASinH	Apply the ASinH transformation prior to normalization. Default: Yes
Create.report	Create an additional histogram channel report. Default: Yes
Landmarks. number	The number of landmarks to be used for normalization. Default: 1
Zip.output	Whether to ZIP the normalized FCS files to a single ZIP file. Default: Yes

Output Files

1. Normalized data

   Normalized FCS data files will be saved in a single zip file (named NormalizedData.zip if the Zip.output parameter is set to TRUE) or they will be saved in a .normalized.data directory with an HTML index file (named normalized.data.html) listing these normalized FCS files so that they can be downloaded individually (if the Zip.output parameter is set to FALSE). Besides normalization, the data will also be ASinH-transformed if this transformation has been enabled by the user.

2. Histogram channel reports

   If specified in the Create.report parameter, additional histogram channel reports will be created for each of the channels selected for normalization in the Channels.to.normalize parameter. These will be saved in the PDF format in a file named original.vs.normalized.channel name.pdf.

Platform Dependencies

Module type: Flow Cytometry

CPU type: any

OS: any

Language: R 2.9.0

GenePattern

GenePattern Module Version Notes

Date	Version	Description
07/13/12	v.1	Initial Version
