# Supplementary material for: GenePattern flow cytometry suite
Source: Source Code Biol Med. 2013 Jul 3;8:14. doi: 10.1186/1751-0473-8-14 (PMC3717030; doi:10.1186/1751-0473-8-14)
Supplement: Additional file 2 — GenePattern Flow Cytometry Suite Modules. Please use any ZIP-compatible software to extract the ZIP archive file into a folder. After extraction, the folder will contain 34 ZIP files, each of these represents one of the GenePattern modules in the GenePattern Flow Cytometry Suite. If you are hosting your own GenePattern server then you can install the GenePattern Flow Cytometry Suite from these module ZIP files by following “Modules & Pipelines”, “Install from zip” at your local GenePattern site. The latest version of these modules can always be obtained by navigating to the particular module at the GenePattern public server web site ( http://genepattern.broadinstitute.org/gp/) and following the “export” link. [file 1751-0473-8-14-S2.zip › AdditionalFile2_GPFCMSuiteModules/FcsToCsv.zip!/FcsToCsv.pdf]

GenePattern

FcsToCsv Documentation

Description: Converts a Flow Cytometry Standard (FCS) file to a comma-separated values (CSV) file

Author: Josef Spidlen (jspidlen@bccrc.ca), gp-help@broadinstitute.org

Summary

Various software tools and packages (e.g., R/BioConductor) implement useful analytical functionality that is generally applicable on data from biological and other domains. Though these tools may not support the Flow Cytometry data file standards, they may be useful in the analysis of Flow Cytometry data.

Thus, this module can be used to convert an FCS data file to a CSV file. Note that the CSV file will contain only the data component of the FCS data file. Specifically, the CSV file will contain a table with columns corresponding to FCS parameters (headers identifying parameters by names, i.e., either values of the $PnN keyword or the $PnN and $PnS keywords) and rows corresponding to events in the FCS file.

In CSV files, there is no place to keep additional metadata that may be present in the FCS text segment as keyword/value pairs. If you want to keep these, this module can extract and store them in an additional file that you can merge back into your analysis subsequently. Alternatively, the ExtractFCSKeywords module can be used for that purpose.

Unless otherwise specified, values in the FCS file will be converted from channel values (commonly on a log scale for fluorescence parameters) to linear scale values. A regular precision (32-bit floating point) is used for this conversion by default, double precision may be requested.

For more information about the FCS file standard and its parameters, see this page at the International Society for Advancement of Cytometry.

References

Spidlen J, Moore W, Parks D, Goldberg M, Bray C, Bierre P, Gorombey P, Hyun B, Hubbard M, Lange S, Lefebvre R, Leif R, Novo D, Ostruszka L, Treister A, Wood J, Murphy RF, Roederer M, Sudar D, Zigon R, Brinkman RR. Data file standard for flow cytometry, version FCS 3.1. *Cytometry A*. 2010;77:97-100.

Parameters

Name	Description
Input.FCS.data.file	The input FCS file to be converted to CSV
Output.CSV.file.name	The name of the resulting output CSV file

Use.full.names	Specify whether to try to extract full parameter names from the FCS file:<br>• true=use the combination of short and long names ($PnN and $PnS values) for the parameter names in the CSV file (default)<br>• false=use short names only ($PnN) for parameter names in the CSV file
Output.keywords.file.name	The file name to store keyword value pairs extracted from the FCS file (uses the input file name as the default base)
Output.keywords.mode	Specify the file format for storing keywords extracted from the FCS file:<br>• CSV=store as a CSV file (default)<br>• FCS=store keywords in the FCS text segment format<br>• None=do not extract the keyword/value pairs
Channel.to.scale.conversion	Specify whether to convert log channel values to linear scale or not:<br>• true=convert channel to linear scale (default)<br>• no=no conversion, store same as in the FCS file
Precision	Specify whether to use regular (32-bit, default) or double (64-bit) precision:<br>• false=use regular precision (default); data will be stored using ASCII representation of 32-bit floating point numbers<br>• true=use double precision; data will be stored using ASCII representation of 64-bit floating point numbers<br>32-bit precision is sufficient for most applications. The size of the data file using the double precision will be approximately twice that of a file using regular precision.

GenePattern

Output Files

1. CSV data file: A CSV file with events from the input FCS file
2. Extracted keyword value pairs: Optionally, a file with extracted keyword/value pairs will be provided. The format of these keywords and whether the file will be provided is determined by the option selection for the *Output keywords mode* parameter. The keyword/value pairs may be stored either as a CSV file with two columns (*keyword* and *value*), or as a FCS text segment, e.g., /$CYT/FACSCalibur/CYTNUM/E3820/...

Example Data

Input: Example FCS File

Output: Example CSV File

Platform Dependencies

**Module type:**	Data Format Conversion
**CPU type:**	any
**OS:**	any
**Language:**	Java (1.5 minimum)

Version Notes

GenePattern Module Version Notes

Date	Version	Description
7/2/2012	v.3	FcsToCsv v.3 added fix for parsing empty keyword values
