# Supplementary material for: GenePattern flow cytometry suite
Source: Source Code Biol Med. 2013 Jul 3;8:14. doi: 10.1186/1751-0473-8-14 (PMC3717030; doi:10.1186/1751-0473-8-14)
Supplement: Additional file 2 — GenePattern Flow Cytometry Suite Modules. Please use any ZIP-compatible software to extract the ZIP archive file into a folder. After extraction, the folder will contain 34 ZIP files, each of these represents one of the GenePattern modules in the GenePattern Flow Cytometry Suite. If you are hosting your own GenePattern server then you can install the GenePattern Flow Cytometry Suite from these module ZIP files by following “Modules & Pipelines”, “Install from zip” at your local GenePattern site. The latest version of these modules can always be obtained by navigating to the particular module at the GenePattern public server web site ( http://genepattern.broadinstitute.org/gp/) and following the “export” link. [file 1751-0473-8-14-S2.zip › AdditionalFile2_GPFCMSuiteModules/FlowClustClassifyFCS.zip!/FlowClustClassifyFCS.pdf]

FlowClustClassifyFCS Documentation

Description: Finding populations in an FCS data file using the FlowClust algorithm.

Author: Josef Spidlen, jspidlen@bccrc.ca, Aaron Barsky

Please see the gp-flowcyt-help Google Group (https://groups.google.com/a/broadinstitute.org/forum/#!forum/gp-flowcyt-help) for help regarding these modules. If you have a GenePattern specific question, please feel free to contact GenePattern at gp-help@broadinstitute.org

Summary

The FlowClustClassifyFCS module automatically assigns cells to populations by clustering FCS data. Internally, this module is using functionality implemented in the flowClust package in R/BioConductor. This algorithm requires a parameter, K, which specifies how many populations the algorithm shall be looking for. If you do not know in advance how many populations you expect to find, you can use the SuggestNumberOfPopulationsFCS module to estimate a K value.

Usage

Maximum memory and processing time was estimated based on processing an FCS file with 1,000,000 events and 21 parameters; the search was performed to find 3 clusters using an Intel(R) Core(TM)2 Extreme CPU X9650 @ 3.00GHz.

- Maximum RAM: 2.3 GB
- Maximum run time: 1 hour 15 minutes

References

Kenneth Lo, Ryan R. Brinkman, and Raphael Gottardo. Automated gating of flow cytometry data via robust model-based clustering. Cytometry A, 73:321–332, February 2008.

Josef Spidlen, Wayne Moore, David Parks, Michael Goldberg, Chris Bray, Pierre Bierre, Peter Gorombey, Bill Hyun, Mark Hubbard, Simon Lange, Ray Lefebvre, Robert R. Leif, David Novo, Leo Ostruszka, Adam Treister, James Wood, Robert F. Murphy, Mario Roederer, Damir Sudar, Robert Zigon, and Ryan R. Brinkman. Data file standard for flow cytometry, version fcs 3.1. Cytometry A, 77(1):97–100, 2010.

GenePattern. The cls file format, accessed November 2009. http://www.broadinstitute.org/cancer/software/genepattern/tutorial/gp_fileformats.html

Parameters

Name	Description
K*	The number of populations that the algorithm shall be looking for. If you do not know the K value, you can use the SuggestNumberOfPopulationsFCS module to estimate it.

CSV.file.name	The ”base” name of the output population files in the CSV file format. Each population file will have the name CSV.file.name-n.csv, where n is a number from 1 to K.<br>Default: <Input.FCS.data.file_basename>
CLS.file.name	The name of the CLS 1 output file, which will store the population number for each event.<br>Default: <Input.FCS.data.file_basename>.cls
Quantile	Outlier quantile. Events outside the specified quantile region will be classified as outliers and be assigned to class 0.<br>Default: 0.9
Channels	A comma-separated list of parameter short names ($PnN values) that shall be used to classify the populations. All parameters will be used by default, i.e., if no value for the Channels parameter is provided. The Time parameter is excluded automatically.

Input Files

1. Input.FCS.data.file

   The FCS data file to be clustered using the FlowClust algorithm. Events (cells) from this data file will be separated into K subpopulations based on specified measured characteristics (channels).

Output Files

1. CSV.file.name-n.csv

   The module outputs K CSV files containing the cells in each of the populations. The columns of the CSV file correspond to the parameters of the FCS file and the column headings will be created based on the short and long parameter names ($PnN and $PnS keyword values) as a single name separated by ;, i.e., $PnN:$PnS, for example: FL2-H:CD69 PE. The file names will be constructed as CSV.file.name-n.csv, where n is a number from 1 to K, and CSV.file.name is the value of the CSV.file.name parameter.

2. CLS.file.name.cls

   A classification results file in the CLS format, which will store the population number for each event in a single file. The order of the events is the same as in the original FCS file. The population numbers are from 1 to K. In addition, a population number 0 means that FlowClust did not assign the event to any population (i.e., the event is an outlier).

Example Data

Example.Input.FCS.data.file.fcs is included in the module source codes; it can be run with K=4 and the rest of the parameters left to their defaults.

Platform Dependencies

**Module type:**	Flow Cytometry
**CPU type:**	Any
**OS:**	Any
**Language:**	R (2.10)

GenePattern Module Version Notes

Date	Version	Description
07/13/12	v.1	Initial Version
