# Supplementary material for: GenePattern flow cytometry suite
Source: Source Code Biol Med. 2013 Jul 3;8:14. doi: 10.1186/1751-0473-8-14 (PMC3717030; doi:10.1186/1751-0473-8-14)
Supplement: Additional file 2 — GenePattern Flow Cytometry Suite Modules. Please use any ZIP-compatible software to extract the ZIP archive file into a folder. After extraction, the folder will contain 34 ZIP files, each of these represents one of the GenePattern modules in the GenePattern Flow Cytometry Suite. If you are hosting your own GenePattern server then you can install the GenePattern Flow Cytometry Suite from these module ZIP files by following “Modules & Pipelines”, “Install from zip” at your local GenePattern site. The latest version of these modules can always be obtained by navigating to the particular module at the GenePattern public server web site ( http://genepattern.broadinstitute.org/gp/) and following the “export” link. [file 1751-0473-8-14-S2.zip › AdditionalFile2_GPFCMSuiteModules/FlowFingerprinting.zip!/FlowFingerprinting.pdf]

GenePattern

FlowFingerprinting Documentation

Description: Fingerprint a flowset.

Author: Karin Breuer (kbreuer@bccrc.ca)

Please see the gp-flowcyt-help Google Group (https://groups.google.com/a/broadinstitute.org/forum/#!forum/gp-flowcyt-help) for help regarding these modules. If you have a GenePattern specific question, please feel free to contact GenePattern at gp-help@broadinstitute.org

Summary

The FlowFingerprinting module takes a flow set and generates a description of the multivariate probability distribution function of its flow cytometry data in the form of a fingerprint. As such, it is independent of a presumptive functional form for the distribution, in contrast with model-based methods such as Gaussian Mixture Modeling. Internally, this module uses the FlowFP package from R/BioConductor. FlowFingerprinting can be used for QC screening.

For more information on the FCS file format, see the FCS 3.1 File Standard (PDF).

Usage

Maximum memory and processing time was estimated based on processing 96 experiments with 7 parameters, using an Intel® Core2 Extreme CPU X9650 at 3.00GHz.

- Maximum RAM: 5.6 GB
- Maximum run time: 60 seconds

Requirements

- GenePattern ≥3.2.1
- R =2.10
- flowCore ≥1.12.0
- flowViz ≥1.12.0
- flowFP ≥1.4.0

References

Gentleman R, Carey V, Bates D, Bolstad B, Dettling M, Dudoit S, Ellis B, Gautier L, Ge Y, Gentry J, Hornik K, Hothorn T, Huber W, Iacus S, Irizarry R, Leisch F, Li C, Maechler M, Rossini A, Sawitzki G, Smith C, Smyth G, Tierney L, Yang J, Zhang J. Bioconductor: open software development for computational biology and bioinformatics. *Genome Biology*. 2004;5(10):R80.

Holyst H, Rogers W. R/bioconductor package flowfp: Fingerprint generation of flow cytometry data, used to facilitate the application of machine learning and datamining tools for flow cytometry. Accessed April 2010. http://bioconductor.org/packages/release/bioc/html/flowFP.html.

Rogers WT, Holyst HA. Flowfp: a bioconductor package for fingerprinting flow cytometric data. *Adv Bioinformatics*. 2009:193947.

Parameters

**Name**	**Description**
Input FCS data file (required)	The FCS file(s) to be fingerprinted.
Model FCS. data file	Training data (one or more FCS files) for the underlying model. If none, the input FCS data file is used for creating a default model.
Model parameters	A comma-separated list of dimensions/parameters/channels used for the underlying model. The module accepts both: a list of parameter names (e.g., FSC-H, SSC-H, FL1-H, FL4-H) as well as a list of parameter indexes (e.g., 1,2,4,5,8).
Model number of recursions	Number of times the FCS training data will be subdivided.
Model dequantize	Option to make all events in the training set unique. Default: Yes
Model sample size	Used to specify the per-flowFrame sample size of the data to use in model generation. If NULL, all of the data is used. Setting this to a smaller number will speed up processing, at the cost of accuracy.
Output QC screening fingerprint stack	If yes, a plot of type ”stack” is generated. Default: Yes
Output QC screening gate consistency	If yes, a plot of type ”qc” is generated. Default: Yes

Output QC screening plate	If yes, a plot of type ”plate” is generated. Default: No
Output csv file	If a name is given, a CSV file is generated containing one feature vector per row. Default name: fingerprints.csv
Output file transformation	Transformation used on data for the CSV output file. ”raw” is the number of events in each bin, ”normalized” is the ratio of the number of events divided by the expected number (the expected number of events is calculated by dividing the total number of events in the instance used to make the fingerprint by the number of features in the fingerprint). ”log2norm” is the log2 of the normalized values. Default: raw
Workflow parameter file	A CSV file containing parameters if the module is used in a pipeline.

Output Files

1. Stack plot

   A PNG image showing a stack plot.
2. Gates QC plot

   A PNG image showing a quality control (QC) plot.
3. Plate plot

   A PNG image showing a plate plot.
4. CSV file

   A CSV file, containing one feature vector per row. The columns contain the number of events per bin.

Platform Dependencies

**Module type:**	Flow Cytometry
**CPU type:**	any
**OS:**	any
**Language:**	R (2.10)

GenePattern Module Version Notes

Version	Description
1	Initial release 7/13/12.
