# Supplementary material for: GenePattern flow cytometry suite
Source: Source Code Biol Med. 2013 Jul 3;8:14. doi: 10.1186/1751-0473-8-14 (PMC3717030; doi:10.1186/1751-0473-8-14)
Supplement: Additional file 2 — GenePattern Flow Cytometry Suite Modules. Please use any ZIP-compatible software to extract the ZIP archive file into a folder. After extraction, the folder will contain 34 ZIP files, each of these represents one of the GenePattern modules in the GenePattern Flow Cytometry Suite. If you are hosting your own GenePattern server then you can install the GenePattern Flow Cytometry Suite from these module ZIP files by following “Modules & Pipelines”, “Install from zip” at your local GenePattern site. The latest version of these modules can always be obtained by navigating to the particular module at the GenePattern public server web site ( http://genepattern.broadinstitute.org/gp/) and following the “export” link. [file 1751-0473-8-14-S2.zip › AdditionalFile2_GPFCMSuiteModules/FlowMeansCluster.zip!/FlowMeansCluster.pdf]

FlowMeansCluster Documentation

Description: Clustering of flow cytometry data using the FlowMeans algorithm.

Author: Nima Aghaeepour (naghaeep@bccrc.ca), Josef Spidlen (jspidlen@bccrc.ca)

Please see the gp-flowcyt-help Google Group (https://groups.google.com/a/broadinstitute.org/forum/#!forum/gp-flowcyt-help) for help regarding these modules. If you have a GenePattern specific question, please feel free to contact GenePattern at gp-help@broadinstitute.org

Summary

This module uses the FlowMeans clustering approach to perform automated gating of cell populations in flow cytometry data. This algorithm applies a non-parametric approach to perform automated gating of cell populations in flow cytometry data. Clustering results are obtained with counting the number of modes in every single dimension followed by multidimensional clustering. Then adjacent clusters (in terms of Euclidean or Mahalanobis distance) are merged. The number of clusters is determined using change point detection algorithm based on piecewise linear regression. This approach allows multiple clusters to represent the same population. It enables the algorithm to find non-spherical cell populations.

Usage

Maximum memory and processing time was estimated based on clustering 11 dimensions from an FCS file with 21 dimensions and 1,000,000 events. For this purpose, the clustering job uses about 1.2 GB or RAM with the processing time varying from a few minutes up to a few hours depending on parameter settings. When clustering such a large file, the most time consuming part is drawing the results and therefore, switching off the graphical output will significantly speed up the job. Changing the distance matrix update to averaging rather than using the default Mahalanobis distance will speed up the job as well.

- Clustering with maximum number of clusters set to 15, disabled drawing plots, updating distance using averaging, one initialization set and default values for additional parameters: run time of 12 minutes.
- Clustering with maximum number of clusters set to 30, disabled drawing plots, updating distance using averaging and default values for additional parameters: run time of 24 minutes.
- Clustering with maximum number of clusters set to 30, disabled drawing plots and default values for additional parameters: run time of 50 minutes.
- Clustering with maximum number of clusters set to 30, enabled drawing plots and updating using averaging: run time 2 hours.
- Clustering with maximum number of clusters set to 30, enabled drawing plots and default values for additional parameters: run time 2 hours and 30 minutes.

Requirements

- GenePattern 3.2.1
- R 2.11
- flowCore 1.13.9
- flowMeans 0.99.4
- workflowCommons 0.1-1

References

Aghaeepour N, Nikolic R, Hoos HH, Brinkman RR. Rapid cell population identification in flow cytometry data. Cytometry A. 2011; 79(1):6-13.

Aghaeepour N. R/bioconductor package flowmeans: Non-parametric flow cytometry data gating, accessed April 2010. http://www.bioconductor.org/packages/devel/bioc/html/flowMeans.html

Parks DR, Roederer M, Moore WA. A new "logicle" display method avoids deceptive effects of logarithmic scaling for low signals and compensated data. *Cytometry A*. 2006; 69:541-551.

Parameters

Name	Description
Input.FCS.data.file (required)	The FCS file to be clustered, i.e., events/cells automatically separated into subpopulations.
Dimensions	A comma-separated list of dimensions (flow cytometry parameters/channels) to be used for clustering. The module accepts both, a list of parameter names (e.g., FSC-H, SSC-H, FL1-H, FL4-H) as well as a list of parameter indexes (e.g., 1,2,4,5,8). All dimensions but Time will be used if the Dimensions parameter is not provided.
Transformation (required)	Specifies the transformation to apply prior clustering. Fluorescence channels are usually better visualized and clustered using a transformation. However, note that applying a transformation where a high curvature region of the transformation coincides with regions of non-near-zero density of events can also generate spurious populations.<br>Options are:<br>• ASinH (Hyperbolic Arcus Sine): The ASinH transformation produces good results on most data. (default)

	• Logarithmic transformation: The logarithmic transformation can be used if not too much data (or no data of interest) is located around the axes.<br>• Logicle transformation: Logicle transformation is an alternative to logarithmic transformation that better handles data around the axes.<br>• No transformation: The data will be used as stored in the FCS data file.
Dimensions.to.transform	A comma-separated list of dimensions (channels) to be transformed as specified by previous parameter. This will be ignored if no transformation is specified. If this parameter is not provided and transformation is specified above, the algorithm will use heuristics to identify parameters that shall be transformed. These heuristics are based on how parameters are stored in the FCS file, their resolution and their name. Again, you can use either parameter names or parameter indexes to specify dimensions to transform.
Maximum.number.of.clusters	The maximum number of clusters that the module will try. This value will be estimated automatically if the value of this parameter is not provided.
Number.of.clusters	A fixed specified number of clusters for which the module will search. This parameter cannot be combined with the Maximum number of clusters parameter. The number of clusters will be estimated automatically if this parameter is not provided. This will be a number from the range of 1 to *Maximum.number.of.clusters*, where the maximum number of clusters can also be estimated automatically if not explicitly provided.
Maximum.number.of.iterations (required)	The maximum number of iterations allowed within the K-means component of the clustering. The default value is usually sufficient. (Default: 50)
Number.of.initialization.sets (required)	The number of random sets used for initialization of the K-means component of the clustering. The default value is usually sufficient. (Default: 10)
Distance.used (required)	The distance metric being used within the FlowMeans algorithm. You can use one of the following:

	• Mahalanobis distance, default (highly recommended)<br>• Euclidean distance
Standardize.<br>data<br>(required)	Specifies whether each data dimension will be standardized (i.e., transformed to a [0,1] interval) before clustering. This usually does no harm and is important if data in dimensions used for clustering covers significantly different ranges for different dimensions. Options are:<br>• Yes, standardize data before clustering, default: Data will be standardized.<br>• No, keep data as is: Data will not be standardized.
Distance.<br>matrix.update<br>(required)	How to update the distance matrix at each merging iteration. Options are:<br>• Update using Mahalanobis distance, default: The Mahalanobis distance will be used to update the distance matrix. Note that this option is impossible if Euclidean distance is set in the *Distance.used* parameter.<br>• Update using averaging: Averaging will be used to update the distance matrix.<br>• Do not update at all: The distance matrix will not be updated at merging iterations.
Transform.<br>residuals<br>(required)	Specifies whether or not to transform residuals to orthogonal distance. Options are:<br>• Yes, transform residuals to orthogonal distance: Residuals will be transformed.<br>• No, do not transform residuals, default: Residuals will not be transformed.
Max.points.for.<br>covariance	The maximum number of data points (events) used for calculating the covariance. All data points will be used if this value is not provided. This is usually a safe option; however, using all events may not always be necessary and limiting this number may slightly speed up the clustering.
Max.points.for.<br>kernel.density	The maximum number of data points (events) used for counting the modes using kernel density estimation. All data points will be used if this value is not provided. This is usually a safe option; however, using all events may not always be necessary and

	limiting this number may slightly speed up the clustering.
Add.noise (required)	Specify whether a little bit of uniform noise be added to the data to prevent singularity issues. Options are:<br>• Yes, add a bit of noise, default: Noise will be added.<br>• No, do not add any noise: Noise will not be added.
Draw.plots (required)	Specify whether plots of clustered data will be created for quick visual inspection of the results. Options are:<br>• Yes, include plots, default: Two images will be created, one with all parameters and the other restricted to parameters used for clustering. These images will include all pairwise combinations of the parameters with events color-coded depending on their cluster assignment. Note that creating the images for very high dimensional data may be more time-consuming than the actual clustering step. Consult the Usage section for more details.<br>• No, omit the plots: No plots will be created.
Seed	Seed value for the random generator. Provide any consistent value to obtain reproducible results.
Print.debug.info	Should additional debugging information be provided (e.g., this may be useful to debug cases where clustering does not work properly for certain data files). Default: No

Output Files

1. Subpopulations in separate CSV files

   The module outputs several CSV files, one for each of the identified cell sub-population. The measurements in these files correspond to cells assigned to the particular population. The columns of the CSV file correspond to the parameters of the input FCS file and the column headings will be created based on the short and long parameter names ($PnN and $PnS keyword values) as a single name separated by ":", i.e., $PnN:$PnS (for example: "FL2-H:CD69 PE"). The file names will be constructed as *[Input FCS file name]*_population_*n*.csv, where *n* is a number from 1 to the number of populations identified in the input FCS files.

2. CSV clustering results

   A clustering results file in the CSV format. It will store the population number for each event in a single file. The CSV file will contain a single column with the *Label* heading. Rows in the file will assign population labels (numbers) for events in the

input FCS data file maintaining the same order of events as in FCS file. The file name will be constructed as *[Input FCS file name]*.clustering.results.csv.

3. CLS clustering results

   A clustering results file in the CLS format, which will store the population number for each event in a single file. The order of the events is the same as in the original FCS file. The population numbers are from 1 to the number of populations identified in the input FCS file. The file name will be constructed as *[Input FCS file name]*.clustering.results.cls.

4. Clustering results images

   If requested by the *Draw.plots* parameter, two images will be created, one with all parameters and the other restricted to parameters used for clustering. These images will be saved in the PNG format and will include all pairwise combinations of the parameters with events color-coded depending on their cluster assignment. The file names will be created as *[Input FCS file name]*.results.image.all.parameters.png and *[Input FCS file name]*.results.image.clustered.parameters.png, respectively.

Platform Dependencies

Module type: Flow Cytometry

CPU type: any

OS: any

Language: R 2.11

GenePattern Module Version Notes

Date	Version	Description
07/13/12	v.1	Initial Version
