# Supplementary material for: GenePattern flow cytometry suite
Source: Source Code Biol Med. 2013 Jul 3;8:14. doi: 10.1186/1751-0473-8-14 (PMC3717030; doi:10.1186/1751-0473-8-14)
Supplement: Additional file 2 — GenePattern Flow Cytometry Suite Modules. Please use any ZIP-compatible software to extract the ZIP archive file into a folder. After extraction, the folder will contain 34 ZIP files, each of these represents one of the GenePattern modules in the GenePattern Flow Cytometry Suite. If you are hosting your own GenePattern server then you can install the GenePattern Flow Cytometry Suite from these module ZIP files by following “Modules & Pipelines”, “Install from zip” at your local GenePattern site. The latest version of these modules can always be obtained by navigating to the particular module at the GenePattern public server web site ( http://genepattern.broadinstitute.org/gp/) and following the “export” link. [file 1751-0473-8-14-S2.zip › AdditionalFile2_GPFCMSuiteModules/FlowMergeCluster.zip!/FlowMergeCluster.pdf]

GenePattern

FlowMergeCluster Documentation

Description: Clustering of flow cytometry data using the FlowMerge algorithm .

Author: Josef Spidlen, jspidlen@bccrc.ca

Please see the gp-flowcyt-help Google Group (https://groups.google.com/a/broadinstitute.org/forum/#!forum/gp-flowcyt-help) for help regarding these modules. If you have a GenePattern specific question, please feel free to contact GenePattern at gp-help@broadinstitute.org

Summary

This module uses the FlowMerge cluster merging approach to perform automated gating of cell populations in flow cytometry data. The max BIC model fitting criterion for mixture models generally overestimates the number of cell populations in flow cytometry data because the number of mixture components required to accurately model a distribution is usually greater than the number of distinct cell populations. Model fitting criteria based on the entropy, such as the ICL, provide better estimates of the number of clusters but tend to provide a poor fit to the underlying distribution.

FlowMerge combines these two approaches by merging mixture components from the max BIC fit based on an entropy criterion. This approach allows multiple mixture components to represent the same cell subpopulation. Merged clusters are mixtures themselves and are summarized by a weighted combination of their component model parameters. The result is a mixture model that retains the good model fitting properties of the max BIC solution but the number of components more closely reflects the true number of distinct cell subpopulations.

For more information on the FCS file format, see the FCS 3.1 File Standard (PDF).

Usage

Maximum memory and processing time was estimated based on clustering several large FCS files. Please note that the run time may decrease with increased number of computing nodes (as long as the server has appropriate processors/cores available for computing); however, the memory requirements increase significantly (nearly linearly with the number of nodes). The run time is also directly dependent on the range of clusters that is being searched for.

- Clustering 8 dimensions from an FCS file with 200,000 events; searching for the range of 1-5 clusters with 4 computing nodes: RAM: 2.1 GB, run time: 1 hour, 30 minutes.
- Clustering 6 dimensions from an FCS file with 150,000 events; searching for the range of 1-10 clusters with 4 computing nodes: RAM: 1.4 GB, run time: 30 minutes.

- Clustering 6 dimensions from an FCS file with 150,000 events; searching for the range of 1-10 clusters with 1 computing nodes: RAM: 400 MB, run time: 1 hour, 50 minutes.

References

Greg Finak and Raphael Gottardo. Merging mixture components for cell population identification in flow cytometry data - the flowmerge package. Accessed March 2010. http://www.bioconductor.org/packages/bioc/html/flowMerge.html.

GenePattern. The CLS file format, accessed November 2009. http://www.broadinstitute.org/cancer/software/genepattern/tutorial/gp_fileformats.html

Parks DR, Roederer M, Moore WA. A new "logicle" display method avoids deceptive effects of logarithmic scaling for low signals and compensated data. *Cytometry A*. 2006;69(6):541–551.

Parameters

Name	Description
Input FCS data file<br>(required)	The FCS file to be clustered.
Dimensions	A comma-separated list of dimensions (flow cytometry parameters/channels) to be used for clustering. The module accepts both a list of parameter names (e.g., FSC-H, SSC-H, FL1-H, FL4-H) as well as a list of parameter indexes (e.g., 1,2,4,5,8). All dimensions but Time will be used if the Dimensions parameter is not provided.

Transformation (required)	Which transformation to apply prior clustering. Fluorescence channels are usually better visualized and clustered using a transformation. Usually, the better the data looks visually, the better the clustering results of this module. However, note that applying a transformation where a high curvature region of the transformation coincides with regions of non near zero density of events can also generate spurious populations. You can use one of the following:<br>• ASinH (Hyperbolic Arcus Sine), default – The ASinH transformation produces good results on most data.<br>• Logarithmic transformation – The logarithmic transformation can be used of not too much data (or no data of interest) is located around the axes.<br>• Logicle transformation – Logicle transformation is an alternative to logarithmic transformation that better handles data around the axes.<br>• No transformation – The data will be used as stored in the FCS data file.
Dimensions to transform	A comma-separated list of dimensions (channels) that shall transformed as specified by previous parameter. This will be ignored if no transformation is specified above. If this parameter is not provided and transformation is specified above, the algorithm will use heuristics to identify parameters that shall be transformed. These heuristics are based on how parameters are stored in the FCS file, their resolution and their name. Again, you can use either parameter names or parameter indexes to specify dimensions to transform.
Range for number of clusters	The range for the number of subpopulations (clusters) that FlowMerge will search for. FlowMerge will try to pick the best number of clusters from the specified range, which shall be provided in the min-max format, where both, mix and max are integers and min is smaller than max. Please note that increased range increases the computing time for this module. Default: 1-10

Estimate degrees of freedom (required)	An indication whether to estimate the degrees of freedom used for the t distribution when modeling data. You can use one of the following:<br>• No estimation (default): The value provided by the Degrees of freedom parameter will be used.<br>• Estimate: The degrees of freedom will be estimated; the value of the Degrees of freedom parameter will be ignored.<br>• Estimate separately for each cluster: The degrees of freedom will be estimated separately for each cluster; the value of the Degrees of freedom parameter will be ignored.
Degrees of freedom	The degrees of freedom used for the t distribution when modeling data. The value of the Degrees of freedom parameter will be ignored if estimation is requested by the Estimate degrees of freedom parameter. Gaussian distribution will be used if Degrees of freedom are not provided and estimation is not requested. Default: 4
Number of computing nodes (required)	How many nodes (e.g., processors, cores) to use if you wish to run the analysis in a parallel mode? Enter '1' if you wish do NOT want to use the parallel mode. Enter a number higher than 1 if your server/cluster has multiple computers/processors/cores and you want to utilize several of these for FlowMerge clustering. Note that the run time may decrease with increased number of computing nodes (as long as the server has appropriate processors/cores available for computing); however, the memory requirements increase significantly since each of the computing nodes will calculate in its own computing environment. Default: 1 (no parallelism, default)

Input Files

1. Input FCS data file

   The FCS file to be clustered, i.e., events/cells automatically separated into subpopulations.

Output Files

1. Subpopulations in separate CSV files

   The module outputs several CSV files, one for each of the identified cell subpopulations. The measurements in these files correspond to cells assigned to the particular population. The columns of the CSV file correspond to the parameters of the input FCS file and the column headings will be created based on the short and

long parameter names ($PnN and $PnS keyword values) as a single name separated by ”:”, i.e., $PnN:$PnS, for example: ”FL2-H:CD69 PE”. The file names will be constructed as <Input FCS file name>_population_<n>.csv, where <Input FCS file name> is the name of your input file, and <n> is a number from 0 to the number of populations identified in the input FCS files. The population numbered as 0 lists unassigned cell measurements (i.e., identified as outliers).

2. CSV clustering results

   A clustering results file in the CSV format, which stores the population number for each event in a single file. The CSV file contains a single column with the Label (0 is outlier) heading. Rows in the file will assign population labels (numbers) for events in the input FCS data file maintaining the same order of events as in FCS file. The population numbers are from 0 to the number of populations identified in the input FCS files. The population numbered as 0 lists unassigned cell measurements (i.e., identified as outliers). The file name will be constructed as <Input FCS file name>.clustering.results.csv.

3. CLS clustering results

   A clustering results file in the CLS format, which stores the population number for each event in a single file. The order of the events is the same as in the original FCS file. The population numbers are from 0 to the number of populations identified in the input FCS files. The population numbered as 0 lists unassigned cell measurements (i.e., identified as outliers). The file name will be constructed as <Input FCS file name>.clustering.results.cls.

4. Clustering uncertainty
   A clustering uncertainty overview file in CSV format, which stores the cluster assignment uncertainty (as percentage) for each event in the input data file. The CSV file will contain two columns with the Event number and Cluster assignment uncertainty (%) headings. Rows in the file will report the cluster assignment uncertainty for all events, where uncertainty is defined as 100% minus the posterior probability that an event (data point) belongs to the cluster to which it is assigned. A value of NA will be reported for events that have not been assigned to any cluster (reported as outliers). The event order is maintained from the input FCS data file. The file name will be constructed as <Input FCS file name>.clustering.results. uncertainty.csv.
5. Clustering label probability
   A CSV file reporting the probability of being a member of each of the population for each of the assigned events. The CSV file contains K +1 columns, where K is the number of identified cell populations (labels). The columns will have the following headings: Event Number, Probability of being population 1 (%), ..., Probability of being population K (%). The data in the file will list the event number in the first column (maintaining the order of events from the input FCS data file), and the probability of being member of each of the populations in additional columns. A value NA indicates that an event is considered as outlier and has not been assigned to any population. The file name will be constructed as <Input FCS file name>.clustering.label.probability.csv.
6. Clustering results images
   A PDF file graphically showing the clustering results in all pairwise combinations of all the dimensions (channels) used for clustering. Each page in the PDF file will contain one graph (i.e., one combination of dimensions), a dot plot with color-coded events based on cluster assignment as well as curves illustrating the shapes of the

clusters. Please note that these images may not be very informative since high-dimensional clustering results may not show well in any of the two-dimensional projections (i.e, the cell populations may not be separated in any of the two-dimensional subspaces even though they are separated in the high dimensional space used for clustering). The file name will be constructed as <Input FCS file name>.clustering.results.images.pdf.

7. Entropy of clustering image
   A PNG image file showing a graph of the entropy of clustering versus the cumulative number of merged observations for various numbers of clusters. FlowMerge fits a piece-wise linear function to this graph in order to estimate the best number of clusters. See documentation of FlowMerge for more details. The name of the file will be constructed as <Input FCS file name>.entropy.of.clustering.image.png.

Example Data

GvHD1.001.fcs is included in the module source codes; it can be run with

Dimensions: FL1-H,FL2-H,FL3-H,FL4-H

Transformation: AsinH (i.e, keep default)

Dimensions to transform: Keep empty

Range for number of clusters: 1-6

Estimate degrees of freedom: No Estimation (i.e, keep default)

Degrees of freedom: 4 (i.e, keep default)

Number of computing nodes: 4

Please allow a few minutes for the clustering to complete.

Platform Dependencies

**Module type:**	Flow Cytometry
**CPU type:**	Any
**OS:**	Any
**Language:**	R 2.10

GenePattern Module Version Notes

Version	Description
1	Initial release 7/11/12.
