# Supplementary material for: GenePattern flow cytometry suite
Source: Source Code Biol Med. 2013 Jul 3;8:14. doi: 10.1186/1751-0473-8-14 (PMC3717030; doi:10.1186/1751-0473-8-14)
Supplement: Additional file 2 — GenePattern Flow Cytometry Suite Modules. Please use any ZIP-compatible software to extract the ZIP archive file into a folder. After extraction, the folder will contain 34 ZIP files, each of these represents one of the GenePattern modules in the GenePattern Flow Cytometry Suite. If you are hosting your own GenePattern server then you can install the GenePattern Flow Cytometry Suite from these module ZIP files by following “Modules & Pipelines”, “Install from zip” at your local GenePattern site. The latest version of these modules can always be obtained by navigating to the particular module at the GenePattern public server web site ( http://genepattern.broadinstitute.org/gp/) and following the “export” link. [file 1751-0473-8-14-S2.zip › AdditionalFile2_GPFCMSuiteModules/ImmPortCSV2TXT.zip!/ImmPortCSV2TXT.pdf]

ImmPortCSV2TXT Documentation

Description: Conversion of a CSV file to a tab-delimited text file.

Author: Yu “Max” Qian (qianyu_cs@yahoo.com)

Please see the gp-flowcyt-help Google Group (https://groups.google.com/a/broadinstitute.org/forum/#!forum/gp-flowcyt-help) for help regarding these modules. If you have a GenePattern specific question, please feel free to contact GenePattern at gp-help@broadinstitute.org

Summary

The module will convert a CSV file to a matrix-like tab-delimited text file that can be further used as input for modules such as *ImmPortColSelection*, *ImmPortFLOCK*, or the *ImmPortCrossSample* module. In general, commas from the CSV file will be replaced by tab-delimiters in the output file.

References

Qian, Y., Wei, C., Eun-Hyung Lee, F., Campbell, J., Halliley, J., Lee, J. A., Cai, J., Kong, Y. M., Sadat, E., Thomson, E., Dunn, P., Seegmiller, A. C., Karandikar, N. J., Tipton, C. M., Mosmann, T., Sanz, I. and Scheuermann, R. H. (2010), Elucidation of seventeen human peripheral blood B- cell subsets and quantification of the tetanus response using a density-based method for the automated identification of cell populations in multidimensional flow cytometry data. Cytometry Part B: Clinical Cytometry, 78B: S69 S82. doi: 10.1002/cyto.b.20554

Parameters

Name	Description
*Input CSV	The input CSV file that will be converted to a tab-delimited text data file file. Default: none

Input Files

1. Input CSV data file

   The input CSV file that will be converted to a tab-delimited text file.

Output Files

1. Output data in a text file

   A tab-delimited text file named output.txt containing the data matrix from the input CSV file.

Example Data

Run the module with Input.file = Selected_Columns_CKJN_A02.csv

Platform Dependencies

Module type: Flow Cytometry

CPU type: Any

OS: Any

Language: C

GenePattern Module Version Notes

Date	Version	Description
09/24/12	v.1	Initial Version
