# Supplementary material for: GenePattern flow cytometry suite
Source: Source Code Biol Med. 2013 Jul 3;8:14. doi: 10.1186/1751-0473-8-14 (PMC3717030; doi:10.1186/1751-0473-8-14)
Supplement: Additional file 2 — GenePattern Flow Cytometry Suite Modules. Please use any ZIP-compatible software to extract the ZIP archive file into a folder. After extraction, the folder will contain 34 ZIP files, each of these represents one of the GenePattern modules in the GenePattern Flow Cytometry Suite. If you are hosting your own GenePattern server then you can install the GenePattern Flow Cytometry Suite from these module ZIP files by following “Modules & Pipelines”, “Install from zip” at your local GenePattern site. The latest version of these modules can always be obtained by navigating to the particular module at the GenePattern public server web site ( http://genepattern.broadinstitute.org/gp/) and following the “export” link. [file 1751-0473-8-14-S2.zip › AdditionalFile2_GPFCMSuiteModules/ImmPortColSelection.zip!/ImmPortColSelection.pdf]

ImmPortColSelection Documentation

Description: Selection of specified columns from a tab-delimited text file.

Author: Yu “Max” Qian (qianyu_cs@yahoo.com)

Please see the gp-flowcyt-help Google Group (https://groups.google.com/a/broadinstitute.org/forum/#!forum/gp-flowcyt-help) for help regarding these modules. If you have a GenePattern specific question, please feel free to contact GenePattern at gp-help@broadinstitute.org

Summary

This module is useful as a preprocessing step of flow cytometry data analysis because some columns in a FCS converted txt file (e.g., Time parameter is usually not needed in the data analysis and may need to be removed. The module takes a data table (a matrix-like tab-delimited text file) and outputs a user-specified subset (enumerated columns) of the data table in the same matrix-like tab-delimited text file format.

References

Qian, Y., Wei, C., Eun-Hyung Lee, F., Campbell, J., Halliley, J., Lee, J. A., Cai, J., Kong, Y. M., Sadat, E., Thomson, E., Dunn, P., Seegmiller, A. C., Karandikar, N. J., Tipton, C. M., Mosmann, T., Sanz, I. and Scheuermann, R. H. (2010), Elucidation of seventeen human peripheral blood B- cell subsets and quantification of the tetanus response using a density-based method for the automated identification of cell populations in multidimensional flow cytometry data. Cytometry Part B: Clinical Cytometry, 78B: S69 S82. doi: 10.1002/cyto.b.20554

Parameters

Name	Description
*Input file	A data table, i.e., a tab-delimited text file with columns corresponding to parameters and rows to events. The first row is a heading labeling parameters. Such a text file is supposed to be generated from an FCS file by the *ImmPortFCSConvLogicleTrans* module.
*Dimension specification	A list of dimensions/columns to extract, specified by their indexes (starting from 1), e.g., 1,4,6. There should not be any white space characters (e.g, spaces, tabs, new lines, etc.) in the parameter value.

Input Files

1. Input file

   A tab-delimited text file with columns corresponding to parameters and rows to events. The first row is a heading labeling parameters. The text file is supposed to be generated from an FCS file (e.g., by the ImmPortFCSConvLogicleTrans module).

Output Files

1. Output data in a text file

   A tab-delimited text file named output.txt containing user-specified columns from the input text file.

Example Data

Run the module with Input.file = CKJN_A02.txt
Dimension.specification = 1,2,3,5,8,9,12

Platform Dependencies

**Module type:**	Flow Cytometry
**CPU type:**	Any
**OS:**	Any
**Language:**	C

GenePattern Module Version Notes

Date	Version	Description
09/20/12	v.1	Initial Version
