# Supplementary material for: GenePattern flow cytometry suite
Source: Source Code Biol Med. 2013 Jul 3;8:14. doi: 10.1186/1751-0473-8-14 (PMC3717030; doi:10.1186/1751-0473-8-14)
Supplement: Additional file 2 — GenePattern Flow Cytometry Suite Modules. Please use any ZIP-compatible software to extract the ZIP archive file into a folder. After extraction, the folder will contain 34 ZIP files, each of these represents one of the GenePattern modules in the GenePattern Flow Cytometry Suite. If you are hosting your own GenePattern server then you can install the GenePattern Flow Cytometry Suite from these module ZIP files by following “Modules & Pipelines”, “Install from zip” at your local GenePattern site. The latest version of these modules can always be obtained by navigating to the particular module at the GenePattern public server web site ( http://genepattern.broadinstitute.org/gp/) and following the “export” link. [file 1751-0473-8-14-S2.zip › AdditionalFile2_GPFCMSuiteModules/ImmPortCrossSample.zip!/ImmPortCrossSample.pdf]

GenePattern

ImmPortCrossSample Documentation

Description: Centroid-based cross-sample comparison of cell populations identified by FLOCK

Author: Yu "Max" Qian (qianyu_cs@yahoo.com)

Please see the gp-flowcyt-help Google Group (https://groups.google.com/a/broadinstitute.org/forum/#!forum/gp-flowcyt-help) for help regarding these modules. If you have a GenePattern specific question, please feel free to contact GenePattern at gp-help@broadinstitute.org

Summary

This module will perform clustering of flow cytometry data with a known specified number and position of sub populations. Flow cytometry data shall be provided in a matrix-like tab-delimited text file. This may be produced either by conversion from FCS using the *ImmPortFCSConvLogicleTrans* module (optionally followed by the *ImmPortColSelection* module), or by conversion from CSV using the *ImmPortCSV2TXT* module. Positions of the populations are provided with a centroid file generated by the ImmPortFLOCK module, which will typically be executed on a different data file being compared with.

References

Qian, Y., Wei, C., Eun-Hyung Lee, F., Campbell, J., Halliley, J., Lee, J. A., Cai, J., Kong, Y. M., Sadat, E., Thomson, E., Dunn, P., Seegmiller, A. C., Karandikar, N. J., Tipton, C. M., Mosmann, T., Sanz, I. and Scheuermann, R. H. (2010), Elucidation of seventeen human peripheral blood B- cell subsets and quantification of the tetanus response using a density-based method for the automated identification of cell populations in multidimensional flow cytometry data. Cytometry Part B: Clinical Cytometry, 78B: S69 S82. doi: 10.1002/cyto.b.20554

Parameters

Name	Description
*Input file	A data table, i.e., a tab-delimited text file with columns corresponding to parameters and rows to events. The first row is a heading labeling parameters. Such a text file is supposed to be generated from an FCS file by the *ImmPortFCSConvLogicleTrans* module.
*Centroid file	A text file specifying the position of each of the cell populations. Such a text file is supposed to be generated by the *ImmPortFLOCK* module (file population_center.txt).

*Number of iterations	The number of iterations that the algorithm will use to match events with the closest population. The default value of 1 usually works well for samples that are similar to each other. The value may be increased up to 10 to process more heterogeneous sets of samples. Please note that increasing the number of iterations will linearly increase the time of execution of the module.

Input Files

1. Input file

   A data table, i.e., a tab-delimited text file with columns corresponding to parameters and rows to events. The first row is a heading labeling parameters. The text file is supposed to be generated from an FCS file by the *ImmPortFCSConvLogicleTrans* module.

Output Files

1. Expression profiles

   A tab-delimited text file named profile.txt containing expression profiles (i.e., values from [1 to n] where n is commonly 4) of each population (in rows) for each marker (in columns)

2. Population assignment

   A text file named population id.txt containing population identifiers (i.e, values from [1 to n] where n is number of populations) corresponding to (and maintaining the order of) events in the input data file, one identifier per row.)

3. Population proportions

   A tab-delimited text file named percentage.txt containing two columns: Population ID and Percentage. Rows after the header include the population identifiers and percentage of events within that population (relatively to the whole data file)

4. Mean fluorescence intensities

   A tab-delimited text file named MFI.txt containing the population identifiers in the first column (i.e, values from [1 to n] where n is number of populations), and the mean fluorescence intensity of that particular population for each of the markers in additional columns. The order of the markers (also referred to as parameters or channels) is maintained from the input data file.

Example Data

Run the module with Input.file = Selected_Columns_CKJN_A05.txt, Centroid.file = population_center.txt, Number.of.iterations = 1

GenePattern

Platform Dependencies

Module type: Flow Cytometry

CPU type: Any

OS: Any

Language: C

GenePattern Module Version Notes

Date	Version	Description
09/24/12	v.1	Initial Version
