# Supplementary material for: GenePattern flow cytometry suite
Source: Source Code Biol Med. 2013 Jul 3;8:14. doi: 10.1186/1751-0473-8-14 (PMC3717030; doi:10.1186/1751-0473-8-14)
Supplement: Additional file 2 — GenePattern Flow Cytometry Suite Modules. Please use any ZIP-compatible software to extract the ZIP archive file into a folder. After extraction, the folder will contain 34 ZIP files, each of these represents one of the GenePattern modules in the GenePattern Flow Cytometry Suite. If you are hosting your own GenePattern server then you can install the GenePattern Flow Cytometry Suite from these module ZIP files by following “Modules & Pipelines”, “Install from zip” at your local GenePattern site. The latest version of these modules can always be obtained by navigating to the particular module at the GenePattern public server web site ( http://genepattern.broadinstitute.org/gp/) and following the “export” link. [file 1751-0473-8-14-S2.zip › AdditionalFile2_GPFCMSuiteModules/ImmPortFCSConvLogicleTrans.zip!/ImmPortFCSConvLogicleTrans.pdf]

GenePattern

ImmPortFCSConvLogicleTrans Documentation

Description: Conversion of a binary FCS2.0 or FCS3.0 file to a tab-delimited text file on logicle scale.

Author: Yu "Max" Qian (qianyu_cs@yahoo.com), Yue Liu (yliu0@yahoo.com)

Please see the gp-flowcyt-help Google Group (https://groups.google.com/a/broadinstitute.org/forum/#!forum/gp-flowcyt-help) for help regarding these modules. If you have a GenePattern specific question, please feel free to contact GenePattern at gp-help@broadinstitute.org

Summary

This module performs a Logicle transformation of values stored in an FCS data file and saves these as a data table (a matrix-like tab-delimited text file) that can be read by the *ImmPortColSelection* module, *ImmPortFLOCK* module or by some third party software. The Logicle transformation is tweaked to perform similarly to the transformation built-in the FlowJo software tool (TreeStar, Inc).

References

Yu Qian, Yue Liu, John Campbell, Elizabeth Thomson, Megan Kong, Richard H. Scheuermann, FCSTrans: an open source software system for FCS file conversion and data transformation, Cytometry Part A, accepted, Feb 2012.

David R. Parks, Mario Roederer, and Wayne. A. Moore. A new "logicle" display method avoids deceptive effects of logarithmic scaling for low signals and compensated data. Cytometry A, 69(6):541-551, June 2006.

Josef Spidlen, Wayne Moore, David Parks, Michael Goldberg, Chris Bray, Pierre Bierre, Peter Gorombey, Bill Hyun, Mark Hubbard, Simon Lange, Ray Lefebvre, Robert R. Leif, David Novo, Leo Ostruszka, Adam Treister, James Wood, Robert F. Murphy, Mario Roederer, Damir Sudar, Robert Zigon, and Ryan R. Brinkman. Data file standard for flow cytometry, version fcs 3.1. Cytometry A, 77(1):97-100, 2010.

Parameters

Name	Description
*Input file	An FCS file that shall be transformed with the Logicle transformation, converted and saved as tab-delimited text file.

Input Files

1. Input file

   A binary FCS file (either FCS2.0 or FCS3.0 standard).

GenePattern

Output Files

1. A tab-delimited txt file containing the data matrix of cell marker expression values

   A tab-delimited text file that has been transformed with the Logicle transformation applied on the Input FCS file. The name of the result file is created based on the original input file name with the file extension being changed to .txt. The number of columns is the parameters measured in the flow cytometry experiment, including scatter parameters and fluorescence parameters. The number of rows is the number of events collected in the flow cytometry experiment.

Example Data

Run the module with Input.file = CKJN_A02.fcs

Platform Dependencies

**Module type:**	Flow Cytometry
**CPU type:**	Any
**OS:**	Any
**Language:**	R 2.11

GenePattern Module Version Notes

Date	Version	Description
09/20/12	v.1	Initial Version
