# Supplementary material for: GenePattern flow cytometry suite
Source: Source Code Biol Med. 2013 Jul 3;8:14. doi: 10.1186/1751-0473-8-14 (PMC3717030; doi:10.1186/1751-0473-8-14)
Supplement: Additional file 2 — GenePattern Flow Cytometry Suite Modules. Please use any ZIP-compatible software to extract the ZIP archive file into a folder. After extraction, the folder will contain 34 ZIP files, each of these represents one of the GenePattern modules in the GenePattern Flow Cytometry Suite. If you are hosting your own GenePattern server then you can install the GenePattern Flow Cytometry Suite from these module ZIP files by following “Modules & Pipelines”, “Install from zip” at your local GenePattern site. The latest version of these modules can always be obtained by navigating to the particular module at the GenePattern public server web site ( http://genepattern.broadinstitute.org/gp/) and following the “export” link. [file 1751-0473-8-14-S2.zip › AdditionalFile2_GPFCMSuiteModules/ImmPortFLOCK.zip!/ImmPortFLOCK.pdf]

ImmPortFLOCK Documentation

Description: FLOCK (FLOw cytometry Clustering without K): a density-based clustering method for automated population identification from multi-dimensional flow cytometry data

Author: Yu "Max" Qian (qianyu_cs@yahoo.com)

Please see the gp-flowcyt-help Google Group (https://groups.google.com/a/broadinstitute.org/forum/#!forum/gp-flowcyt-help) for help regarding these modules. If you have a GenePattern specific question, please feel free to contact GenePattern at gp-help@broadinstitute.org

Summary

This module performs density-based clustering of multi-dimensional flow cytometry data. It is the main module of the ImmPort FLOCK flow cytometry analysis pipeline. Flow cytometry data shall be provided in a matrix-like tab-delimited text file. This may be produced either by conversion from binary FCS files using the *ImmPortFCSConvLogicleTrans* module (optionally followed by the *ImmPortColSelection* module if marker selection is necessary), or by conversion using the *ImmPortCSV2TXT* module if the input is a CSV file.

This is the version 1.0 of FLOCK. For version 2.0 or the newest version of FLOCK which is under testing, the user can find the source code at: http://immportflock.sourceforge.net or email the author.

References

Qian, Y., Wei, C., Eun-Hyung Lee, F., Campbell, J., Halliley, J., Lee, J. A., Cai, J., Kong, Y. M., Sadat, E., Thomson, E., Dunn, P., Seegmiller, A. C., Karandikar, N. J., Tipton, C. M., Mosmann, T., Sanz, I. and Scheuermann, R. H. (2010), Elucidation of seventeen human peripheral blood B-cell subsets and quantification of the tetanus response using a density-based method for the automated identification of cell populations in multidimensional flow cytometry data. Cytometry Part B: Clinical Cytometry, 78B: S69–S82. doi: 10.1002/cyto.b.20554

Parameters

Name	Description
*Input file	A data table, i.e., a tab-delimited text file with columns corresponding to parameters and rows to events. The first row is a heading labeling parameters. Such a text file is supposed to be generated from an FCS file by the ImmPortFCSConvLogicleTrans module optionally followed by the ImmPortColSelection module (if applicable to remove unused parameters). Alternatively, this text file may be created by the ImmPortCSV2TXT module if the original data is in a CSV format. Default: none

*Number of bins	The number of bins used to partition each dimension. The default value of 0 means that the algorithm will automatically determine a suitable number of bins. Any value from the [6, 29] range may be provided to manually override algorithmic estimations. Default: 0
*Density threshold	The minimal number of events in a bin (in the hyperspace of used markers) needed to mark the bin as a dense bin. The default value of 0 means that the algorithm will automatically determine a suitable density threshold. Any value from the [3, 99] range may be provided to manually override algorithmic estimations. Default: 0
*Maximum number of populations	The maximum number of populations identifiable by the clustering algorithm. The default value of 30 works well in most cases but may be overridden by any value from the [5, 999] range. Larger values may increase the execution time of the module. Default: 30

Input Files

1. Input file

   A TXT data file containing the data matrix of cell marker expression values. The number of columns is the parameters measured in the flow cytometry experiment, including scatter parameters and fluorescence parameters. The number of rows is the number of events collected in the flow cytometry experiment.

Output Files

1. Expression profiles

   A tab-delimited text file named profile.txt containing expression profiles (i.e., values from [1 to n] where n is commonly 4) of each population (in rows) for each marker (in columns)

2. Population assignment

   A text file named population id.txt containing population identifiers (i.e, values from [1 to n] where n is number of populations) corresponding to (and maintaining the order of) events in the input data file, one identifier per row.)

3. Population proportions

   A tab-delimited text file named percentage.txt containing two columns: Population ID and Percentage. Rows after the header include the population identifiers and percentage of events within that population (relatively to the whole data file)

4. Mean fluorescence intensities

   A tab-delimited text file named MFI.txt containing the population identifiers in the first column (i.e, values from [1 to n] where n is number of populations), and the mean fluorescence intensity of that particular population for each of the markers in

additional columns. The order of the markers (also referred to as parameters or channels) is maintained from the input data file.

5. Population center

   A text file named population center.txt containing the centroid coordinates of each identified population. This file may be used with the ImmPortCrossSample module to compare several data files to each other.

6. Algorithm parameters

   A tab-delimited text file named parameters.txt providing details about the parameters used within the clustering algorithm. These include the minimum and maximum values in input data, the actual density threshold and number of bins.

7. Clustering results

   A tab-delimited text file named flock results.txt created as a combination of the input file, event identifiers and population identifiers.

8. FCS properties

   A text file named fcs.properties containing the number of events, number of populations, and number of markers, as well as the algorithm parameters used in the analysis.

9. Event coordinates

   A text file named coordinates.txt containing ASCII-encoded coordinates of events that may be used for visualization purposes.

Example Data

Run the module with Input.file = Selected_Columns_CKJN_A02.txt
Number.of.bins = 0 Density.threshold = 0 Maximum.number.of.populations = 30

The module can be run with default values of all parameters.

Platform Dependencies

**Module type:**	Flow Cytometry
**CPU type:**	Any
**OS:**	Any
**Language:**	C

GenePattern Module Version Notes

Date	Version	Description
09/20/12	v.1	Initial Version
