# Supplementary material for: GenePattern flow cytometry suite
Source: Source Code Biol Med. 2013 Jul 3;8:14. doi: 10.1186/1751-0473-8-14 (PMC3717030; doi:10.1186/1751-0473-8-14)
Supplement: Additional file 2 — GenePattern Flow Cytometry Suite Modules. Please use any ZIP-compatible software to extract the ZIP archive file into a folder. After extraction, the folder will contain 34 ZIP files, each of these represents one of the GenePattern modules in the GenePattern Flow Cytometry Suite. If you are hosting your own GenePattern server then you can install the GenePattern Flow Cytometry Suite from these module ZIP files by following “Modules & Pipelines”, “Install from zip” at your local GenePattern site. The latest version of these modules can always be obtained by navigating to the particular module at the GenePattern public server web site ( http://genepattern.broadinstitute.org/gp/) and following the “export” link. [file 1751-0473-8-14-S2.zip › AdditionalFile2_GPFCMSuiteModules/KMeansClassifyFCS.zip!/KMeansClassifyFCS.pdf]

KMeansClassifyFCS Documentation

Description: Use the K-Means algorithm to find populations in an FCS file.

Author: Aaron Barsky,
Josef Spidlen, jspidlen@bccrc.ca,
Please see the gp-flowcyt-help Google Group (https://groups.google.com/a/broadinstitute.org/forum/#!forum/gp-flowcyt-help) for help regarding these modules. If you have a GenePattern specific question, please feel free to contact GenePattern at gp-help@broadinstitute.org

Summary

The KMeansClassifyFCS module automatically assigns cells to populations by clustering FCS data. The K-Means algorithm assigns every event in an FCS file to one of K different clusters. This algorithm requires a parameter, K, which specifies how many populations the algorithm shall be looking for. If you do not know in advance how many populations you expect to find, you can use the SuggestNumberOfPopulations module to estimate a K value.

K-Means is most effective for flow cytometry data when all clustering parameters use the same scale. It is common to use K-Means on the forward and side scatter; however, it may be problematic when mixing fluorescent parameters with scatters, especially when these are stored on different scales. You may want transform the parameters if this is the case. K-Means clustering can be applied on a single parameter.

The module outputs K files containing the cells in each of the populations. It also produces a .CLS file that identifies a cluster number for each cell without separating the cells in to different files.

Usage

The time and memory requirements were evaluated using an Intel(R) Core(TM)2 Extreme CPU X9650 @ 3.00GHz and the following tests:

- Clustering of an FCS file with 1,000,000 events; the search was performed to

find 3 clusters using 20 out of 21 FCS parameters. Required RAM: 475 MB.
Run time: 3 minutes.

- Clustering of an FCS file with 1,000,000 events; the search was performed to find 10 clusters using 23 out of 24 FCS parameters. Required RAM: 840 MB.
Run time: 4 minutes.

References

GenePattern. The cls file format, accessed November 2009.
http://www.broadinstitute.org/cancer/software/genepattern/tutorial/gp_fileformats.html

Parameters

Name	Description
K*	The number of populations that the algorithm shall be looking for. If you do not know the K value, you can use the SuggestNumberOfPopulations module to estimate it.
CSV file name	The ”base” name of the output population files in the CSV file format. Each population file will have the name CSV file name-n.csv, where n is a number from 1 to K, and CSV file name is the value of this parameter.
CLS file name	The name of the CLS output file, which will store the population number for each data point.
Dimensions	A comma-separated list of short parameter names ($PnN values) that shall be used to classify the populations.

Input Files

1. Input FCS data file

The FCS file to be clustered, i.e., events/cells automatically separated into subpopulations.

Output Files

1. Subpopulations in separate files

   The module outputs K CSV files containing the cells in each of the populations. The columns of the CSV file correspond to the parameters of the FCS file and the column headings will be created based on the short and long parameter names ($PnN and $PnS keyword values) as a single name separated by ";", i.e., $PnN:$PnS, for example: "FL2-H:CD69 PE". The file names will be constructed as CSV file name-n.csv, where n is a number from 1 to K, and CSV file name is the value of the CSV file name parameter.

2. CLS classification results

   A classification results file in the CLS format, which will store the population number for each event in a single file. The order of the events is the same as in the original FCS file. The population numbers are from 1 to K.

Example Data

t1.fcs is included in the module source codes; it can be run with K = 4.

Platform Dependencies

**Module type:**	Flow Cytometry
**CPU type:**	Any
**OS:**	Any
**Language:**	R 2.10

GenePattern Module Version Notes

**Date**	**Version**	**Description**
7/11/2012	v.1	Initial version
