# Supplementary material for: GenePattern flow cytometry suite
Source: Source Code Biol Med. 2013 Jul 3;8:14. doi: 10.1186/1751-0473-8-14 (PMC3717030; doi:10.1186/1751-0473-8-14)
Supplement: Additional file 2 — GenePattern Flow Cytometry Suite Modules. Please use any ZIP-compatible software to extract the ZIP archive file into a folder. After extraction, the folder will contain 34 ZIP files, each of these represents one of the GenePattern modules in the GenePattern Flow Cytometry Suite. If you are hosting your own GenePattern server then you can install the GenePattern Flow Cytometry Suite from these module ZIP files by following “Modules & Pipelines”, “Install from zip” at your local GenePattern site. The latest version of these modules can always be obtained by navigating to the particular module at the GenePattern public server web site ( http://genepattern.broadinstitute.org/gp/) and following the “export” link. [file 1751-0473-8-14-S2.zip › AdditionalFile2_GPFCMSuiteModules/LogicleTransformFCS.zip!/LogicleTransformFCS.pdf]

GenePattern

LogicleTransformFCS Documentation

Description: Logicle transformation of (selected) parameters in a list mode Flow Cytometry Standard (FCS) data file.

Author: Josef Spidlen (jspidlen@bccrc.ca), gp-help@broadinstitute.org

Please see the gp-flowcyt-help Google Group (https://groups.google.com/a/broadinstitute.org/forum/#!forum/gp-flowcyt-help) for help regarding these modules. If you have a GenePattern specific question, please feel free to contact GenePattern at gp-help@broadinstitute.org

Summary

The module takes an input FCS data file and applies the Logicle transformation on selected parameters.

In most flow cytometry applications, fluorescence signals of interest can range down to essentially zero. After fluorescence compensation, some cell populations will have low means and include events with negative data values. Logarithmic presentation has been very useful in providing informative displays of wide-ranging flow cytometry data, but it fails to adequately display cell populations with low means and high variances and, in particular, offers no way to include negative data values. This has led to a great deal of difficulty in interpreting and understanding flow cytometry data, has often resulted in incorrect delineation of cell populations, and has led many people to question the correctness of compensation computations that were, in fact, correct. The Logicle display method provides more complete, appropriate, and readily interpretable representations of data that includes populations with low-to-zero means, including distributions resulting from fluorescence compensation procedures, than can be produced using either logarithmic or linear displays.

For more information on the FCS file format, see the FCS 3.1 File Standard (PDF).

Usage

Maximum memory and processing time was estimated based on processing an FCS file with 1,000,000 events and 24 parameters stored as FCS 3.0 in the floating point data type.

- Maximum RAM: 1.9 GB
- Maximum run time: 30 seconds

References

Parks DR, Roederer M, Moore WA. A new "logicle" display method avoids deceptive effects of logarithmic scaling for low signals and compensated data. *Cytometry A*. 2006;69:541-551.

Spidlen J, Moore W, Parks D, Goldberg M, Bray C, Bierre P, Gorombey P, Hyun B, Hubbard M, Lange S, Lefebvre R, Leif R, Novo D, Ostruszka L, Treister A, Wood J,

Murphy RF, Roederer M, Sudar D, Zigon R, Brinkman RR. Data file standard for flow cytometry, version FCS 3.1. *Cytometry A*. 2010;77:97-100.

Parameters

Name	Description
Input.fcs.data.file	The FCS data file to transform.
Parameters.to. transform	A comma-separated list of FCS parameters to transform. The parameters are specified by their short names (i.e., values of the $PnN keyword). If nothing is specified, all parameters with FL in their short names will be transformed.
T	T > 0 is the data value at the top of the scale range to be displayed, e.g., 10,000 for common 4 decade data or 262144 (2^{18}) for an 18 bit data range. The maximum input value will be used if no explicit value for T is provided.
M	M > 0 is the full width of the intended Logicle display in asymptotic decades. The default value of 4.5 (decades) will be used. Note also that for the resulting *Logicle* transformation, *Logicle* (T) = M.
W	W > 0 is the linearization width in asymptotic decades. This specifies how wide the near-linear region will be on the plot and also determines the slope of the scale at the data zero assuming that T, M, and A have already been defined. In a standard Logicle plot, the width of the displayed region below data zero is also W. This assures that the scale in the negative range does not depart greatly from linearity and thereby avoids the artifacts seen with log scaling of low data values.<br>A recommended way to specify W to match particular data is to select a value *r* approximating the most negative data value that must be included and calculate W as:<br>$W = \frac{M - \log(T / \lvert r \rvert)}{2}$<br>Setting *r* at the 5th percentile of events that are below zero will yield an appropriate display in most cases. The described calculation will be used to set W for each of the transformed FCS parameters if no explicit value for W is provided.

A	A is the additional negative display range in asymptotic decades. For a standard *Logicle* plot A = 0. In flow cytometry applications the use of positive values of A (additional negative range in view) is not recommended. If data of interest falls below the end of a standard *Logicle* plot, the appropriate remedy is to increase W to bring more negative values into the display range. In cases where low data values are dominated by statistical variation but the values are constrained to be non-negative (as seen in peak detected flow cytometry data), a *Logicle* plot with A = −W would include data zero and be near-linear at low data values thereby avoiding problems associated with log scales at the low end. Thus, negative values for A may be more likely to have valid uses than positive values. The default value of 0 will be used.
Use.Fast. Implementation	Use regular implementation (Default): Use regular implementation of Logicle with high precision, which may perform slower.<br>Use fast implementation: Use a fast implementation of Logicle with less precision. The typical maximum difference between value calculated by regular versus fast implementation are in the range of 10^{-4} . On the other hand, while the fast calculation is several times faster than the regular one, the I/O operations are typically the most time consuming aspect of the module. Therefore, with regular hard drive-based storage on the server, the overall time saved by the fast option is only about 10% in most cases.
Output.fcs.file. name	The output FCS file name. By default the input file name is used as the base name.

Output Files

1. FCS data file with Logicle transformation applied on selected parameters

   An FCS data file with selected parameters transformed by the Logicle transformation. The data is stored as floating points with $PnE/0,0/ indicating linearity; however, make sure to distinguish the parameters on the Logicle scale from parameters on the linear scale. For example, any compensation will be applied on linear data, i.e., prior to the Logicle transformation. The TRANSFORMATION DETAILS keyword is included to remind the user about applied transformations and to specify details of the transformations (which may be different for different parameters based on specific parameter values).

Platform Dependencies

Module type: Flow Cytometry

CPU type: any

OS: any

Language: Java (1.6 minimum)

GenePattern Module Version Notes

Date	Version	Description
7/11/2012	1	Initial version released
