# Supplementary material for: GenePattern flow cytometry suite
Source: Source Code Biol Med. 2013 Jul 3;8:14. doi: 10.1186/1751-0473-8-14 (PMC3717030; doi:10.1186/1751-0473-8-14)
Supplement: Additional file 2 — GenePattern Flow Cytometry Suite Modules. Please use any ZIP-compatible software to extract the ZIP archive file into a folder. After extraction, the folder will contain 34 ZIP files, each of these represents one of the GenePattern modules in the GenePattern Flow Cytometry Suite. If you are hosting your own GenePattern server then you can install the GenePattern Flow Cytometry Suite from these module ZIP files by following “Modules & Pipelines”, “Install from zip” at your local GenePattern site. The latest version of these modules can always be obtained by navigating to the particular module at the GenePattern public server web site ( http://genepattern.broadinstitute.org/gp/) and following the “export” link. [file 1751-0473-8-14-S2.zip › AdditionalFile2_GPFCMSuiteModules/MClustClusterLabel.zip!/MClustClusterLabel.pdf]

GenePattern

MClustClusterLabel Documentation

Description: Model-based labeling (matching) of clustered flow cytometry data.

Author: Josef Spidlen (jspidlen@bccrc.ca)

Please see the gp-flowcyt-help Google Group (https://groups.google.com/a/broadinstitute.org/forum/#!forum/gp-flowcyt-help) for help regarding these modules. If you have a GenePattern specific question, please feel free to contact GenePattern at gp-help@broadinstitute.org

Summary

Independent clustering of a several flow cytometry samples (e.g., blood from different patients) typically results in dividing each of the FCS files into several groups corresponding to cell subpopulations in each sample. Another analytical step is required to match (label) these subpopulations across different samples. For example, a cluster (subpopulation) number 1 of patient 1 can correspond to cluster (subpopulation) number 3 of patient 2. Such matching only makes sense if the original flow cytometry samples were treated the same way and stained with the same fluorescence markers (same fluorescence parameters). The label matching is usually done by comparison of the position (e.g., the mean) of the clusters (subpopulations) in the space of fluorescence parameters that have been used for the original clustering.

The MClustClusterLabel module can be used to assign labels to previously clustered data sets from multiple flow cytometry samples. The data may have been previously clustered by various clustering algorithms, including the FlowClust algorithm as implemented in the FlowClustClassify module. The main input for the MClustClusterLabel module is a set of CSV files with each subpopulation in a separate file. The columns of this CSV file describe the flow cytometry parameters of the cells (in rows) of the CSV file. The FlowClustClassify module outputs its results in this kind of CSV file (on top of its CLS output).

The cluster matching implemented in this module is performed using model-based clustering of means of the previously clustered subpopulations. The user has the choice of various models that can be used for this purpose. In addition, the user shall specify how many distinct labels (subpopulations) are expected to be found across all the previously clustered flow cytometry data. The MClustClusterLabelBIC module can be used in order to estimate this number. Finally, the cluster label matching can be done based on all flow cytometry parameters (i.e., all but time, default) or a subset of these flow cytometry parameters can be specified. Matching labels based on a subset of flow cytometry parameters is useful in case the original clustering has been performed using such a subset.

See MClust documentation (http://cran.r-project.org/web/packages/mclust/index.html) for details about different models.

Usage

Memory and processing time was estimated based on processing a set 118 pre-clustered subpopulations (118 CSV files) with 5 flow cytometry parameters each (6 MB of input data).

- RAM: 30 MB
- Run time: 5 seconds

Requirements

- GenePattern ≥3.2.1
- R ≥2.10
- mclust ≥3.4.1
- workflowCommons ≥0.1-1

References

Chris Fraley and Adrian E. Raftery. MClust version 3 for r: Normal mixture modeling and model-based clustering, September 2006. http://www.stat.washington.edu/research/reports/2006/tr504.pdf

Lo K, Brinkman RR, Gottardo R. Automated gating of flow cytometry data via robust model-based clustering. *Cytometry A*. 2008;73:321-332.

Parameters

Name	Description
Input clustering results (required)	The input CSV files with each subpopulation in a separate file. The column headings describe the flow cytometry parameters (i.e., parameter names). The rows of the CSV file include flow cytometry parameters of cells from a single sample assigned to a subpopulation by a preceding clustering step. For example, multiple runs of the FlowClustClassify module (i.e., applied on various FCS data files) can be used to produce a compliant output.
No of labels (required)	The number of distinct labels (populations) to look for among all the input subpopulations. If you do not know how many distinct labels to look for, you can use the MClustClusterLabelBIC module to get an estimate.

Dimensions	A comma-separated list of dimensions (flow cytometry parameters/channels) used for computing the BIC. This list of dimensions should correspond to dimensions used for cluster labeling, which should be the same as the dimensions that have originally been used for clustering. The module accepts both: a list of parameter names (e.g., FSC-H, SSC-H, FL1-H, FL4-H) or a list of parameter indexes (e.g., 1,2,4,5,8). All specified dimensions must be included in all the input clustering results CSV files. All dimensions but Time will be used if the Dimensions parameter is not provided.
Model (required)	Specific model used for clustering of the means of pre-clustered data. Options are:<br>• EII – Spherical, equal volume.<br>• VII, default – Spherical, unequal volume.<br>• EEI – Diagonal, equal volume and shape.<br>• VEI – Diagonal, varying volume, equal shape.<br>• EVI – Diagonal, equal volume, varying shape.<br>• VVI – Diagonal, varying volume and shape.<br>• EEE – Ellipsoidal, equal volume, shape, and orientation.<br>• EEV – Ellipsoidal, equal volume and equal shape.<br>• VEV – Ellipsoidal, equal shape.<br>• VVV – Ellipsoidal, varying volume, shape, and orientation.<br>See MClust documentation (http://cran.r-project.org/web/packages/mclust/index.html) for details about different models.
Uncertainty. threshold (required)	The threshold (in percent) for maximum allowed uncertainty when assigning labels. Populations where no label can be assigned with probability greater or equal to the value of the Uncertainty threshold parameter will be marked as unassigned in the labels.csv result file. The value of the Uncertainty threshold parameter must be >50 and <100. Independently on this threshold, details about probability of each of the subpopulations having assigned each of the labels can be reviewed in the label probability.csv file. Default: 65

Output Files

1. Assigned labels

   A CSV file named labels.csv with two columns: Population file and Label. This file assigns labels (1, 2, 3, ...) for pre-clustered populations. A label 0 means that no label could be assigned considering the Uncertainty threshold parameter.

2. Label probability

   A CSV file named label probability.csv with columns: Population file, Label 1, Label 2, Label 3, etc. This file reports the probability (in percentages) of each of the labels being assigned to each of the pre-clustered populations. A label 0 means that no label could be assigned considering the Uncertainty threshold parameter.

3. Uncertainty

   A CSV file named uncertainty.csv with two columns: Population file and Label assignment uncertainty. This file reports the uncertainty (in percent) when assigning label for each of the pre-clustered populations.

Platform Dependencies

Module type: Flow Cytometry

CPU type: any

OS: any

Language: R (minimum version 2.10)

GenePattern Module Version Notes

Version	Description
1	Initial release 7/11/12.
