# Supplementary material for: GenePattern flow cytometry suite
Source: Source Code Biol Med. 2013 Jul 3;8:14. doi: 10.1186/1751-0473-8-14 (PMC3717030; doi:10.1186/1751-0473-8-14)
Supplement: Additional file 2 — GenePattern Flow Cytometry Suite Modules. Please use any ZIP-compatible software to extract the ZIP archive file into a folder. After extraction, the folder will contain 34 ZIP files, each of these represents one of the GenePattern modules in the GenePattern Flow Cytometry Suite. If you are hosting your own GenePattern server then you can install the GenePattern Flow Cytometry Suite from these module ZIP files by following “Modules & Pipelines”, “Install from zip” at your local GenePattern site. The latest version of these modules can always be obtained by navigating to the particular module at the GenePattern public server web site ( http://genepattern.broadinstitute.org/gp/) and following the “export” link. [file 1751-0473-8-14-S2.zip › AdditionalFile2_GPFCMSuiteModules/MClustClusterLabelBIC.zip!/MClustClusterLabelBIC.pdf]

GenePattern

MClustClusterLabelBIC Documentation

Description: Bayesian Information Criterion (BIC) for model-based labeling of clusters; helps estimate the number of labels for MClustClusterLabel.

Author: Josef Spidlen (jspidlen@bccrc.ca)

Please see the gp-flowcyt-help Google Group (https://groups.google.com/a/broadinstitute.org/forum/#!forum/gp-flowcyt-help) for help regarding these modules. If you have a GenePattern specific question, please feel free to contact GenePattern at gp-help@broadinstitute.org

Summary

Independent clustering of a several flow cytometry samples (e.g., blood from different patients) typically results in dividing each of the FCS files into several groups corresponding to cell subpopulations in each of the particular sample. Another analytical step is required to label these subpopulations so that they can be matched across different samples. The MClustClusterLabel module can be used for such a labeling. As one of the parameters, the MClustClusterLabel module requires the number of labels being set to the expected number of distinct labels (populations) to look for. The MClustClusterLabelBIC module can be used to estimate this number.

The MClustClusterLabelBIC module outputs the Bayesian Information Criterion (BIC) for model-based clustering of means of pre-clustered flow cytometry data for various (selected) models. Generally, the higher the BIC score, the better the appropriate model fits the data using the specified number of components. However, note that in certain cases, BIC may overestimate the number of components. At the beginning, the BIC curve usually tends to increase with increasing number of components up to a certain point, where it starts to decrease (sometimes temporarily). A common practice is to find the first inflection point and use it as the number of components for clustering.

The main input for the MClustClusterLabelBIC module is a set of CSV files with each subpopulation in a separate file. The columns of such an CSV file describe the flow cytometry parameters of the cells (in rows) of the CSV file. The FlowClustClassify module outputs its results in this kind of CSV file (on top of its CLS output). In addition, the user can specify a range of number of components (distinct labels) that can be searched for the best BIC value.

In addition, the search can be based all flow cytometry parameters (i.e., all but time, default) or a subset of these flow cytometry parameters can be specified. Searching based on a subset of flow cytometry parameters is useful in case the original clustering has been performed using such a subset and cluster labeling will be performed based on that subset as well. Finally, there are several models that may or may not be included when reporting the BIC score. You should include the model(s) that you are considering to use later for cluster labeling. See MClust documentation (http://cran.r-project.org/web/packages/mclust/index.html) for details about different models. At least one of these models shall be included in order for the algorithm to be able to search.

Usage

Memory and processing time was estimated based on processing a set 118 pre-clustered subpopulations (118 CSV files) with 5 flow cytometry parameters each (6 MB of input data) searching the range of 1-30 distinct labels.

- RAM: 30 MB
- Run time: 5 seconds

Requirements

- GenePattern ≥3.2.1
- R ≥2.10
- mclust ≥3.4.1
- workflowCommons ≥0.1-1

References

Chris Fraley and Adrian E. Raftery. MClust version 3 for r: Normal mixture modeling and model-based clustering, September 2006. http://www.stat.washington.edu/research/reports/2006/tr504.pdf

Parameters

Name	Description
Input.clustering.results (required)	The input CSV files with each subpopulation in a separate file. The column headings describe the flow cytometry parameters (i.e., parameter names). The rows of the CSV file include flow cytometry parameters of cells from a single sample assigned to a subpopulation by a preceding clustering step. For example, multiple runs of the moduleFlowClustClassify module (i.e., applied on various FCS data files) can be used to produce a compliant output.
Range	The range of distinct labels (populations) for which the module will compute the BIC. Default: 1-10
Dimensions	A comma-separated list of dimensions (flow cytometry parameters/channels) used for computing the BIC. This list of dimensions should correspond to dimensions used for cluster labeling, which should be the same as the dimensions that have originally been used for clustering. The module accepts both: a list of parameter names (e.g., FSC-H, SSC-H, FL1-H, FL4-H) or a list of parameter indexes (e.g., 1,2,4,5,8). All specified dimensions must be included in all the input clustering results CSV files. All dimensions but Time will be used if the Dimensions parameter is not provided.

EII<br>(required)	Include the EII model (spherical, equal volume) when calculating BIC. Use one of the following:<br>• Yes, default: Yes, include this model in the BIC report.<br>• No: No, omit this model from the BIC report.
VII<br>(required)	Include the VII model (spherical, unequal volume) when calculating BIC. Use one of the following:<br>• Yes, default: Yes, include this model in the BIC report.<br>• No: No, omit this model from the BIC report.
EEI<br>(required)	Include the EEI model (diagonal, equal volume and shape) when calculating BIC. Use one of the following:<br>• Yes: Yes, include this model in the BIC report.<br>• No, default: No, omit this model from the BIC report.
VEI<br>(required)	Include the VEI model (diagonal, varying volume, equal shape) when calculating BIC. Use one of the following:<br>• Yes: Yes, include this model in the BIC report.<br>• No, default: No, omit this model from the BIC report.
EVI<br>(required)	Include the EVI model (diagonal, equal volume, varying shape) when calculating BIC. Use one of the following:<br>• Yes: Yes, include this model in the BIC report.<br>• No, default: No, omit this model from the BIC report.
VVI<br>(required)	Include the VVI model (diagonal, varying volume and shape) when calculating BIC. Use one of the following:<br>• Yes: Yes, include this model in the BIC report.<br>• No, default: No, omit this model from the BIC report.
EEE<br>(required)	Include the EEE model (ellipsoidal, equal volume, shape, and orientation) when calculating BIC. Use one of the following:<br>• Yes: Yes, include this model in the BIC report.<br>• No, default: No, omit this model from the BIC report.
EEV<br>(required)	Include the EEV model (ellipsoidal, equal volume and equal shape) when calculating BIC. Use one of the following:<br>• Yes: Yes, include this model in the BIC report.<br>• No, default: No, omit this model from the BIC report.

VEV (required)	Include the VEV model (ellipsoidal, equal shape) when calculating BIC. Use one of the following:<br>• Yes: Yes, include this model in the BIC report.<br>• No, default: No, omit this model from the BIC report.
VVV (required)	Include the VVV model (ellipsoidal, varying volume, shape, and orientation) when calculating BIC. Use one of the following:<br>• Yes: Yes, include this model in the BIC report.<br>• No, default: No, omit this model from the BIC report.

Output Files

1. Suggested number of labels

   A file named *SuggestedNoOfLabels.txt* with an ASCII encoded integer value in it. The value in the file represents the suggested number of labels. This number is based on fitting the means of the pre-clustered input data to the VVI model (diagonal, varying volume and shape) and it suggests the number based on the first inflection point of the BIC score curve of the VII model. While we produce a simple numeric output in this file, it is recommended that rather than trusting this number, you visually inspect the produced BIC plots (file BIC plot.png) and estimate the best number yourself by looking at the curves of the model(s) that you are planning to use for further cluster labeling.

2. BIC plots

   BIC plots as a PNG image file in a file named *BIC_plot.png*. The image will include the BIC curve for the selected models and specified range of distinct labels (number of components). Visual inspection of the plot is the best way to estimate the resulting number of clusters. Typically, one looks at the curve and finds either the maximum or the first inflection point of the related BIC curve. Looking at various BIC curves may also suggest, which of the model is suitable to fit the pre-clustered data for the labeling purposes.

3. BIC results

   A CSV file named *BIC_results.csv* with columns representing the number of components and additional columns for BIC values of each of the models selected for output (for example EII BIC, VII BIC, etc). This file provides detailed BIC values for the selected models and specified range of distinct labels (number of components) and can therefore be used for more sophisticated computational estimates of the best number of labels and model to use for further cluster labeling. A value NA will be included if a specific model cannot be fitted for specific number of components and therefore, the BIC value cannot be computed.

Platform Dependencies

Module type: Flow Cytometry

CPU type: any

OS: any

Language: R (minimum version 2.10)

GenePattern Module Version Notes

Version	Description
1	Initial release 7/11/12.
