# Supplementary material for: GenePattern flow cytometry suite
Source: Source Code Biol Med. 2013 Jul 3;8:14. doi: 10.1186/1751-0473-8-14 (PMC3717030; doi:10.1186/1751-0473-8-14)
Supplement: Additional file 2 — GenePattern Flow Cytometry Suite Modules. Please use any ZIP-compatible software to extract the ZIP archive file into a folder. After extraction, the folder will contain 34 ZIP files, each of these represents one of the GenePattern modules in the GenePattern Flow Cytometry Suite. If you are hosting your own GenePattern server then you can install the GenePattern Flow Cytometry Suite from these module ZIP files by following “Modules & Pipelines”, “Install from zip” at your local GenePattern site. The latest version of these modules can always be obtained by navigating to the particular module at the GenePattern public server web site ( http://genepattern.broadinstitute.org/gp/) and following the “export” link. [file 1751-0473-8-14-S2.zip › AdditionalFile2_GPFCMSuiteModules/MergeFCSDataFiles.zip!/MergeFCSDataFiles.pdf]

MergeFCSDataFiles Documentation

Description: Merge multiple Flow Cytometry Standard (FCS) data files into a single FCS dataset; includes sub-sampling option.

Author: Josef Spidlen (jspidlen@bccrc.ca), gp-help@broadinstitute.org

Summary

This module will allow you to combine events from multiple FCS data files and save these in a single file as long as events in all the source files contain the same parameters. This may be useful in certain cases, for example, using clustering to identify all the possible subpopulations based on samples from several patients.

This module will take a list of FCS data files and output a single FCS data file containing events from all the specified input data files. The parameters in all the data files should match; this is verified based on parameter short names (the $PnN keyword). An error will be produced if parameters in the input FCS data files are inconsistent.

If subsampling rate is being used, each event has a probability approximately equal to the subsampling rate of being included in the result file; e.g., 50% of events are taken from each data set. Another option is to specify the approximate number of events that will be included from each dataset, e.g., 1000 random events are included from each data set. These two options cannot be combined. By default, all events are included in the result file.

For more information about the FCS file standard and its parameters, see this page at the International Society for Advancement of Cytometry.

Usage

Merging a set of 102 FCS files containing 7 data parameters and 10,000-38,000 events each (23 MB of input data) required 460 MB of RAM and a 13-second run time.

Merging a set of 48 FCS files containing 13 data parameters and 22,000-70,000 events each (142 MB of input data) required 1 GB of RAM and a 25-second run time.

References

Spidlen J, Moore W, Parks D, Goldberg M, Bray C, Bierre P, Gorombey P, Hyun B, Hubbard M, Lange S, Lefebvre R, Leif R, Novo D, Ostruszka L, Treister A, Wood J, Murphy RF, Roederer M, Sudar D, Zigon R, Brinkman RR. Data file standard for flow cytometry, version FCS 3.1. *Cytometry A*. 2010;77:97-100.

Parameters

Name	Description
Input.files (required)	The input FCS files to be merged. Use either a zip file or a pointer to a directory or a text file with FCS file names (full path) with one filename per line.
Subsampling. rate	The subsampling rate is the percentage of events to be included from each data set. The value of the subsampling rate is between 0 and 100. Default:100%<br>This parameter **cannot** be combined with the *Keep n events* option.<br>All events will be included if neither *Subsampling rate* nor *Keep n events* is specified.
Keep.n.events	Keep approximately *n* events from each dataset. This parameter cannot be combined with *Subsampling rate*.<br>All events will be included if neither *Subsampling rate* nor *Keep n events* is specified.
Random.seed	The random seed. Default: 1234567
Output.file.name	The output FCS file name. Default: MergedFCSDataFiles.fcs

Output Files

1. FCS data file

   This single FCS data file has one data set containing events from all the input FCS files. Depending on the *Subsampling rate* or *Keep n events* parameters specified, either all events or a portion of them will be included in this file.

Platform Dependencies

Module type: Flow Cytometry

CPU type: any

OS: any

Language: Java (1.6 minimum)

GenePattern

GenePattern Module Version Notes

Date	Version	Description
7/3/2012	v.1	
