# Supplementary material for: GenePattern flow cytometry suite
Source: Source Code Biol Med. 2013 Jul 3;8:14. doi: 10.1186/1751-0473-8-14 (PMC3717030; doi:10.1186/1751-0473-8-14)
Supplement: Additional file 2 — GenePattern Flow Cytometry Suite Modules. Please use any ZIP-compatible software to extract the ZIP archive file into a folder. After extraction, the folder will contain 34 ZIP files, each of these represents one of the GenePattern modules in the GenePattern Flow Cytometry Suite. If you are hosting your own GenePattern server then you can install the GenePattern Flow Cytometry Suite from these module ZIP files by following “Modules & Pipelines”, “Install from zip” at your local GenePattern site. The latest version of these modules can always be obtained by navigating to the particular module at the GenePattern public server web site ( http://genepattern.broadinstitute.org/gp/) and following the “export” link. [file 1751-0473-8-14-S2.zip › AdditionalFile2_GPFCMSuiteModules/PlateQAFCS.zip!/PlateQAFCS.pdf]

GenePattern

PlateQAFCS Documentation

Description: Perform automated quality assessment of plate-based Flow Cytometry Standard (FCS) data.

Author: Aaron Barsky, Josef Spidlen (jspidlen@bccrc.ca)

Please see the gp-flowcyt-help Google Group (https://groups.google.com/a/broadinstitute.org/forum/#!forum/gp-flowcyt-help) for help regarding these modules. If you have a GenePattern specific question, please feel free to contact GenePattern at gp-help@broadinstitute.org

Summary

This module performs quality assurance checking on a collection of FCS data files, under the assumption that all the samples should share similar properties in the forward scatter (FSC), side scatter (SSC), and time channels. A common use case is to ensure that a multiple-well plate of samples was correctly processed by the flow cytometer. The module calculates summary statistics on all the samples, and flags outliers in the report. The Web-based report can be viewed by clicking on the index.html file in the job results page.

For more information on the FCS file format, see the FCS 3.1 File Standard (PDF).

Usage

The time and memory requirements were evaluated based on the following tests:

- Quality assurance checking of 102 FCS files containing 7 data parameters and 10,000-38,000 events each (23 MB of input data). Required RAM: 680 MB. Run time: 9 minutes
- Quality assurance checking of 48 FCS files containing 13 data parameters and 22,000-70,000 events each (142 MB of input data). Required RAM: 1.7 GB. Run time: 7 minutes
- Quality assurance checking of 48 FCS files containing 13 data parameters and 90,000-120,000 events each (250 MB of input data).RequiredRAM:3.1 GB. Run time: 11 minutes

Requirements

- GenePattern ≥ 3.3.0
- R ≥ 2.10.0
- flowCore ≥ 1.12.0
- rflowcyt ≥ 1.18.0
- flowViz ≥ 1.10.0
- flowQ ≥ 1.6.0
- stats ≥ 4 2.10.0

References

Spidlen J, Moore W, Parks D, Goldberg M, Bray C, Bierre P, Gorombey P, Hyun B, Hubbard M, Lange S, Lefebvre R, Leif R, Novo D, Ostruszka L, Treister A, Wood J, Murphy RF, Roederer M, Sudar D, Zigon R, Brinkman RR. Data file standard for flow cytometry, version FCS 3.1. *Cytometry A*. 2010;77:97-100.

Parameters

Name	Description
Input directory (required)	FCS files from a single plate to be tested. The module accepts a ZIP file with all input files, or a server based directory name, or a text file listing all input files by their full server path (one input file per line).
Check cell counts (required)	Verify that the cell counts are similar for each sample. Default: Yes, default
Check medians (required)	Report the median values of the FSC and SSC channels. Default: Yes, default
Check PDF (required)	Report the probability density function of the FSC and SSC channels. Default: Yes, default
Check ECDF (required)	Report the empirical cumulative distribution function for the FSC and SSC channels. Default: Yes, default
Check boxplots (required)	Show the box plots for the FSC and SSC channels. A box plot shows the smallest observation, the lower quartile, the median, the upper quartile, and the largest observation. Default: Yes, default
Check timeline (required)	Plot the value of FSC against time. The resulting plot should show a horizontal line with random values above and below. A sloped line would indicate bias in the measurements. Default: Yes, default
Check timeflow (required)	Plot the count of events against time. Default: Yes, default

FSC channel (required)	The (short) name of the FSC parameter in all your data files, i.e., value of the $PnN keyword. If you don't know the name of the FSC channel, you can find it by running the PreviewFCS or ExtractFCSKeywords module. Default: FSC-H
SSC channel (required)	The (short) name of the SSC parameter in all your data files, i.e., value of the $PnN keyword. If you don't know the name of the SSC channel, you can find it by running the PreviewFCS or ExtractFCSKeywords module. Default: SSC-H
output file name (required)	Where the ZIP the output report to a single ZIP file. Default: TRUE

Output Files

1. Quality control report

   An HTML report summarizing the quality control tests. Depending on the Zip.output parameter, all the report files may be compressed into a single ZIP file named PlateQAFCSReport.zip, or they will be left as separate files. Please note that the report typically consists of hundreds of output files unless they are zipped together. A single ZIP may be easier to download; however, if files are not zipped together then the report may be viewed online on the server by opening the PlateQAFCSReport/report.html file. The report displays an overview table with rows corresponding to tested samples and rows to selected quality control processes. Clicking on the heading will show an appropriate overview plot for that process. Clicking on a + sign will expand the appropriate information. The colors in this report are chosen as follows:

   - green – test passed
   - yellow – there is a warning associated with the test
   - red – test failed

Platform Dependencies

**Module type:**	Flow Cytometry
**CPU type:**	any
**OS:**	any
**Language:**	R (2.10.0 minimum)

GenePattern Module Version Notes

Version	Description
1	Initial release 7/11/12.
