# Supplementary material for: GenePattern flow cytometry suite
Source: Source Code Biol Med. 2013 Jul 3;8:14. doi: 10.1186/1751-0473-8-14 (PMC3717030; doi:10.1186/1751-0473-8-14)
Supplement: Additional file 2 — GenePattern Flow Cytometry Suite Modules. Please use any ZIP-compatible software to extract the ZIP archive file into a folder. After extraction, the folder will contain 34 ZIP files, each of these represents one of the GenePattern modules in the GenePattern Flow Cytometry Suite. If you are hosting your own GenePattern server then you can install the GenePattern Flow Cytometry Suite from these module ZIP files by following “Modules & Pipelines”, “Install from zip” at your local GenePattern site. The latest version of these modules can always be obtained by navigating to the particular module at the GenePattern public server web site ( http://genepattern.broadinstitute.org/gp/) and following the “export” link. [file 1751-0473-8-14-S2.zip › AdditionalFile2_GPFCMSuiteModules/PreviewFCS.zip!/PreviewFCS.pdf]

PreviewFCS Documentation

Description: Allows viewing of structural metadata, parameters, and descriptive statistics from a Flow Cytometry Standard (FCS) data file

Author: Josef Spidlen (jspidlen@bccrc.ca),

Please see the gp-flowcyt-help Google Group (https://groups.google.com/a/broadinstitute.org/forum/#!forum/gp-flowcyt-help) for help regarding these modules. If you have a GenePattern specific question, please feel free to contact GenePattern at gp-help@broadinstitute.org

Summary

This module can be used to get a quick overview of the contents of an FCS data file. It provides a description including the number of events, number of parameters, and for each parameter, it provides its name (value of the $PnN keyword), full name (value of the $PnS keyword), minimum, maximum, median, mean, and first and third quartiles of the distribution. The format of the output can be either an HTML report or an XML document.

For more information on the FCS file format, see the FCS 3.1 File Standard (PDF).

Usage

Maximum memory and processing time was estimated based on processing an FCS file with 1,000,000 events and 24 parameters stored as FCS 3.0 in the floating point data type.

- Maximum RAM: 2 GB
- Maximum run time: 50 seconds

References

Spidlen J, Moore W, Parks D, Goldberg M, Bray C, Bierre P, Gorombey P, Hyun B, Hubbard M, Lange S, Lefebvre R, Leif R, Novo D, Ostruszka L, Treister A, Wood J, Murphy RF, Roederer M, Sudar D, Zigon R, Brinkman RR. Data file standard for flow cytometry, version FCS 3.1. *Cytometry A*. 2010;77:97-100.

Parameters

Name	Description
Input fcs data file	The input FCS file to be previewed.

Output file format	The file format to be used to show the preview output; options are:<br>• HTML (default) ; the report will be saved as a transitional XHTML version 1 file<br>• XML; the report will be saved as a transitional XML file
Output file name	The output file name. The default uses the input file name as the base. The file extension is determined by the output file format selected.

Output Files

1. Preview report
   Description of the contents of the FCS file in either HTML or XML. For each data set in the input FCS data file, this report includes the number of events, number of parameters, version of FCS used, data acquisition date and time, and data type used. For each parameter, it provides its name ($PnN keyword), full name ($PnS keyword), scale (linear or logarithmic), minimum, maximum, median, mean, and first and third quartiles of the distribution. In addition, the report contains all keyword/value pairs, as well as a spillover matrix if found in the file.

Platform Dependencies

Module type: Flow Cytometry

CPU type: any

OS: any

Language: Java (1.6 minimum)

GenePattern Module Version Notes

Date	Version	Description
7/13/2012	v.2	Fix for parsing empty keyword values
