# Supplementary material for: GenePattern flow cytometry suite
Source: Source Code Biol Med. 2013 Jul 3;8:14. doi: 10.1186/1751-0473-8-14 (PMC3717030; doi:10.1186/1751-0473-8-14)
Supplement: Additional file 2 — GenePattern Flow Cytometry Suite Modules. Please use any ZIP-compatible software to extract the ZIP archive file into a folder. After extraction, the folder will contain 34 ZIP files, each of these represents one of the GenePattern modules in the GenePattern Flow Cytometry Suite. If you are hosting your own GenePattern server then you can install the GenePattern Flow Cytometry Suite from these module ZIP files by following “Modules & Pipelines”, “Install from zip” at your local GenePattern site. The latest version of these modules can always be obtained by navigating to the particular module at the GenePattern public server web site ( http://genepattern.broadinstitute.org/gp/) and following the “export” link. [file 1751-0473-8-14-S2.zip › AdditionalFile2_GPFCMSuiteModules/RemoveSaturatedFCSEvents.zip!/RemoveSaturatedFCSEvents.pdf]

GenePattern

RemoveSaturatedFCSEvents Documentation

Description: Remove saturated events from an FCS data file.

Author: Josef Spidlen (jspidlen@bccrc.ca)
Please see the gp-flowcyt-help Google Group (https://groups.google.com/a/broadinstitute.org/forum/#!forum/gp-flowcyt-help) for help regarding these modules. If you have a GenePattern specific question, please feel free to contact GenePattern at gp-help@broadinstitute.org

Summary

The module takes an input FCS data file and removes saturated events, i.e., events with parameter values on (or very close to) the maximum range of particular scales. Very close means within 0.1%, i.e., an event is removed if 1.001 * event value is greater that the maximum range for that particular parameter. The result is saved as a new data file.

Saturated events are usually created when the voltage on the instrument is set too high so that event values fall outside of the recordable scale. These events are commonly recorded with the maximum allowed value, which may cause problems for subsequent analysis (for example, these outliers may create artificial clusters and cause problems for automated gating). Removal of these events may be beneficial for further analysis; however, it should be used carefully and the user should check how many and which events have been removed (see the standard output for these details).

Usage

Maximum memory and processing time was estimated based on processing an FCS file with 1,000,000 events and 24 parameters stored as FCS 3.0 in floating point data type.

- Maximum RAM: 2 GB
- Maximum run time: 40 seconds

Server Requirements

- GenePattern ≥ 3.2.0
- Java ≥ 1.6

References

- Josef Spidlen, Wayne Moore, David Parks, Michael Goldberg, Chris Bray, Pierre Bierre, Peter Gorombey, Bill Hyun, Mark Hubbard, Simon Lange, Ray Lefebvre, Robert R. Leif, David Novo, Leo Ostruszka, Adam Treister, James Wood, Robert F. Murphy, Mario Roederer, Damir Sudar, Robert Zigon, and Ryan R. Brinkman.

Data file standard for flow cytometry, version FCS 3.1. Cytometry A, 77(1):97–100, 2010.

Parameters

Name	Description
Output FCS file name	The name of the out FCS file.<br>The output file name will be constructed by concatenating the input file name with the “without_saturated” suffix the output FCS file name parameter is not given.

Input Files

1. Input FCS data file (required)

   The FCS data file to remove saturated events from.

Output Files

1. Output FCS data file

   An FCS data file with saturated events removed from the data set.

2. Standard output

   Text based information enumerating events that have been removed from the data set and stating the total number and percentage of events removed.

Example Data

Example data file ExampleInput.fcs has been included withing the module ZIP file and can be obtained by exporting the module and extracting the extracted ZIP file.

Platform Dependencies

**Module type:**	Flow Cytometry
**CPU type:**	any
**OS:**	any
**Language:**	Java (minimum version 1.6)

GenePattern Module Version Notes

Version	Description
1	Initial version released 7/11/12
