# Supplementary material for: GenePattern flow cytometry suite
Source: Source Code Biol Med. 2013 Jul 3;8:14. doi: 10.1186/1751-0473-8-14 (PMC3717030; doi:10.1186/1751-0473-8-14)
Supplement: Additional file 2 — GenePattern Flow Cytometry Suite Modules. Please use any ZIP-compatible software to extract the ZIP archive file into a folder. After extraction, the folder will contain 34 ZIP files, each of these represents one of the GenePattern modules in the GenePattern Flow Cytometry Suite. If you are hosting your own GenePattern server then you can install the GenePattern Flow Cytometry Suite from these module ZIP files by following “Modules & Pipelines”, “Install from zip” at your local GenePattern site. The latest version of these modules can always be obtained by navigating to the particular module at the GenePattern public server web site ( http://genepattern.broadinstitute.org/gp/) and following the “export” link. [file 1751-0473-8-14-S2.zip › AdditionalFile2_GPFCMSuiteModules/SamSPECTRALClusterFCS.zip!/aSamSPECTRALClusterFCS.pdf]

GenePattern

SamSPECTRALClusterFCS Documentation

Description: Clustering of flow cytometry data using the SamSPECTRAL algorithm.

Author: Josef Spidlen (jspidlen@bccrc.ca)

Please see the gp-flowcyt-help Google Group (https://groups.google.com/a/broadinstitute.org/forum/#!forum/gp-flowcyt-help) for help regarding these modules. If you have a GenePattern specific question, please feel free to contact GenePattern at gp-help@broadinstitute.org

Summary

Spectral clustering is a non-parametric clustering method which has proved useful in many pattern recognition areas. Not only it does not require a priori assumptions on the size, shape and distributions of clusters, but it has several features that make it an appropriate candidate for clustering biological data:

- It is not sensitive to outliers, noise or shape of clusters.
- It is adjustable so we can make use of biological knowledge to adapt it for a specific problem or dataset.
- There is mathematical evidence to guarantee its proper performance.

However, there are serious empirical barriers in applying spectral clustering for large data sets, such as commonly present in flow cytometry. TheSamSPECTRALClusterFCS module implements the SamSPECTRAL algorithm, which is a modification of spectral clustering allowing you to process flow cytometry data.

For more information on the FCS file format, see the FCS 3.1 File Standard (PDF).

Usage

Maximum memory and processing time was estimated based on clustering several larger FCS files. For files with more than 100,000 events, beta was set to 0. Please note that setting beta to 0 is essential to process data sets with more 100,000 events in order to obtain results in reasonable time.

- FCS file with 200,000 events, clustering 10 dimensions; setting sigma=100, separation factor=2 and beta=0: RAM: 500 MB; run time: 11 minutes.
- FCS file with 1,000,000 events, clustering 10 dimensions; setting sigma=100, separation factor=2 and beta=0: RAM: 1.3 GB; run time: 25 minutes.
- FCS file with 100,000 events, clustering 12 dimensions; setting sigma=200, separation factor=2 and beta=4: RAM: 425 MB; run time: 18 minutes.

GenePattern

Server Requirements

- GenePattern ≥ 3.2.0
- R 2.11.0

References

- Zare H, Shooshtari P, Gupta A, Brinkman RR. Data reduction for spectral clustering to analyze high throughput flow cytometry data. *BMC Bioinformatics*. 2010;11:403.
- Parks DR, Roederer M, Moore WA. A new ”logicle” display method avoids deceptive effects of logarithmic scaling for low signals and compensated data. *Cytometry A*, 2006;69:541–551.
- Spidlen J, Moore W, Parks D, Goldberg M, Bray C, Bierre P, Gorombey P, Hyun B, Hubbard M, Lange S, Lefebvre R, Leif R, Novo D, Ostruszka L, Treister A, Wood J, Murphy RF, Roederer M, Sudar D, Zigon R, Brinkman RR. Data file standard for flow cytometry, version FCS 3.1. *Cytometry A*. 2010;77(1):97–100.

Links

- Habil Zare and Parisa Shooshtari. R/bioconductor package samspectral: Identification of cell populations in flow cytometry data. http://www.bioconductor.org/packages/bioc/html/SamSPECTRAL.html
- GenePattern. The CLS file format. http://www.broadinstitute.org/cancer/software/genepattern/tutorial/gp_fileformats.html#cls
- Wikipedia. Comma-separated values; the csv file format description: http://en.wikipedia.org/wiki/Comma-separated_values

Parameters

Name	Description
Input FCS data file (required)	FCS file to be clustered.
Dimensions	A comma-separated list of dimensions (flow cytometry parameters/channels) that shall be used for computing clustering. The module accepts both, a list of parameter names (e.g., FSC-H, SSC-H, FL1-H, FL4-H) or a list of parameter indexes (e.g., 1,2,4,5,8). All dimensions but Time will be used if the Dimensions parameter is not provided.

Transformation<br>(required)	Which transformation to apply on the data prior clustering. Fluorescence channels are usually better visualized and clustered using a transformation. Usually, the better the data looks visually, the better also the clustering results of this module. However, please note that applying a transformation where a high curvature region of the transformation coincides with regions of non near zero density of events can also generate spurious populations. You can use one of the following:<br>• ASinH (Hyperbolic Arcus Sine), default: The ASinH transformation produces good results on most data.<br>• Logarithmic transformation: The logarithmic transformation can be used of not too much data (or no data of interest) is located around the axes.<br>• Logicle transformation: Logicle transformation 2 is an alternative to logarithmic transformation that better handles data around the axes.<br>• No transformation: The data will be used as stored in the FCS data file.<br>Default: ASinH
Dimensions to transform	A comma-separated list of dimensions (channels) that shall transformed as specified by previous parameter. This will be ignored if no transformation is specified above. If this parameter is not provided and transformation is specified above, the algorithm will use heuristics to identify parameters that shall be transformed. These heuristics are based on how parameters are stored in the FCS file, their resolution and their name. Again, you can use either parameter names or parameter indexes to specify dimensions to transform.
Sigma<br>(required)	Sigma is a scaling parameter that determines the resolution in the spectral clustering stage; typically a value between 10 and 5000. By increasing it, more spectral clusters are identified. This can be useful when small population are aimed. See SamSPECTRAL documentation on more details how Sigma can be adjusted.
Separation factor<br>(required)	The separation factor is a threshold that controls to what extend clusters should be combined or kept separate; typically a value between 0.05 and 5. See SamSPECTRAL documentation on more details how the separation factor can be adjusted.

Beta (required)	The size of neighborhood that is being searched during the conductance calculation step. Beta can be set to a value from 0 to 5; however, the default value of 4 should not be changed unless the input data set contains measurements of more that 100,000 particles. Setting Beta to zero or a low value will reduce computational time by applying an approximation to the conductance calculation step. This will allow you to cluster large data sets in reasonable time; however, the quality of the result may be affected. Setting beta to 5 is not recommended since it will increase computational time while not significantly improving clustering results in most cases.<br>Default: 4, default
Seed	Seed value for the random generator; provide any consistent value to obtain reproducible results.

Input Files

1. Input FCS data file (required)
   The FCS file to be clustered, i.e., events/cells automatically separated into subpopulations.

Output Files

1. Subpopulations in separate CSV files

   The module outputs several CSV files, one for each of the identified cell subpopulation. The measurements in these files correspond to cells assigned to the particular population. The columns of the CSV file correspond to the parameters of the input FCS file and the column headings will be created based on the short and long parameter names ($PnN and $PnS keyword values) as a single name separated by ":", i.e., $PnN:$PnS, for example: "FL2-H:CD69 PE". The file names will be constructed as Input FCS file name_population_n.csv, where Input FCS file name is the name of your input file, and n is a number from 0 to the number of populations identified in the input FCS files. The population numbered as 0 lists unassigned cell measurements (i.e., identified as outliers).

2. CSV clustering results

   A clustering results file in the CSV format, which will store the population number for each event in a single file. The CSV file will contain a single column with the Label (0 is outlier) heading. Rows in the file will assign population labels (numbers) for events in the input FCS data file maintaining the same order of events as in FCS file. The population numbers are from 0 to the number of populations identified in the input FCS files. The population numbered as 0 lists unassigned cell measurements (i.e., identified as outliers).

3. CLS clustering results

   A clustering results file in the CLS format, which will store the population number for each event in a single file. The order of the events is the same as in the original FCS file. The population numbers are from 0 to the number of populations identified in the input FCS files. The population numbered as 0 lists unassigned cell measurements (i.e., identified as outliers).

Example Data

An example data file named ExampleInput.fcs can be obtained by exporting the module and extracting the zip file. You may use Sigma = 100, Separation factor = 0.1 and leave all the other parameters as defaults.

Platform Dependencies

**Module type:**	Flow Cytometry
**CPU type:**	any
**OS:**	any
**Language:**	R version 2.11

GenePattern Module Version Notes

Version	Description
1	Initial release 7/11/12.
