# Supplementary material for: GenePattern flow cytometry suite
Source: Source Code Biol Med. 2013 Jul 3;8:14. doi: 10.1186/1751-0473-8-14 (PMC3717030; doi:10.1186/1751-0473-8-14)
Supplement: Additional file 2 — GenePattern Flow Cytometry Suite Modules. Please use any ZIP-compatible software to extract the ZIP archive file into a folder. After extraction, the folder will contain 34 ZIP files, each of these represents one of the GenePattern modules in the GenePattern Flow Cytometry Suite. If you are hosting your own GenePattern server then you can install the GenePattern Flow Cytometry Suite from these module ZIP files by following “Modules & Pipelines”, “Install from zip” at your local GenePattern site. The latest version of these modules can always be obtained by navigating to the particular module at the GenePattern public server web site ( http://genepattern.broadinstitute.org/gp/) and following the “export” link. [file 1751-0473-8-14-S2.zip › AdditionalFile2_GPFCMSuiteModules/SetFCSKeywords.zip!/SetFCSKeywords.pdf]

SetFCSKeywords Documentation

Description: Sets keyword/value(s) in a Flow Cytometry Standard (FCS) file.

Author: Josef Spidlen (jspidlen@bccrc.ca)

Please see the gp-flowcyt-help Google Group (https://groups.google.com/a/broadinstitute.org/forum/#!forum/gp-flowcyt-help) for help regarding these modules. If you have a GenePattern specific question, please feel free to contact GenePattern at gp-help@broadinstitute.org.

Summary

Specific metadata information (data about data) can be stored in FCS data files as part of the TEXT segment in the form of keyword/value pairs. For example, this may include date and time of data acquisition, description of measured cells, comments, type and/or serial number of cytometer used, name of the operator, tube or plate identification, excitation wavelength and power for measured channels, types and voltages of detectors used, identification of the sample/specimen and its source, volume of the sample run during data acquisition, or any other custom information.

This module can be used to place additional metadata in an FCS file or replace existing metadata stored in a keyword/value pair. The keyword/values can be submitted as a CSV file with two columns (Keyword and Value) or as a text file with a chunk of FCS TEXT segment. An error will be reported if a specified keyword value cannot be set, e.g., it would break the consistency of the FCS data file.

For more information on the FCS file format, see the FCS 3.1 File Standard (PDF).

FCS TEXT Segment Format Details

The FCS TEXT segment contains a series of ASCII-encoded keyword/value pairs. The delimiter is the first character of the TEXT segment chunk and this delimiter is subsequently placed to separate keywords from keyword values. The delimiter can be any ASCII character. For example, "$P1S/Forward scatter/" is a keyword/value pair. In this example, the '/' is the delimiter character. The delimiter is placed at the start and end of each keyword value. The delimiter may not be the first character in a keyword or keyword value. If the delimiter appears in a keyword or keyword value, it must be immediately followed by a second delimiter. For example, "$SYS/RSX-11//M/" shows a value of RSX-11/M for the $SYS keyword. Since null (zero length) keywords or keyword values are not permitted, two consecutive delimiters can never occur between a value and a keyword.

Example of a TEXT segment chunk:

> /My Keyword/My Value/My Other Keyword/My other value containing a // delimiter character/$COM/Example/

Please note that the delimiter character always appears both at the beginning and at the end of the FCS TEXT segment chunk. See the FCS 3.1 File Standard (PDF) for more details.

Usage

Maximum memory and processing time was estimated based on adding 10 keywords to an FCS file with 1,000,000 events and 24 parameters stored as FCS 3.0 in the floating point data type.

- Maximum RAM: 1.8 GB
- Maximum run time: 30 seconds

References

Spidlen J, Moore W, Parks D, Goldberg M, Bray C, Bierre P, Gorombey P, Hyun B, Hubbard M, Lange S, Lefebvre R, Leif R, Novo D, Ostruszka L, Treister A, Wood J, Murphy RF, Roederer M, Sudar D, Zigon R, Brinkman RR. Data file standard for flow cytometry, version FCS 3.1. *Cytometry A*. 2010;77:97-100.

Parameters

Name	Description
Input FCS data file	The input FCS data file in which to set keyword/value pairs.
Keywords input file	A file with keyword/value pairs to be added to the input FCS data file. The keyword/value pairs can be submitted as a CSV file with two columns: *Keyword* and *Value* (though the heading is optional). They can also be submitted as a text file with a chunk of FCS TEXT segment, such as "/$P3S/MUC 1 APC/$P4S/Thy1 PE/BD$CUSTOM_STUFF/12/".
Output FCS file name	The file name for the output FCS data file.

Output Files

1. FCS data file with keyword/value pairs added

   FCS data file containing all events and parameters from the input data file with additional keyword/value pairs added and/or values of existing keywords updated according to the specified Keywords input file.

Platform Dependencies

Module type: Flow Cytometry

CPU type: Any

OS: Any

Language: Java (1.6 minimum)

GenePattern Module Version Notes

Date	Version	Description
07/11/12	v.2	SetFCSKeywords v.2 Fix for parsng empty keyword values.
