# Supplementary material for: GenePattern flow cytometry suite
Source: Source Code Biol Med. 2013 Jul 3;8:14. doi: 10.1186/1751-0473-8-14 (PMC3717030; doi:10.1186/1751-0473-8-14)
Supplement: Additional file 2 — GenePattern Flow Cytometry Suite Modules. Please use any ZIP-compatible software to extract the ZIP archive file into a folder. After extraction, the folder will contain 34 ZIP files, each of these represents one of the GenePattern modules in the GenePattern Flow Cytometry Suite. If you are hosting your own GenePattern server then you can install the GenePattern Flow Cytometry Suite from these module ZIP files by following “Modules & Pipelines”, “Install from zip” at your local GenePattern site. The latest version of these modules can always be obtained by navigating to the particular module at the GenePattern public server web site ( http://genepattern.broadinstitute.org/gp/) and following the “export” link. [file 1751-0473-8-14-S2.zip › AdditionalFile2_GPFCMSuiteModules/SuggestNumberOfPopulationsFCS.zip!/aSuggestNumberOfPopulationsFCS.pdf]

GenePattern

SuggestNumberOfPopulationsFCS Documentation

Description: Suggest a number of clusters (K value) for a clustering algorithm.

Author: Aaron Barsky, Josef Spidlen (jspidlen@bccrc.ca)

Please see the gp-flowcyt-help Google Group (https://groups.google.com/a/broadinstitute.org/forum/#!forum/gp-flowcyt-help) for help regarding these modules. If you have a GenePattern specific question, please feel free to contact GenePattern at gp-help@broadinstitute.org

Summary

Most clustering algorithms require the user to input the number (K) of expected sub-populations. This module can help if you do not know how many populations to look for. It tests a range of several K values and suggests a number of populations using the Bayesian Information Criterion (BIC) in combination with the FlowClust algorithm. In addition, it outputs the estimated BIC as well as the Integrated Completed Likelihood (ICL) score for each of the K values, which may also help to select an appropriate number of populations.

For more information on the FCS file format, see the FCS 3.1 File Standard (PDF).

Usage

Maximum memory and processing time was estimated based on processing an FCS file with 1,000,000 events and 21 parameters; the range was kept at default, i.e., algorithm asked to search for the best number of clusters between 1-30; however, the effective search was performed for 1-9 clusters since more than 9 clusters could not be found using the FlowClust algorithm. An Intel(R) Core(TM)2 Extreme X9650 CPU at 3.00GHz has been used.

- Maximum RAM: 2.3 GB
- Maximum run time: 25 hours

Requirements

- GenePattern ≥ 3.2.1
- R 2.10.0
- flowCore ≥ 1.12.0
- flowClust ≥ 2.4.0

References

Lo K, Brinkman RR, Gottardo R. Automated gating of flow cytometry data via robust model-based clustering. *Cytometry A*. 2008;73:321-332.

Parameters

Name	Description
Input FCS data file	The FCS file to be examined to determine the number of populations.
Dimensions	A comma-separated list of long parameter names ($PnS values) to be used in the analysis. All parameters will be used if no value for the Dimensions parameter is provided. The Time parameter is excluded automatically.
Range	The range of K values to be tested. The range will be specified in the form of “min,max ”. If Range is not specified, the module will try all K values from 1 to 30. Using the Range parameter, you can specify a smaller or larger range. Note that FlowClust may not be able to correctly cluster data with very high K values, in which case scores will be computed up to the highest valid K value.

Output Files

1. Suggested number of populations

   A single suggested number of populations will be stored in the suggestedNumberOfPopulations.txt file. This value is based on the estimated BIC score.

2. BIG score plot

   A plot of the Bayesian Information Criteria (BIG) score for each of the tested K values will be saved in the BICScorePlot.png file.

3. ICL score plot

   A plot of the Integrated Completed Likelihood (ICL) score for each of the tested K values will be saved in the ICLScorePlot.png file.

Platform Dependencies

Module type: Flow Cytometry

CPU type: any

OS: any

Language: R 2.10.0

GenePattern

GenePattern Module Version Notes

Version	Description
1	Initial release 7/13/12
